# Probing biofilm development, stress response and heterogeneity—spectroscopic characterization of single and multi-species consortia

**DOI:** 10.1038/s41522-026-01010-x

**Published:** 2026-05-20

**Authors:** Elena Yunda, Aleksandra Hagberg, Thibault Duteil, Grégory Francius, András Gorzsás, Fabienne Quilès, Madeleine Ramstedt

**Affiliations:** 1 Department of Chemistry, Umeå University, Umeå, Sweden; 2 Umeå Centre for Microbial Research, Umeå University, Umeå, Sweden; 3 Université de Lorraine, CNRS, LCPME, Nancy, France; 4 Science for Life Laboratory, Umeå University, Umeå, Sweden

**Keywords:** Biofilms, Water microbiology, Microbial communities

## Abstract

Environmental bacterial biofilms play many roles in the ecosystem including cycling of nutrients and serving as food for grazing organisms. Their function is linked to their microbial and chemical composition that may be altered by several parameters including environmental stressors. This manuscript presents a well-characterized model system of four bacterial isolates from a small Swedish river: *Pseudomonas* sp., *Sphingomonas* sp., *Rhizobium* sp. and *Pararhizobium* sp. Microbiological and chemical phenotypes were investigated including cell and biofilm morphology, as well as biochemical composition in absence and presence of the drug trimethoprim. Vibrational spectroscopy, cryo-X-ray photoelectron spectroscopy and confocal optical microscopy were applied to investigate and characterize monocultures and cocultures. The chemical characterization showed variation of the energy storage substance polyhydroxyalkanoates as well as polysaccharides between isolates and drug exposures. Spatial heterogeneities were observed using Raman microspectroscopy where *Sphingomonas* sp. cells, formed small clusters, inside the four species consortium, an organization that appeared to protect this isolate during exposure to trimethoprim.

## Introduction

Bacteria live in different types of environmental niches of our planet. They have been found in large varieties in waters, soils and biota and have adapted their lifestyle to the specific life conditions present in their microenvironments^1–3^. In each environmental niche, microbial communities form an important part of the ecosystem and are key actors for the biogeochemical cycling of important nutrients, for example as part of the food chain for other organisms and as degraders of organic matter^4^. Bacteria have several lifestyles: planktonic (free-swimming) cells, or communities called biofilms. Biofilm consists of cells encapsulated in extracellular polymeric material (EPS) and represents a microbial life form that shields bacteria from environmental stress such as dehydration or exposure to toxic substances^5–7^. The extracellular material in biofilms may consist of a range of different substances. Common macromolecules encountered in EPS are polysaccharides, proteins, extracellular DNA, and enzymes^8^. These macromolecules provide the EPS and the biofilm with a range of specific physicochemical properties, such as water retention, adhesion to substrates, rigidity and reduced diffusion of extracellular material such as enzymes or membrane vesicles from the biofilm^9–11^. The EPS can also function as a nutrient reserve and help bacteria to survive in an environment that may have fluctuating access to nutrients and water^2^. Biofilms facilitate communication and collaboration between microorganisms residing inside it and provide an arena that can enhance synergistic multi-species interactions^12,13^. This lifestyle provides many benefits for survival, growth and coping with environmental stressors^12,13^. Thus, in the environment, multi species biofilms are often observed and probably represent one of the most common lifestyles for environmental bacteria.

The presence of pharmaceuticals in the environment is an emerging problem in various ecosystems^14,15^. These substances may be biologically active even at low concentrations, potentially influencing a wide range of organisms^14^. Their presence in the environment may serve as an environmental stress factor for organisms leading to changes in behavior, metabolism, survival, etc^14,16,17^. Such changes have been described for birds, mammals, fish, insects^14,17,18^, and microorganisms^19–22^. Previous studies show that planktonic bacteria and bacterial biofilms may respond to pollution on several levels and in different ways: they may accumulate pharmaceuticals^23,24^, alter their genetic code and develop resistance to a drug^1,22^, increase their tolerance by metabolic alterations^24^, increase production of energy reserve substances^25^, change their metabolic activity to become dormant^26^, alter the composition of secreted EPS^27^ or change the species composition in biofilms formed by multi-species consortia^24,28^. All these alterations of bacteria and biofilms can have broader implications on an ecosystem, including changes of nutrient composition of the biofilm affecting grazing organisms^29,30^, selective pressure on microbial communities altering their contribution to the overall biogeochemical cycling of nutrients^1,31^, as well as transfer of acquired antibiotic resistance genes from benign bacteria to opportunistic pathogens^1^.

Trimethoprim is a drug targeting the folate metabolism, affecting a number of metabolic pathways and thereby influencing essential metabolites in bacteria, including the synthesis of nucleic acids^32^. It is commonly used for treating urinary tract infections^32^. Trimethoprim has a pKa value of 7.4 and therefore is present both in protonated and non-protonated form at pH 7, whereas the positively charged form dominates at pH 5^33^. Protonation occurs at the N of the pyrimidine ring because of its higher electron density compared to the amino groups^34^. Protonation has previously been reported to reduce diffusion and uptake into bacterial cells^32,33^ due to an increase in charge and hydrophilicity^34^. However, positively charged trimethoprim molecules may be electrostatically attracted to bacterial cells, which are often negatively charged. Thus, the positively charged form may be preferentially bound to the outside of bacterial cells or EPS, even if its uptake is reduced. This may potentially affect different processes at the surface of the bacterial cell and possibly influence the properties of the EPS surrounding the cells. Previous work has shown that trimethoprim is not highly toxic to bacterial cells^35^. The half maximal effective concentration (EC50) for a range of bacteria has been reported in the range of mg/L, which is the range that is reached in patient fluids. However, it has been noted that many environmental bacterial have much lower threshold to this drug^35^. Furthermore, bacteria may be influenced by the drug at much lower concentrations than the effective killing dose, giving rise to phenotype alterations and resistance development^36^. In surface waters in Europe a range of trimethoprim concentrations has been monitored, 0.01–0.35 μg/L^37^. The predicted levels for Western Europe generally have been given as lower than 0.25 μg/L^35,37^. However, in waste water discharge and in global hotspots, concentrations have been reported up much higher and in some cases up to mg/L^36^.

Previous research on bacterial responses to toxins have shown that monospecies and multispecies biofilms may respond differently to the exposure and that bacteria often benefit from living in co-cultures^13^. The increased fitness in multispecies biofilms depends on a range of factors, including mutation, shared goods and metabolic specialization^12,38,39^. It has also been described that bacterial interactions may depend on the architecture of the biofilm and the internal organization. Thus, co-localization of bacteria may enhance sharing of goods and the development of gradients inside biofilms may produce niches where different bacteria may settle in an optimal environment. For example, with respect to antibiotics, more sensitive bacteria may survive better inside biofilms where they are protected by layers of EPS and other cells^12,40^. Thus, previous work has shown the importance of studying bacterial responses in model systems of higher complexity than monospecies biofilms.

Natural river biofilms are highly dynamic structures that vary depending on a range of factors such as water flow, nutrient flux, temperature variations, sun exposure, flux of pollutants, organism composition, grazing of other organisms, etc^19,41^. All these individual influences combine and affect natural biofilms. This results in biofilm systems of such high complexity that reproducibility, in practical terms, is lost^41^. Therefore, the research on environmental biofilms generally follows two methodologically different strategies that probe different aspects with respect to complexity and reproducibility^41^. The first approach aims to preserve the full complexity of natural biofilms by performing measurements out in the field or trying to sample and preserve the community structure in the lab. This approach is close to what is observed in the environment but is less suitable for understanding detailed processes and decipher mechanisms inside biofilms. Thus, to probe specific mechanisms and processes that may occur in environmental biofilms in a controlled and reproducible way, the complexity of the system has to be reduced, and model systems developed. This is the second approach, which aims to simplify the system in order to gain control over parameters influencing it, which in turn increases reproducibility and allows for more detailed mechanical insights. However, simplified models cannot represent every aspect of the original environmental system. For progress in the research area of environmental biofilms, both strategies are needed and ideally shine light on different types of research questions, thus, complementing each other.

This study is a part of a long-term research initiative, following the second research strategy described above, i.e., understanding specific processes in biofilms. The specific aim of this particular study was twofold: (I) to develop an environmentally relevant model system and (II) test it using the antibiotic trimethoprim as a model stressor. The concentrations of trimethoprim used are higher than the background concentrations found in the site of bacterial isolation. Thus, the exposure used here serves as an indication of possible effects from exposures from for example accidental pollution via waste water discharge. In order to develop an environmentally relevant model system for biofilms, several targets should be met. First, the organisms need to be sourced from a suitable environmental niche in order to be relevant and have predictive power for the research question at hand. Second, the organisms need to exhibit traits that are relevant both with respect to biological and chemical phenotypes. For example, the chosen strains must be cultivable in vitro both as monocultures and as co-cultures. Furthermore, they should exhibit chemical and physicochemical traits representative to what may be expected in the environmental niche studied. The system formed should also be robust enough to allow for investigations of both stationary and dynamic processes in order to capture several different types of processes and study how they respond to various perturbations. For a model system to be widely applicable it should also be constructed from a data-driven approach rather than a hypothesis driven approach, as the latter may restrict what types of research questions could be addressed using the model system. For the model system developed here, the overall context is investigations of specific fundamental responses to environmental stressors arising in fresh-water bacteria and biofilms. These responses may alter both bacterial cells and the EPS surrounding the cells in the biofilm, as well as influence spatial organization altering the microclimate inside the biofilm. This organization can take many forms. It has been described that cells may organize into gradients where one type consumes substances that are detrimental for others, thereby providing a fitness advantage for the more sensitive cells. Furthermore, sharing of goods such as genetic material is facilitated by close proximity between cells^40,42^. Sensitivity to toxins such as antibacterial substances have also been described to be reduced in heterogeneous biofilms and tightly linked to the spatial organization of cells inside biofilms^43^.

To study biofilm heterogeneities, experimental methodologies that can monitor both biofilm cells and their EPS are needed, in addition to well-controlled model systems. In this work, we have applied optical microscopy and vibrational spectroscopy techniques to probe model biofilms of environmental relevance both with respect to their chemistry and organization. Vibrational spectroscopic techniques, such as Fourier transform infrared (FTIR) and Raman spectroscopies, are non-destructive tools that can study bacterial biofilms and EPS, in situ, in real time, and without the use of external agents (labels, dyes or markers)^8^. Importantly, these techniques do not require a priori knowledge of the system, or its components, and they monitor the entire chemical composition simultaneously. Thus, they are data-driven rather than hypothesis-driven. In combination with multivariate analysis^44^, a multitude of processes can be studied in parallel in biological samples. In the context of microbiology, vibrational spectroscopy has been shown to e.g., differentiate between planktonic cells and biofilms, provide spatially resolved chemical information of both EPS and microorganisms, or between organisms^45^, follow biofilm development in real time in situ, as well as study how antimicrobial agents affect the biofilm chemistry^44,46,47^. Here, vibrational spectroscopy was combined with optical microscopy to give a detailed characterization of the bacterial model system developed, and to understand fundamental aspects about the internal organization inside co-cultures of these environmental bacteria. In a parallel study vibrational spectroscopy was used, in combination with other techniques, to study alteration in secreted EPS after trimethoprim exposure^27^.

This study presents the establishment of a well-characterized consortium of river bacteria that we propose as a model system for studying the effect of environmental stressors on bacteria. As a first step, four environmental bacterial isolates collected from a small river in Sweden, Knivstaån^28^, were genome sequenced. Secondly, they were carefully characterized with respect to various phenotypes such as cell morphology, growth, biofilm formation, motility and sensitivity to the drug trimethoprim. Spectroscopy was used to investigate the chemical composition of the bacterial cultures and of the biofilms formed. Thereafter, a four-species biofilm model was constructed and its architecture, organization and sensitivity to the selected drug was investigated, as a proof-of concept for the established model system.

## Results

The genome analyses of the four river isolates showed that they were closely related to other environmental isolates in the NIH GenBank database (for complete genome sequences, please refer to Supplementary Information). The genome from the *Pseudomonas* sp. isolate had the highest similarity to chromosomal DNA from an environmental *Pseudomonas* sp. isolate collected in China (GenBank CP117439.1), and also scored high on a number of strains belonging to the *Pseudomonas fluorescens* subgroup^48,49^. The *Sphingomonas* sp. isolate was most closely resembling environmental *Sphingomonas* sp. isolates from Korea (GenBank CP039249.1), Japan (GenBank AP022673.1) and a *Sphingomonas aerolata* isolate (Genbank CP098762.1) from France, in order of falling sequence similarity. *S. aerolata* is described to have orange color, similar to our isolate^50^. The *Rhizobium* sp. isolate had highest sequence similarity to chromosomal DNA from an environmental *Rhizobium* sp. isolated in the Shulgan-tash cave in Russia (GenBank CP087974.1). The fourth strain had highest sequence similarity to chromosomal DNA of an environmental *Pararhizobium sp*. strain isolated in Canada (Genbank CP149510.1). These isolates will, hereafter, be described only by their genus name.

### Growth, morphology and motility in individual isolates

**Fig. 1:**
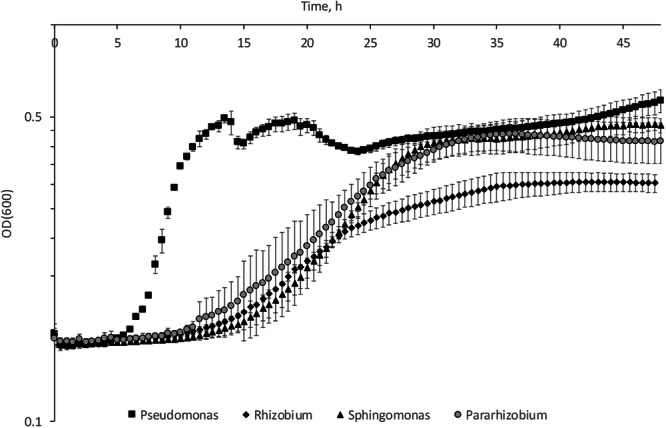
Growth curves in 100% R2A medium at room temperature (20 °C) for the four river isolates collected in the Knivsta River in the south of Sweden. Each measurement point is an average of optical densities at 600 nm (OD(600) measured in 6 wells in a 96-well plate, error bars represent the standard deviation, y-axis shows log10 scale. The data contains scatter from both planktonic cells and biofilm inside the wells. Squares represent *Pseudomonas*, diamonds *Rhizobium*, triangles *Sphingomonas* and circles *Pararhizobium*.

Bacterial growth in liquid medium gave rise to higher culture densities for the *Pseudomonas, Pararhizobium* and *Sphingomonas* isolates, than for the *Rhizobium* isolate over a time period of 48 h in 100% R2A medium (Fig. 1). Some variation in maximum optical density could be observed between biological replicates of *Pararhizobium* and *Sphingomonas* probably originating from small differences in expression of EPS influencing the optical density measurements (Supplementary Fig 2). The period for the lag phase was shorter for *Pseudomonas* than for the other strains (Fig. 1). This isolate also exhibited a very interesting and reproducible growth pattern with oscillating growth following the first exponential phase. An explanation for these oscillations could be dynamic biofilm formation influencing the scattering of light during the assay. The collection of data for the growth curves were done using an automated system enabling stationary growth followed by short instances (10 s) of shaking. Consequently, the collected data represent a sum of scatter from both biofilms formed in the wells and planktonic cells.

**Fig. 2:**
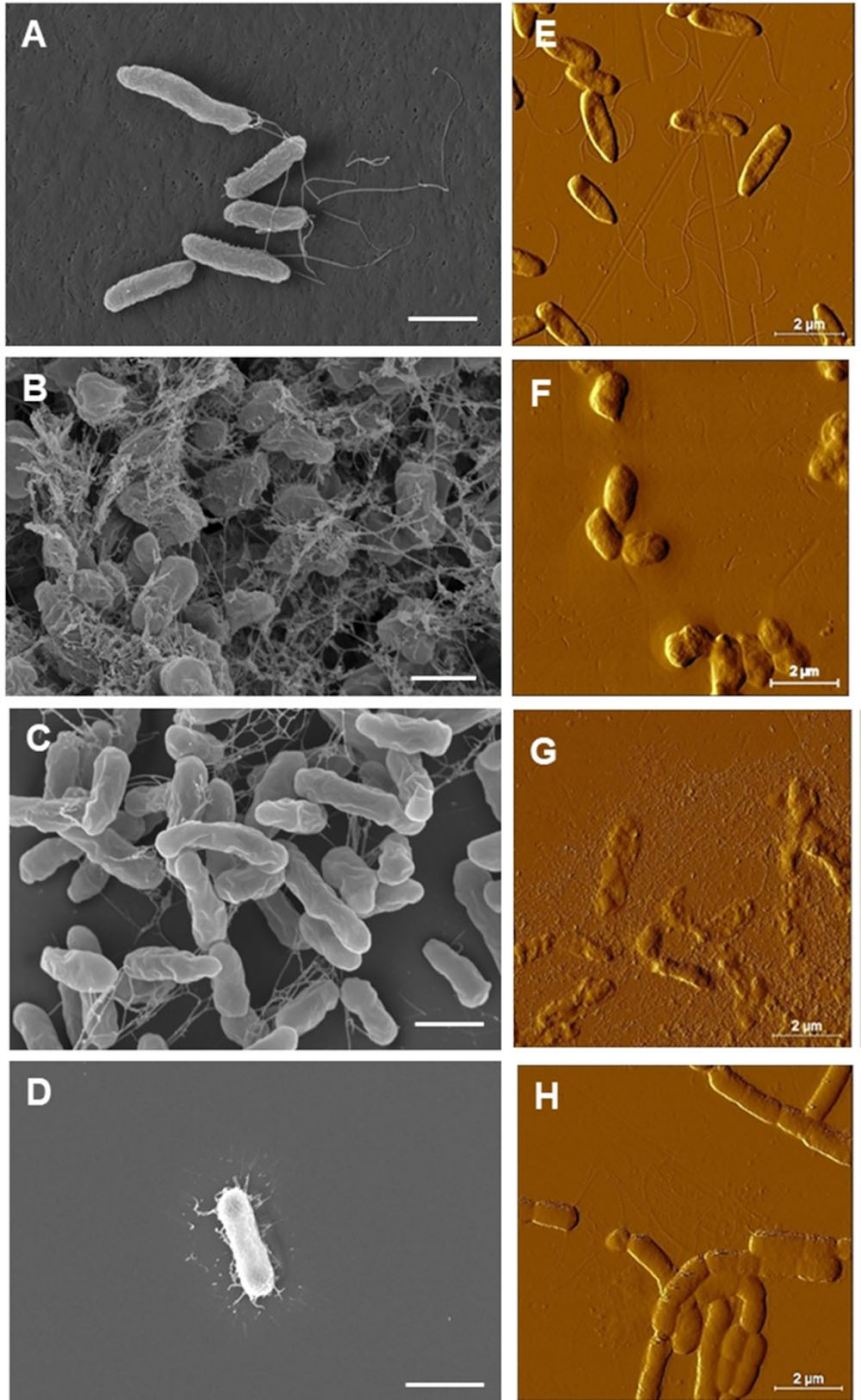
Cell morphology of river isolates. **A**–**D** Scanning electron microscopy and **E**–**H** atomic force microscopy deflection images showing the cell morphology for the four river isolates. **A**, **E** *Pseudomonas*, **B**, **F** *Pararhizobium*, (**C**, **G**) *Rhizobium*, (**D**, **H**) *Sphingomonas*. Scale bar is 1 µm in (**A**–**D**) and 2 µm in (**E**–**H**). Dehydrated extracellular material is visible as fibrous material in the SEM images of all strains except *Pseudomonas*.

All strains, except *Pseudomonas*, visibly produced substantial amounts of hydrated biomass when grown on culture plates (Supplementary Fig 3). Clear colonies were generally not observed but rather continuous hydrated material, indicating the presence of large amounts of extracellular material. This large EPS production could also be observed by eye in liquid cultures of *Sphingomonas* and the relative content of EPS to cells increased in low nutrient conditions (10% R2A medium vs 100% R2A). The EPS matrix could be observed as (dehydrated) material surrounding individual cells in scanning electron microscopy (SEM) images collected from cultures grown on agar plates (Fig. 2 and Supplementary Fig 4). The images showed that all bacteria were rod-shaped. *Pseudomonas* cells were 1.2 ± 0.2 μm long and 0.41 ± 0.03 μm wide and showed presence of polar flagella (Fig. 2A and Supplementary Fig. 4). *Pararhizobium* belongs to the same family (*Rhizobiaceae*) as *Rhizobium* but nowadays accounted for as a separate species. *Pararhizobium* cells have been described to be 1.2–2.5 μm long and 0.3–0.9 μm wide^51^. Our *Pararhizobium* isolate fit this description and showed a short, compact cell shape with a length at 1.6 ± 0.4 and a width of 0.55 ± 0.05 μm (Figs. 2B and Supplementary Fig. 4). Presence of flagella for *Pararhizobium* was difficult to elucidate in the images due to the large amounts of EPS. Cells of *Rhizobium* were without flagella and had a slenderer cell shape compared to the *Pararhizobium* isolate. They had a diameter of 0.48 ± 0.04 μm and a length of 1.8 ± 0.4 μm. This also corresponds well to previous reports for this species^52^. *Sphingomonas* morphology and size were also in agreement with literature values^53^ with a length of 1.4 ± 0.2 μm and a width of 0.46 ± 0.03 μm. No flagella were observed for the *Sphingomonas* isolate. Potential presence of polar fimbria (reported in some strains of this genus^53^) would be masked by the large amounts of EPS surrounding the cells in the SEM images. In order to obtain further information about flagella or fimbria, atomic force microscopy (AFM) images were acquired (Fig. 2E–H). Similar sizes for the cells were found compared to SEM results. AFM confirmed the presence of multiple flagella for *Pseudomonas* cells (Fig. 2E) and showed that *Sphingomonas* had fibrous structures protruding from the surface resembling fimbria (Fig. 2H). Presence of fimbria agrees with a study by de Vries et al. that showed an environmental isolate of *Sphingomonas* carrying both fimbria and flagella^35^. Our *Sphingomonas* isolate did not show structures resembling flagella but appeared to mainly have shorter fibers resembling fimbria covering the entire cell surface. AFM images showed *Pararhizobium* cells that were surrounded by a halo of amorphous material gradually thinning toward the periphery, possibly indicating presence of a capsule (Fig. 2F). The maximum height of the halo was 80-100 nm. *Rhizobium* cells were surrounded by extracellular material that was interpreted as EPS (Fig. 2G).

**Fig. 3:**
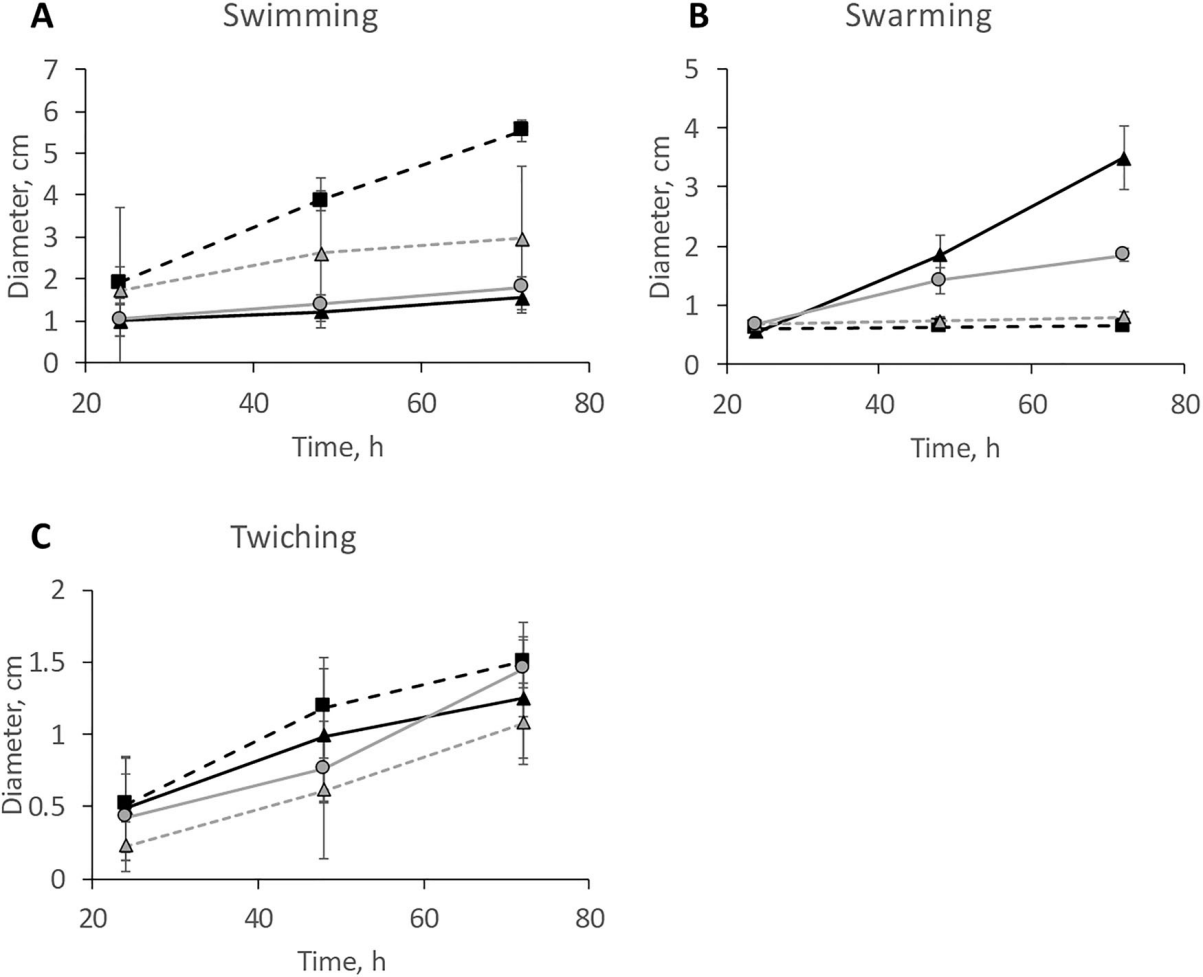
Motility for the four river isolates over a time period of 72 h. The images represent results from motility assays monitoring: **A** swimming, **B** swarming and **C** twitching motility. Symbols represent measurement data. Lines are added to guide the eye. Black triangles linked by a solid black line correspond to *Sphingomonas*, Black squares linked by a dashed black line *Pseudomonas*, Gray circles linked by a gray solid line *Pararhizobium*, and Gray triangles linked with a dashed gray line *Rhizobium*. The data represent an average of 4 plates and the error bars the standard deviation between replicas.

Three types of motilities were investigated for the four isolates; swimming, swarming and twitching. Swimming motility was highest for *Pseudomonas* (Fig. 3A). Swimming motility is well known to be powered by flagella^54^ and the *Pseudomonas* isolate studied here also clearly shows one or several polar flagella in SEM and AFM images^55^ (Fig. 2A, E). Swarming motility was most pronounced in the *Sphingomonas* isolate (Fig. 3B). However, no flagella were observed in SEM or AFM images of this strain (Fig. 2D, H) and flagella have an important function in swarming motility. Thus, the motility observed in the swarming experiments most likely relate to gliding motility, not requiring flagella^54^. It has previously been described that the two types of motilities may easily be mistaken for each other^56^ and *Sphingomonas* species have previously been described to exhibit gliding motility^53^. Thus, we conclude that the movement observed for the *Sphingomonas* isolate was gliding within the large amount of extracellular substances produced by this strain. Also, *Pararhizobium* was motile in the swarming assay and produced large quantities of EPS, possibly also indicating a similar gliding motility. Twitching motility was not pronounced for any of the strains.

### Chemical characterization

**Fig. 4:**
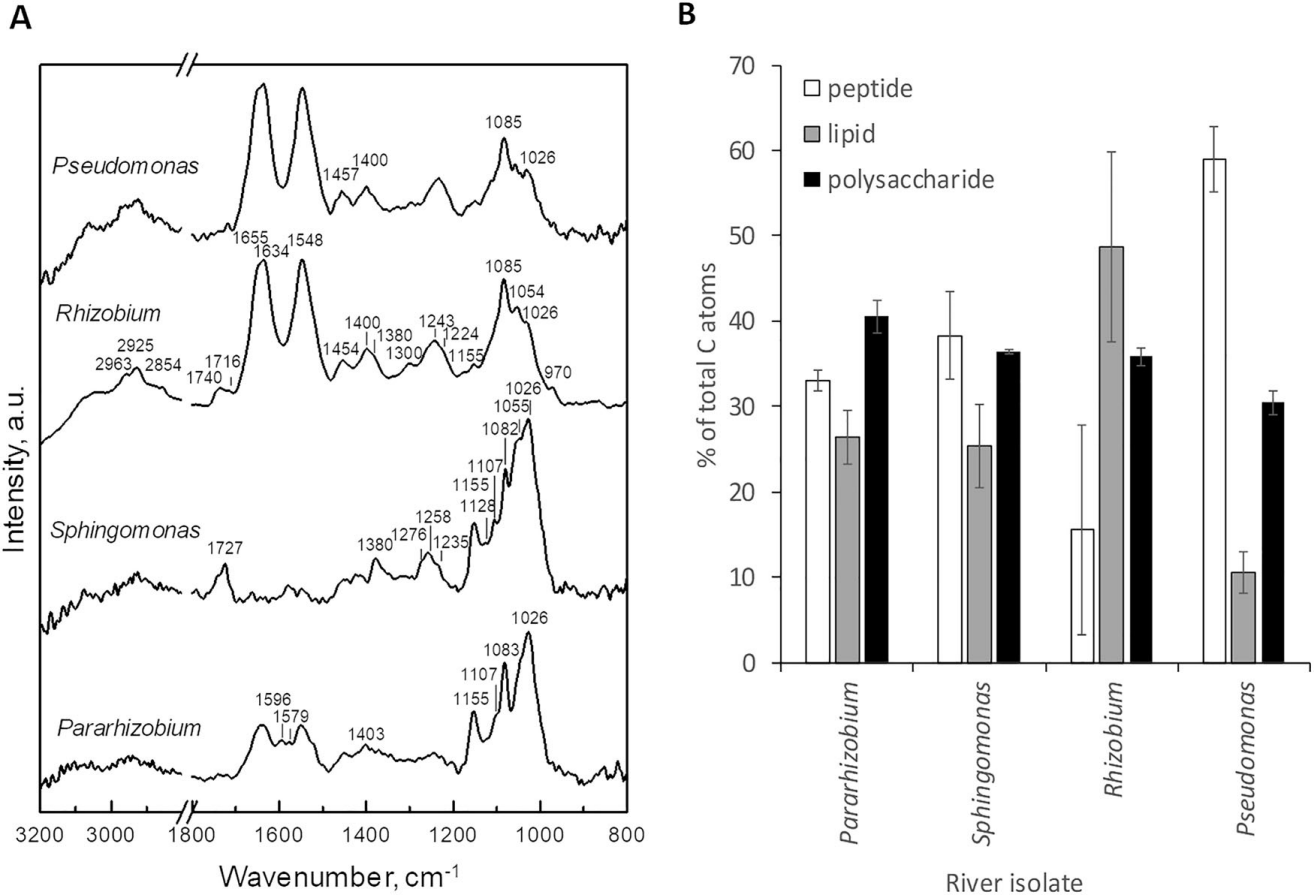
Spectroscopic characterization of bacterial isolates grown on 10% R2A agar. **A** ATR-FTIR spectra showing bands related to biochemical composition and **B** cryo-XPS data for relative content of peptides, lipids and polysaccharides on bacterial surfaces, estimated from the carbon 1 s signal in cryo-XPS analyses of *Pseudomonas, Rhizobium, Pararhizobium* and *Sphingomonas*. Error bars in XPS data represent standard deviation between two biological replicas.

In order to characterize the biomolecular composition of the four isolates, we used three different spectroscopic techniques probing the macromolecular composition of intact hydrated cells. Cells were measured hydrated to reduce the risk of artefacts being introduced due to cell rupture and reorganization of the macromolecular architecture in the sample during drying. Vibrational (attenuated total reflectance Fourier transform infrared, ATR-FTIR, and Raman) spectroscopy, with micrometer-scale (depth/volume) information, enabled monitoring of the composition of entire cells and their surrounding EPS simultaneously. Cryo-X-ray photoelectron spectroscopy (cryo-XPS), on the other hand, probes less than the top 10 nm of a sample surface and therefore exclusively provides information about the cell envelope and any surrounding EPS. Colonies of the four bacterial isolates were collected directly from agar plates and thereafter characterized using ATR-FTIR and cryo-XPS to investigate differences in biochemical composition and surface chemistry (Fig. 4, Supplementary Fig 5).

The ATR-FTIR spectra in Fig. 4A show regions with main contributions from proteins (1500–1700 cm^−1^), nucleic acids (1220–1240 cm^−1^), polysaccharide bands (1000–1200 cm^−1^), and (phospho)lipids (2850–2970 cm^−1^). The relative contribution of these classes of compounds in the bacterial cells varied among species. For *Pseudomonas*, the polysaccharide contribution to the overall fingerprint region (1800–900 cm^−1^) was lower compared to the other three species (proportional intensities: 26% for *Pseudomonas* versus 30%, for *Rhizobium*, 76% for *Sphingomonas*, and 53% for *Pararhizobium*). A slightly lower relative content of polysaccharides was also observed in cryo-XPS spectra of *Pseudomonas* combined with a high protein (peptide) content (Fig. 4B). This corresponds well with the visual appearance of the colonies on the plate, where the *Pseudomonas* colonies appeared to have much less EPS compared to the other three strains. In contrast, a high content of polysaccharides with respect to other components was seen in ATR-FTIR and cryo-XPS spectra of *Pararhizobium* and in ATR-FTIR spectra of *Sphingomonas*. Both these species were characterized with extensive production of extracellular material when growing on agar plates, especially at 10% R2A (Supplementary Fig 3). The spectra of *Pararhizobium* also included broad bands around 1590 cm^−1^ and 1400 cm^−1^ that may be due to asymmetric and symmetric stretching vibrations of carboxylates (COO^–^) present in proteins^57^ (Fig. 4A). For *Rhizobium*, cryo-XPS results showed high relative lipid content compared to other three species (Fig. 4B). In line with this data, ATR-FTIR spectra of *Rhizobium* displayed bands at 2850–2970 cm^−1^, 1380 cm^−1^ and 1300 cm^−1^ assigned to a combination of CH_2_ and CH_3_ molecular vibrations. Furthermore, a band at 1740 cm^−1^ corresponding to C=O stretching vibrations can indicate a contribution of ester groups from lipids/fatty acids (Fig. 4A).

It is interesting to note that ATR-FTIR spectra of *Sphingomonas* from 10% R2A plates did not show amide I and amide II bands from proteins in the region between 1500 and 1700 cm^−1^ as they did for cells from 100% R2A (Supplementary Fig 6). As proteins are abundant in bacterial cells^58^, the lack of corresponding bands in the spectra at lower nutrient content suggested that the material collected from the agar plates for the analysis was predominantly extracellular for this sample. At the same time, bands at 1727 cm^−1^, 1258 cm^−1^, 1276 cm^−1^, and 1380 cm^−1^ were present, characteristic of polyhydroxyalkanoates (PHA, Fig. 4A, Supplementary Fig 7)^59^. These bacterial polymers are typically present in the intracellular compartment and serve as energy-storage compounds. Our ATR-FTIR results from *Sphingomonas* suggested that at least some of these polymers were present in the extracellular matrix of bacterial colonies. It should be noted that the spectrum of *Rhizobium* also showed bands at ~1740 cm^−1^, 1300 cm^−1^, 1054 cm^−1^ and a shoulder at 1380 cm^−1^, which could also be indicative of PHA. However, these bands were less pronounced compared to the spectra of *Sphingomonas* (Fig. 4A). In addition to PHA, the extracellular matrix of *Sphingomonas* contained polysaccharide compounds, as indicated by the intense 1000−1200 cm^−1^ region. Based on previous ATR-FTIR studies of bacterial cells, the bands at 1155, 1082 and 1026 cm^−1^ could be assigned to glycogen, an energy storage polysaccharide produced by various bacteria^60^. These bands were also present in the ATR-FTIR spectra of *Pararhizobium* and partly in *Rhizobium* and *Pseudomonas* (Fig. 4A). It is important to note that the composition of the R2A medium, used to cultivate bacteria, included starch. The molecular structure of starch is similar to glycogen, resulting in a similar set of bands in the same spectral region (Supplementary Fig 8a). However, control experiments of the river strains grown on 10% R2A agar plates without starch showed the same carbohydrate vibrational features (Supplementary Fig 8b). Thus, the bands observed in the spectra are likely to originate mostly from glycogen and/or other polysaccharides produced by the bacteria and not predominantly from the starch of the growth medium.

To complement ATR-FTIR and cryo-XPS measurements, colonies of bacterial isolates were characterized using Raman spectroscopy. The spectra of *Pseudomonas*, *Rhizobium and Pararhizobium* displayed predominantly the bands characteristic of cytochromes due to resonance enhancement with the 532 nm laser^61^ (Supplementary Fig 9). Carotenoids are also selectively and strongly enhanced in Raman spectra using a 532 nm laser. Intense carotenoid bands were observed in *Sphingomonas*, in accordance with the orange color of the colonies (Supplementary Fig 3). While strong resonance effects with cytochromes and carotenoids limit the chemical information obtained by Raman spectroscopy at this laser wavelength, the unique, strong signals of carotenoids provide an excellent route for selectively tracing *Sphingomonas* in co-cultures with the other three isolates (as described later).

Based on the spectroscopic analyses, we concluded that there are differences in chemical composition between the four river isolates. Such differences may result in different types of interactions with the microenvironment surrounding the cells, for example regarding diffusion of pollutants through the colonies. It may also give a base for interactions between species where some isolates may benefit from the presence of the others.

### Biofilm formation by individual isolates

**Fig. 5:**
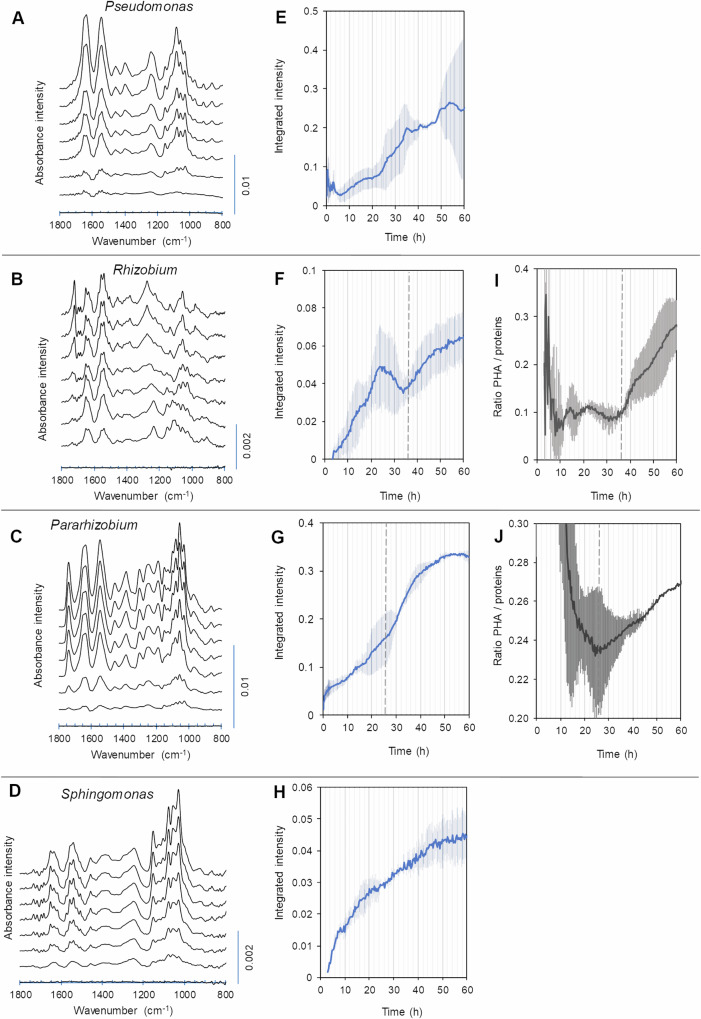
In- situ monitoring of biofilm development by four river isolates. ATR-FTIR spectra of biofilms of **A** *Pseudomonas*, **B** *Rhizobium*, **C** *Pararhizobium* and **D** *Sphingomonas* at (upwards from bottom of each graph) 3, 12, 21, 30, 39, 48, 57, and 66 h after bacterial inoculation. Spectra are offset for clarity. Spectra recorded after 3 h of bacterial inoculation were used as background for spectra presented. **E**–**H** show the averages of integrated intensities in the region between 1271 cm^−1^ and 1192 cm^−1^ from spectra collected every 20 min during two separate experiments. The averages correspond to phosphates present in nucleic acids and phospholipids and are used to illustrate bacterial growth on the ATR crystal for **E** *Pseudomonas*. **F** *Rhizobium* **G** *Pararhizobium* and **H** *Sphingomonas*. **I**–**J** illustrate production of Polyhydroxyalkanoates (PHA) for **I** *Rhizobium* and **J** *Pararhizobium*. PHA was determined by integrating intensities of the characteristic region of the carbonyl band between 1755 and 1705 cm^−1^ for spectra from two separate experiments per isolate. Proteins were determined using integrated intensities of the amide II band (1591–1487 cm^−1^). Error bars represent standard deviation.

Global biofilm formation was first investigated using the classical crystal violet assay using multi-well plates. All four strains showed clear biofilm formation, except *Sphingomonas* that showed weak biofilm formation after 24 h in this assay (Supplementary Fig 10). Thereafter, ATR-FTIR spectroscopy was used to investigate biofilm formation in more detail and in situ (Fig. 5).

In ATR-FTIR, the infrared beam penetrates into the sample to a distance of up to ~2 µm. Therefore, the increase of the intensity of bacterial bands over time indicates settlement of bacterial cells onto the ATR crystal, as well as bacterial growth on the surface of the ATR crystal. Figure 5 shows ATR-FTIR spectra of biofilms of the four isolates recorded every 9 h during 3 days of biofilm growth (Fig. 5A–D). The total spectral intensities (1800–900 cm^−1^) during the whole process of biofilm development (Fig. 5E–H) were lower for *Sphingomonas* and *Rhizobium* compared to the other two strains. This could be due to a generally lower biomass production by *Sphingomonas* and *Rhizobium* during culture growth (Supplementary Fig 2). It is also possible that *Sphingomonas* and *Rhizobium* were forming biofilms as non-surface attached aggregates, which could place them (at least partially) beyond the penetration depth of analysis. Such loose aggregates would also have been easily removed in the washing step of the crystal violet biofilm assay described above.

Spectra from all isolates were characterized with changes observed at the later part of the biofilm formation period, compared to spectra recorded during the first day of biofilm cultivation (Fig. 5A–D). For all species, there was a change in the relative intensities of bands corresponding to C–O and C–OH stretching vibrations in carbohydrates at ~1150 cm^−1^ and ~1030 cm^−1^. These bands decreased with respect to bands in the 1200−1000 cm^−1^ region in biofilms of *Pseudomonas*, *Rhizobium* and *Pararhizobium*, and increased in *Sphingomonas* biofilms as a function of time. High relative content of carbohydrates in *Sphingomonas* biofilms is in accordance with the abundant production of EPS that was observed for this species during cultivation in liquid culture and on agar plates. As with planktonic cells, interference from starch that may have diffused into the biofilm from the medium cannot be ruled out completely. However, as bacteria were shown to produce large quantities of carbohydrates also in absence of starch, we assign these carbohydrate vibrations primarily to a mixture of carbohydrates in the EPS (Supplementary Fig 8).

The most pronounced changes over time occurred in the spectra of *Rhizobium* biofilms (Fig. 5B). In addition to variations in the carbohydrate region described above, there was a remarkable increase in the intensities of bands at 1724 cm^−1^, 1276 cm^−1^, 1057 cm^−1^, and 979 cm^−1^. These bands were previously observed in the FTIR spectra of *Rhizobium* and assigned to C=O, C–O–C, C–O and C–C stretching vibrations of poly(3-hydroxybutyrate), respectively^62^. Poly(3-hydroxybutyrate) is a common PHA synthesized by *Rhizobium* species, and it serves an important role^63–65^. The significant increase in intensities of PHA bands occurred after a period of reduction in bacterial growth at ~36 h of biofilm growth. This is observed in Fig. 5F as a reduction in the integrated intensities of phosphate bands representing bacterial contributions to the spectra. This reduction coincided with an increase in the ratio of integrated intensities of the carbonyl band (from PHA) to the amide II (representing bacterial proteins) (Fig. 5F, I). Considering the static conditions of biofilm growth during these ATR-FTIR measurements, it is possible that the supply of oxygen was limited at the bottom layer of the biofilm, leading to partial detachment or lysis of cells. It has been shown for other *Rhizobium* species that oxygen limitation induces accumulation of large amounts of PHA^63^. Oxygen availability could also influence the kinetics of biofilm formation by *Pseudomonas*. Indeed, the pattern of biofilm formation consisted of periods of faster and slower growth (Fig. 5E), as well as short-term events showing plateauing or drops in fingerprint spectral intensities, similarly to what was observed in the growth curves for this isolate when grown in 96-well plates. It has previously been suggested that oxygen depletion can lead to an oscillating pattern of the kinetics of biofilm formation for *Pseudomonas fluorescens*^66^. Such detachment and reattachment of our *Pseudomonas* isolate is also supported by the high mobility observed in the motility assay for this strain (Fig. 3). Thus, this observation supports the hypothesis that the oscillating pattern, also observed in the automated growth curve, most likely relates to changes in biofilm coverage (as discussed in connection to Fig. 1).

The other two strains—*Pararhizobium* and *Sphingomonas*—showed more “classical” kinetics of biofilm formation (Fig. 5G, H). The growth of *Sphingomonas* biofilms was more pronounced in the beginning of biofilm formation and gradually decreased with time. Interestingly, *Pararhizobium* also contained polyhydroxyalkanoates (PHA), as indicated by bands at 1739 cm^−1^, 1057 cm^−1^, and series of bands between 1330 cm^−1^ and 1160 cm^−1^ (Fig. 5C). The position of the ester carbonyl band and bands between 1330 cm^−1^ and 1160 cm^−1^ was different compared to the bands in the spectra of *Rhizobium* biofilms. Therefore, poly(3-hydroxybutyrate) was not, or at least not the sole, PHA produced by *Pararhizobium* during biofilm formation. As for *Rhizobium*, the significant increase in PHA accumulation was observed relatively long after the start of biofilm formation (~26 h), as demonstrated by the increase of the intensity of the band at 1739 cm^−1^ relatively to the amide II band of proteins (Figs. 5C, G, J). In case of *Pararhizobium*, the production of PHA appeared to increase in connection to the onset of more rapid kinetics of biofilm growth.

To conclude, the in situ FTIR analyses showed that all four isolates form biofilms, but that the dynamics of biofilm formation as well as the content and organization of EPS varied between them. All biofilms contained carbohydrates, but these appeared to be consumed or replaced over time to a larger extent in the *Rhizobium* isolate than in the others. *Sphingomonas* biofilms, on the other hand, appeared to increase in carbohydrate content over time. Interestingly, the presence of PHA in biofilms differed to some extent to what was observed for bacterial cultures on agar plates. In the cells from agar plates, PHA was observed for *Sphingomonas* and in *Rhizobium*, but in biofilms PHA production was most pronounced in *Rhizobium* and *Pararhizobium*. Spatial heterogeneities may play a role here, as the application of biomass to the crystal surface differs between the two experiments. Here, the biofilm grew directly on the ATR crystal, enabling in situ measurements of intact biofilms, whereas measurements of biomass from agar required collection and application of biomass onto the crystal surface.

### Effects of stressors on individual isolates in planktonic form

**Fig. 6:**
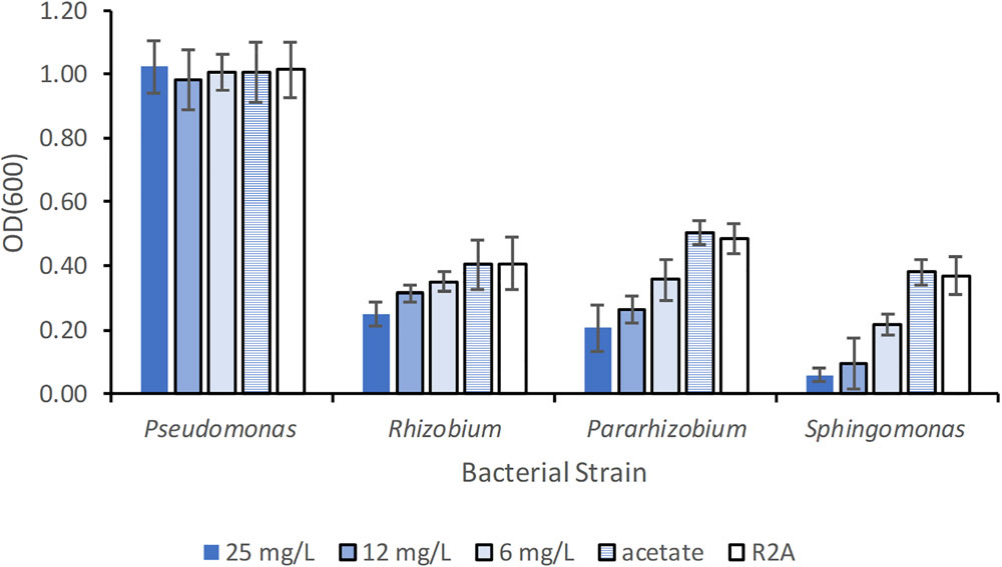
Average growth of planktonic bacteria after 24 h incubation in absence and presence of trimethoprim at pH 7. Error bars represent the standard deviation for growth in 12–27 wells from at least two biological replicas. No significant difference was observed for the growth of *Pseudomonas* at pH 7. The growths of *Sphingomonas* and *Pararhizobium* were significantly reduced compared to control at all concentrations (*p* « 0.01) at pH 7, and the growth of *Rhizobium* was significantly different from control at 12 mg/L and 25 mg/L (*p* « 0.01, students *t*-test) at pH 7. The “acetate” control had a pH of 7 and served as a control for the addition of trimethoprim solution into R2A. R2A was a control with only growth medium.

Stressors may affect both bacterial cells and EPS in a biofilm. To study the effect on cells only, we investigated their culture density in planktonic cultures in liquid broth after 24 h growth. The concentration was chosen to be in the range of EC50 values reported for bacterial cells with respect to trimethoprim^35^, in order to study both possible effects of killing as well as adaptations under stress conditions. All individual isolates except *Pseudomonas* were sensitive to the presence of trimethoprim in a concentration-dependent manner (Fig. 6 and Supplementary Figs 11 and 12), giving reduced culture densities. The most sensitive strain was *Sphingomonas*, displaying the largest reduction in growth in the presence of 25 mg/L trimethoprim with 16% of growth compared to R2A control after 24 h at pH 7. *Pararhizobium* was the second most sensitive at 25 mg/L with 42% growth, and *Rhizobium* displayed 61% of growth in R2A control after 24 h. Overall growth (as measured by optical density after 24 h) of the four strains was not significantly different between pH 7 and pH 5 (Supplementary Fig 12), except at the highest concentration 25 mg/L (*p* < 0.01). Thus, light scattering of cells in liquid culture indicated that although the uptake of trimethoprim may differ between pH 7 and pH 5, cells were also affected when exposed to the protonated form of the drug.

**Fig. 7:**
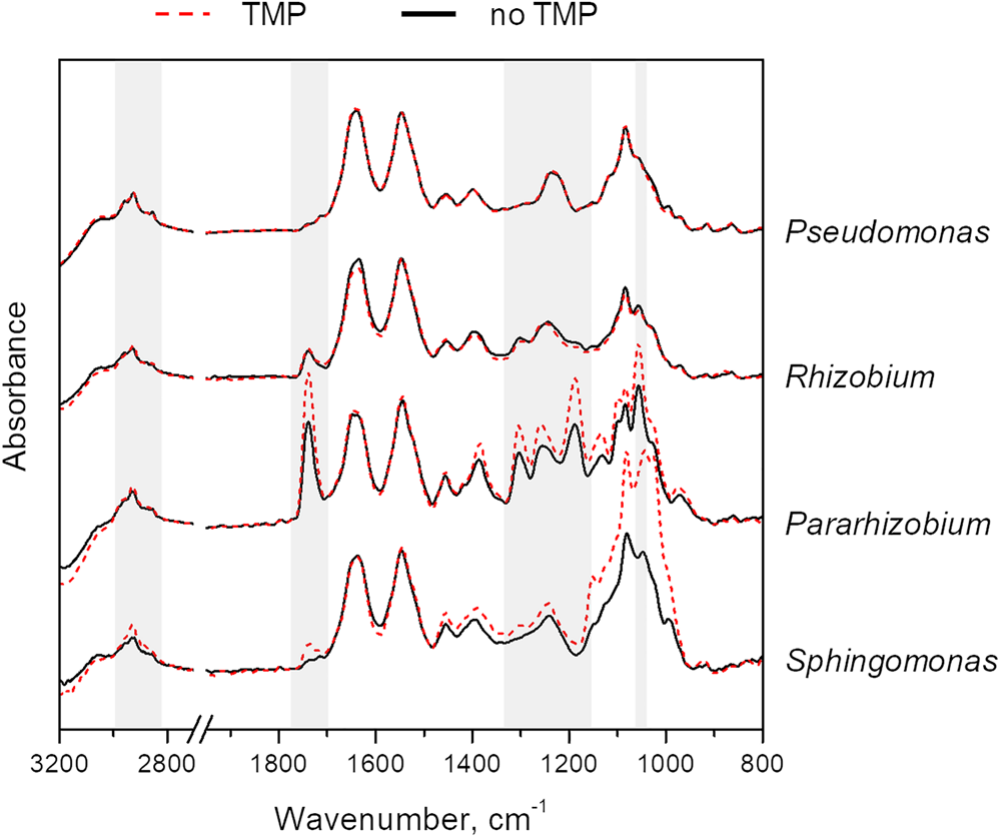
ATR-FTIR spectra of bacterial cells after 24-h growth in planktonic cultures with (red dashed line, 25 mg/L) or without trimethoprim (black solid line) at pH 7. Specific PHA bands are marked to highlight changes between the two conditions, clearly visible in *Pararhizobium* and *Sphingomonas* strains. For comparison, spectra are normalized to the amide II band at ~1540 cm^−1^.

ATR-FTIR measurements were performed to study biochemical alternations in planktonic cells following incubation with 25 mg/L trimethoprim. Spectra from *Pseudomonas* and *Rhizobium* remained unchanged, but *Pararhizobium* and *Sphingomonas* altered their biochemical composition after exposure to trimethoprim indicating formation of PHA^59^. This can be seen in Fig. 7 as an increase in intensity of the bands at ≈1740 cm^−1^ and 1057 cm^−1^, corresponding to C=O and C–O stretching vibrations, respectively. The bands in the regions 2830–2970 cm^−1^ (CH_2_, CH_3_ stretching vibrations) and 1165−1320 cm^−1^ (C–O–C stretching vibrations) also increased (Fig. 7). As described above (sections “Chemical characterization” and “Biofilm formation by individual isolates”), pure cultures of *Rhizobium* and *Sphingomonas* produced PHA after 3 days when grown on culture plates. On solid surfaces, PHA was also observed to form as part of the biofilm growth cycle for *Rhizobium* and *Pararhizobium*, at time periods longer than ca 30 h. However, in presence of trimethoprim, a change in PHA production was observed already at 24 h, indicating a change in biomolecular composition promoted by the presence of trimethoprim.

PHAs such as polyhydroxybutyrate are known to be synthesized inside a wide variety of bacterial species and biofilms^67^. Multi-species consortia residing in environments exposed to stressors have been described to frequently include organisms that are able to synthesize PHA^25^. These substances have been reported to provide an increased fitness and survival for bacteria during exposure to many types of stressors, for example low or high temperatures, desiccation, UV-radiation, solvent, osmotic shock, toxic substances and oxidative conditions^25,68,69^. Thus, an increased synthesis of PHA in bacteria and biofilms exposed to trimethoprim indicated a stress response that most likely protected both *Sphingomonas* and *Pararhizobium*, and may also benefit other bacteria when these isolates reside in multi-species biofilms. The *Rhizobium* isolate did not respond to trimethoprim by increased production of PHA despite the ability of this strain to do so. This may be related to the observed differences in sensitivity to trimethoprim (Fig. 6). *Sphingomonas* and *Pararhizobium* were the two most sensitive and consequently may have been more stressed by the presence of trimethoprim than *Rhizobium*.

### Biofilm morphology and architecture

**Fig. 8:**
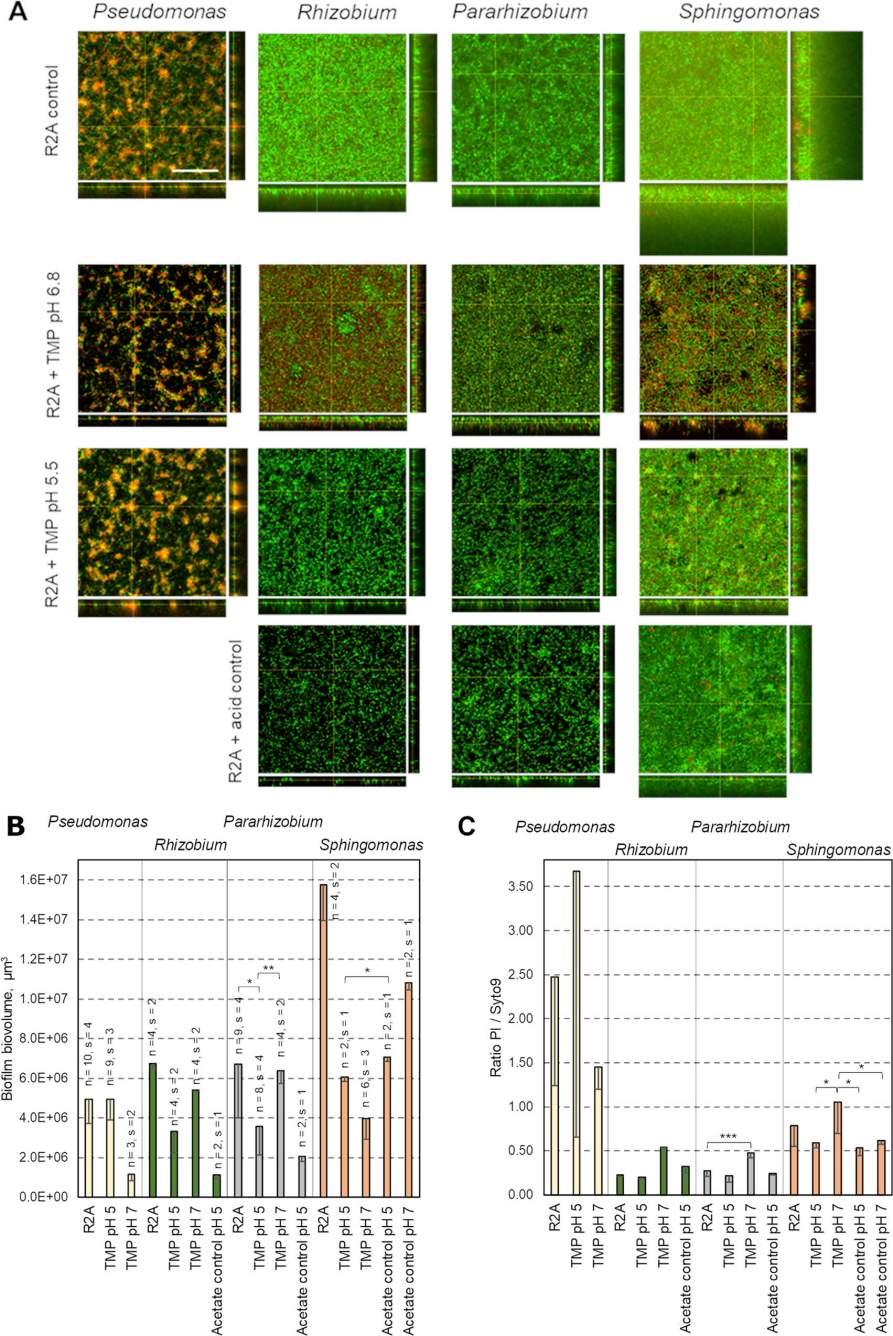
Biofilm morphology and architecture of mono-species cultures. **A** Representative CLSM images of biofilms exposed to trimethoprim at neutral and acidic conditions with corresponding controls. Scale bars represent 100 μm. The same magnification was used for all images. The images represent smaller parts of the full images given in Supplementary Fig 14. Quantification of parameters from CLSM images using BiofilmQ for **B** biofilm volume and **C** staining ratio red/green (PI/Syto9) as a measure of the proportion of cells with damaged membranes. (n) is a total number of images from (s) number of samples. *, **, and *** indicate *p* ≤ 0.05, *p* ≤ 0.01, and *p* ≤ 0.001 (*t*-test) in selected samples.

To study the combined effect of trimethoprim on both cells and EPS confocal laser scanning microscopy (CLSM) was used. In the unexposed biofilms, confocal microscopy analyses revealed species-specific differences in the structure and architecture (Fig. 8). *Pseudomonas* formed a biofilm consisting of islands growing several µm apart from one another and with a height of up to ~40 µm from the substrate surface. The cells present in colonies were predominantly stained red with *Bac*Light^TM^ dyes. Therefore, the membrane in *Pseudomonas* biofilm cells was compromised in some way, or the bacteria at the surface were dead. A third explanation for the red color could be that the biofilm contained extracellular DNA that was stained. An abundance of “dead” cells would be contrary to the ATR-FTIR fingerprints obtained of *Pseudomonas* biofilms (Fig. 5A). In a study on *Pseudomonas fluorescens*^46^, it was shown that bacteria in the death phase have low intensity of bands corresponding to nucleic acids (1220–1240 cm^−1^, 1085 cm^−1^, 915 cm^−1^). In our study, the symmetric stretching of phosphate moieties at 1085 cm^−1^ was covered by a carbohydrate band at 1082 cm^−1^. Nonetheless, high relative intensities of bands assigned to the asymmetric stretch of phosphate groups at 1220−1240 cm^−1^ and ribose-phosphate motions at 915 cm^−1^ after 3 days of growth suggest that cells were growing in the vicinity of the surface or encapsulated by extracellular DNA. Optical microscopy observations showed that in addition to colonies, a high number of cells in *Pseudomonas* biofilms remained unattached to the surface and these cells were highly motile. The microscopy analyses showed that these motile cells were all stained green and moved rapidly around the colonies. This may suggest that the biochemical signatures obtained by ATR-FTIR measurements could have originated from motile cells close to the ATR crystal in *Pseudomonas* biofilms. However, considering the high motility, it is not likely that these cells would be close to the crystal for a sufficiently long time to, by themselves, give rise to the FTIR fingerprint observed. Furthermore, staining of the stationary biofilms using CTC (5-cyano-2,3-ditolyl tetrazolium chloride) with DAPI (4’-6 diamino-2 phenylindole) as counterstain indicated that biofilm cells were metabolically active both in presence and absence of trimethoprim (Supplementary Fig 13). Thus, put together, these observations suggest that the cells at the surface of the crystal were alive and either had a high membrane potential, a “leaky” membrane^70^ or were encapsulated with extracellular DNA. The presence of extracellular DNA has been well documented for *Pseudomonas aeruginosa*^71–74^ and described to be a recurring theme in biofilms from many types of bacteria^75^. Its presence in this river isolate is still an open question. If present, it may assist in adhesion to the substrate surface^76^.

Compared to the biofilms of *Pseudomonas*, biofilms of the other three isolates were characterized by a more homogeneous coverage of the surface (Fig. 8). *Rhizobium* and *Pararhizobium* formed biofilms that were up to ~60 µm thick. Biofilms of *Pararhizobium* formed complex three-dimensional patterns that could be observed both visually and in optical microscopy. *Sphingomonas* formed biofilms with a remarkable thickness of ~150 µm. Clusters of cells, stained red, and voids were sporadically observed at the bottom of *Sphingomonas* biofilms. Previous studies have described that partial cell lysis can be beneficial for the remaining subpopulation of viable cells in thick biofilms^77^. Furthermore, void formation and growth of biofilms several micrometers away from the surface was reported in a study of *Sphingomonas* biofilms under flow conditions^78^. This phenomenon would explain the low intensities of the ATR-FTIR spectral bands observed in our study for *Sphingomonas* biofilms. Such a biofilm could also be expected to have lower adhesion to a surface, explaining the very large difference in apparent biofilm formation between the crystal violet assay and the in-situ optical microscopy shown in Fig. 8. However, the difference in biomass between 24 h and 72 h may also reflect the slower growth of *Sphingomonas* resulting in a delayed biofilm formation (Fig. 5 and Supplementary Fig 10).

**Fig. 9:**
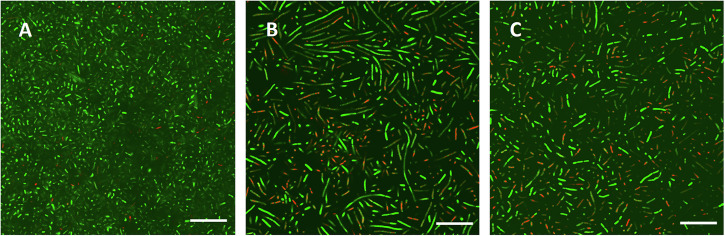
*Sphingomonas* cell morphology in presence of trimethoprim. High resolution images of *Sphingomonas* cells at **A** pH 7 (with acetate), **B** pH 7 and trimethoprim (with acetate), **C** pH 5 and trimethoprim (with acetic acid). Scale bars represent 20 µm in all images.

As a next step, biofilm morphology was monitored at pH 7 and pH 5 after exposure to trimethoprim (Fig. 8). In the images of three of the isolates, changes in ratio between cells stained red or green (from live—dead staining) could be observed, as well as changes in biofilm volume (Fig. 8). However, *Pseudomonas* cells did not appear to change in color as they were already predominantly red in biofilms also under control conditions. This red color, thus, made it difficult to visually estimate the impact of trimethoprim on *Pseudomonas* cells in the biofilms (Fig. 8C). No alterations were observed in the maximum thickness of *Pseudomonas* biofilms between conditions (29 ± 7 μm in R2A, 23 ± 7 μm at pH 7 and trimethoprim and 27 ± 7 μm at pH 5 and trimethoprim) and the biofilm volume remained similar between control and exposed at pH 5. However, cells exposed at pH 7 showed a lower biofilm volume despite having similar morphology to the non-exposed cells (Fig. 8B). Nonetheless, the similarity in morphology between conditions as well as the observed growth of planktonic cultures in the presence of the antibiotic (Fig. 6) suggest that *Pseudomonas* cells were not susceptible to trimethoprim at these concentrations, but possibly the presence of trimethoprim promoted cells to detach from the surface giving a reduced overall biofilm volume at pH 7. Similar to planktonic cells (Figs. 6, 7), the most dramatic influence of trimethoprim on biofilms occurred in *Sphingomonas* samples. Fluorescent staining of cells with *Bac*Light^TM^ kit revealed a higher number of damaged cells in conditions with trimethoprim at pH 7 than in conditions with trimethoprim at pH 5 (Fig. 8). At pH 7, the number of damaged cells was also higher than in conditions with acetate/acetic acid alone, suggesting that the damaging impact was associated with trimethoprim. In the R2A control, the number of damaged cells in *Sphingomonas* biofilms was not significantly different from the conditions with trimethoprim at pH 7. The presence of red-stained cells in this case could be a result of remarkably thick biofilms and cell damage at the bottom layers. Indeed, the volume of biofilm in control conditions of R2A medium alone was 1.5 times higher than in the conditions with acetate at pH 7 and 2.2 at pH 5. This ratio further increased to 4.0 in conditions with trimethoprim at pH 7 and 2.6 at pH 5 (Fig. 8). The effect observed in the acetate/acetic acid control indicated an effect of pH or acetate on EPS accumulation in biofilms, since planktonic cells did not show a decreased cell density in the acetate/acetic acid control (Fig. 6 and Supplementary Fig 12). Possibly, reduced EPS content may have enhanced cell dispersion and thus increased fitness of cells remaining in the biofilm by facilitating access to nutrients and removal of waste metabolites from the biofilm. Furthermore, CLSM results showed that *Sphingomonas* cells were elongated when exposed to trimethoprim and intermittently stained in red and green (Fig. 9). Thus, cell density results (Fig. 6), fluorescent staining, biofilm volume data, and morphological analysis of *Sphingomonas* cells in biofilms all suggests that trimethoprim had a negative impact at both pH 5 and pH 7, albeit with more pronounced effect at neutral conditions.

*Pararhizobium*, the strain that was the second most sensitive to trimethoprim (Figs. 6, 7), also displayed altered biofilm morphology. The overall quantification based on live/dead staining suggested pronounced membrane damage at pH 7 but this difference was not detected at pH 5. Despite the sensitivity of *Pararhizobium* to trimethoprim in planktonic cultures, biofilm volumes with and without trimethoprim stayed similar at pH 7. This suggests that the capacity of *Pararhizobium* to produce extracellular matrix remained unaffected by trimethoprim at neutral conditions despite the negative effect observed on planktonic and biofilm cells. This could potentially be due to the EPS providing protection or increased adhesion for the cells in the biofilm. The biofilm volume decreased in both exposed and non-exposed conditions at pH 5, indicating changes in EPS production induced by the lower pH (Fig. 8). The fourth isolate, *Rhizobium*, showed quantitative results very similar to *Pararhizobium*. A clear alteration in ratio between red/green in the live/dead staining indicated membrane damage in the presence of trimethoprim at pH 7 (Fig. 8). However, in contrast to *Pararhizobium*, the biofilm volume was reduced in the presence of trimethoprim already at pH 7 (Fig. 8). It was further reduced in the two conditions at pH 5, in line with the optical density data that showed larger effect by trimethoprim on cell density at pH 5 (Fig. 6 and Supplementary Fig 12). However, the live/dead staining seem to contradict these two more quantitative measures on optical density of planktonic cells and biofilm volume. The reasons for this remain elusive. One possibility is that dead *Rhizobium* cells detached more easily form the biofilm at pH 5 due to the reduced quantity of EPS, thereby reducing the overall red staining as the biofilm volume shrunk.

Bacteria have been suggested to exhibit lower uptake of trimethoprim at pH 5 compared to pH 7^33^. Our results are generally in line with this hypothesis, although an effect of trimethoprim was also observed at pH 5. In addition to this effect, our results indicate an impact of acidity on the production and accumulation of extracellular matrix in the biofilms. Therefore, at acidic conditions, biofilm cells were influenced by a combination of two mechanistically different stressors simultaneously. These multiple-stress conditions were therefore studied more closely with respect to responses in a co-culture biofilm model system.

### Co-cultivation

The four bacterial isolates used here were collected from the same substrate in the small river. Thus, we can assume that in their natural environment, these four isolates lived in some level of proximity of each other (and other river bacteria). A simple cross-cultivation assay showed that all strains could co-exist without visible antagonism in the culture conditions used (Supplementary Fig 15). As a next step, biofilm architecture was investigated also for biofilms from the four species consortium using confocal microscopy.

**Fig. 10:**
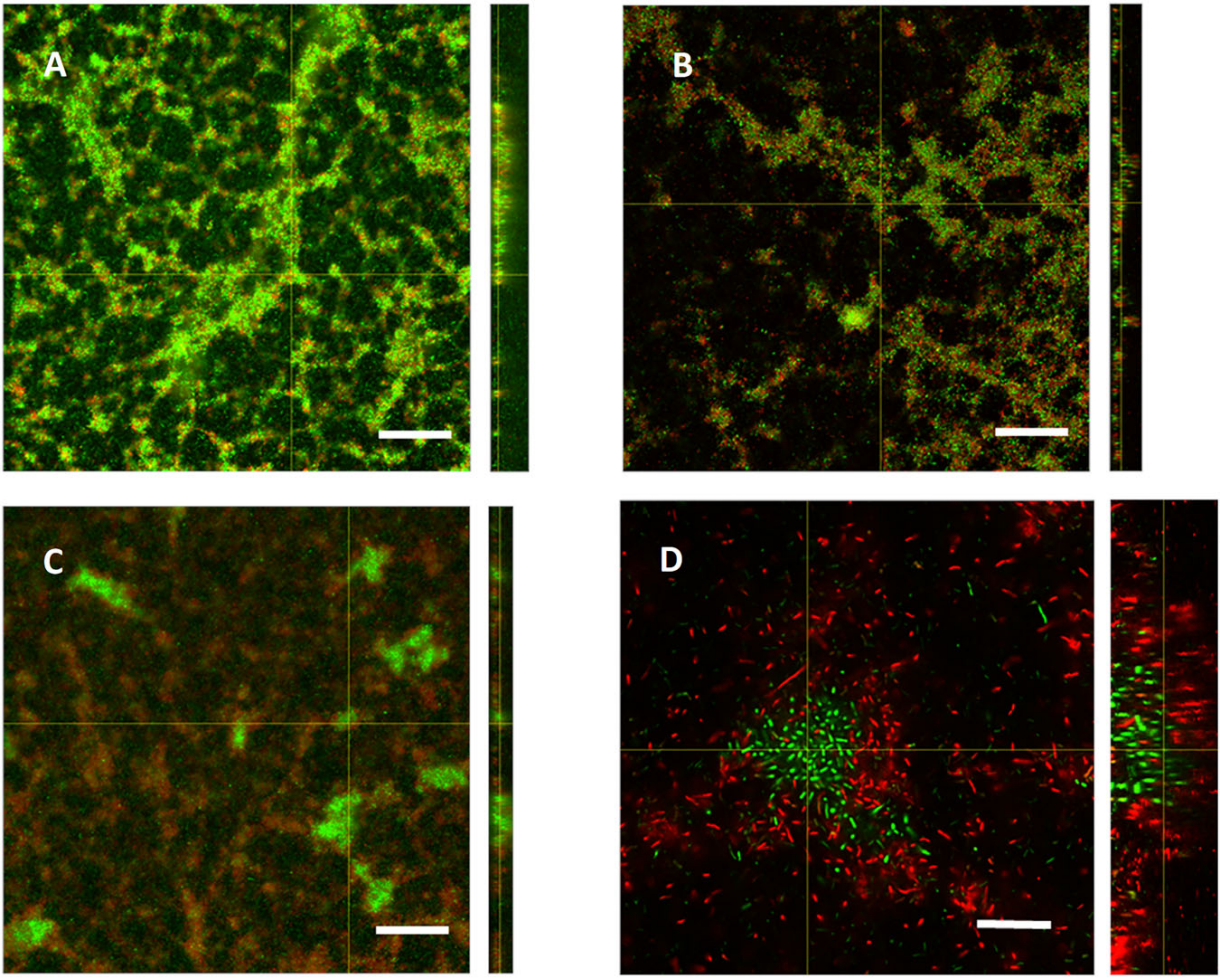
Biofilm morphology and architecture of four-species consortia. CLSM images of four-species biofilms **A** unexposed (representative from 8 images on 3 samples), **B** exposed to trimethoprim at pH 7 (representative from 2 images on 2 samples), **C** exposed to 25 mg/L trimethoprim in acidic conditions showing clustering green cells surrounded by red cells (BacLight^TM^ staining) (representative from 7 images on 3 samples). **D** Higher magnification of (**C**). The apparent blur of cells around the colonies is due to the high motility of the *Pseudomonas* cells, in accordance with control observations of single-species biofilms. Scale bar in **A**–**C** represents 100 µm and in **D** 20 µm).

The biofilms formed by the four-species consortium had features resembling the structure of *Pseudomonas* single-species biofilm at the bottom, but with matrix-associated cells at the top layer (Figs. 8, 10). The architecture of the matrix at the top layer of the biofilm was similar to the matrix of *Pararhizobium* mono-species biofilms, or dual-species biofilm with *Pseudomonas* and *Pararhizobium* (Supplementary Fig 16). This suggests a layered organization inside the multi-species biofilm where *Pseudomonas* presumably dominated at the bottom layers. The chemical fingerprint of the top layers of such a biofilm was not possible to monitor in situ using ATR-FTIR spectroscopy, due to their distance from the ATR crystal surface. However, based on the data from single-species biofilms, the visual similarity to the *Pararhizobium* biofilms and the FTIR data, we hypothesize that the top matrix may have originated from *Pararhizobium* and contained PHA (Fig. 5C). The thickness of the four-species biofilm was around 60 µm. Hence, the presence of other species restricted *Sphingomonas* cells from forming exceedingly thick biofilms (Figs. 8, 10).

The four-species biofilm grown with trimethoprim at pH 5 had visually no matrix-associated layer of cells at the top of the biofilm, as was observed for the unperturbed system. Instead, we observed a biofilm structure reminiscent of *Pseudomonas* mono-species biofilms with cells predominantly stained red, dotted by islands of incorporated cells that were stained green (Fig. 10C, D). The visual similarity of the co-culture biofilm to the *Pseudomonas* biofilm suggested a dominance of *Pseudomonas* in the consortium, with the other cells forming the clusters. In order to investigate these interactions further, strains were grown in combinations of two (*Pseudomonas* + *Pararhizobium*), three (*Pseudomonas* + *Pararhizobium* + *Rhizobium*) and four strains (*Pseudomonas* + *Pararhizobium* + *Rhizobium* + *Sphingomonas*) and the morphology investigated using confocal microscopy. Formation of small islands, as observed in the four-species biofilm, were observed only when *Sphingomonas* was present in the biofilms. Thus, we hypothesized that the green cells in Fig. 10C, D corresponded to intact, green *Sphingomonas* cells forming clusters inside a biofilm dominated by the more tolerant *Pseudomonas* strain that was stained red due to extracellular DNA.

**Fig. 11:**
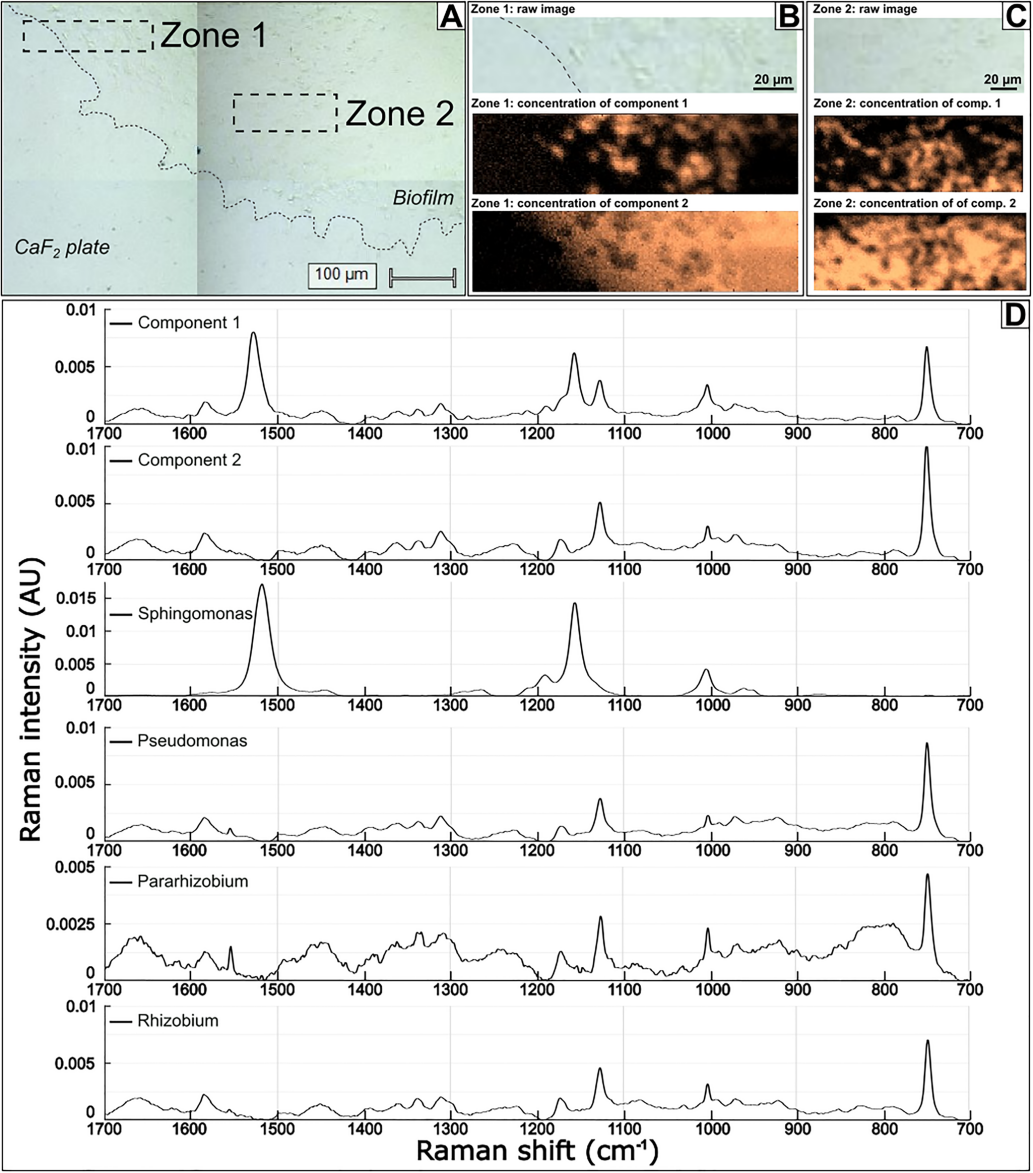
Raman microspectroscopy of the four-species consortia on a CaF_2_ substrate. The image shows **A** two zones from within the biomass **B**, **C** the two zones enlarged as bright field images and as maps of multivariate curve resolution alternating least squares (MCR-ALS) resolved spectral Components 1 and 2. **D** Representative Raman spectra corresponding to areas with high abundance of Component 1 and Component 2 as well as Raman spectra of individual river isolates of the consortium (as in Supplementary Fig 9). Component 1 shows high levels of carotenoids from *Sphingomonas* cells, and low levels of cytochromes from the other bacterial isolates. Component 2 mainly shows cytochromes similar to the individual spectra of *Pseudomonas, Pararhizobium* and *Rhizobium*. Spectra in **D** show actual Raman spectra corresponding to regions with high intensity of each of the two components of main interest. The mathematically resolved components are instead shown in Supplementary Fig 17.

To investigate the organization inside the four-species co-culture further, we used confocal Raman microspectroscopy (Fig. 11) and multivariate analysis. In spectra and hyperspectral maps of the co-culture, chemical heterogeneities were clearly observed and were assigned to molecular features shown in Table 1.

**Table 1:** Raman shifts with assignments. For more details regarding assignments of the exact vibrations, please consult references indicated

Raman shift (cm^−1^)	Assignment	Potential origin, Ref
1660	Amide I	Proteins^79^^,^^83^,
1580	Methine bridge, aromatic C=C	Cytochrome (heme)^83^,
1528	C=C	Carotenoids^94^,
1460–1450	-C-H	Unspecific, lipids^79^
1390	-COO	Unspecific ^79^,
1360	-CH, incl. aromatic	Cytochrome (weak), unspecific^83^
1330	-C-H	Unspecific ^83^,
1310	-C-H, incl. aromatic	Cytochrome ^83^,
1225	Amide III, -C-O	Proteins^79^^,^^83^,
1172	C-C, -C-O-C-	Unspecific, polysaccharides^79^
1155	=C-C=	Carotenoids^94^,
1130	C-N	Cytochrome (heme)^83^,
1003	-C-C-, incl. aromatic	Phenyl alanine, lipids, proteins^79^,
780	Ring breathing.	Nucleobases (pyrimidine)—C, U^79^,
750	pyrrol ring breathing	Cytochrome (heme)^83^,
720	Ring breathing	Nucleobases (purine)—A^79^,

Spectra from individual strains and the co-culture (Figs. 11, Supplementary Fig. 9) showed Raman Resonance (RR) effects with the 532 nm laser. *Sphingomonas* showed characteristic RR bands assigned to carotenoids at 1155 cm^−1^ (=C‑C=) and 1528 cm^−1^ (−C=C-)^79^. The Raman spectra of the three other strains showed RR bands assigned as cytochrome: at 750 cm^−1^ (ring vibrations), 1130 cm^−1^ (C-N), and 1580 cm^−1^ (aromatic ring stretching), which can be assigned to fingerprints of the heme protein. These features were also observed in the spectra from co-culture. Amide I and III bands from proteins were observed at ca. 1660 cm^−1^ and 1225 cm^−1^, respectively. Both bands were broad, indicating a variety of secondary structures. Other, considerably less intense bands could be assigned to specific amino acids, but without further detailed analyses such assignments remain tentative and are not listed here, except for the characteristic sharp band of phenyl-alanine at 1003 cm^−1^.

The unique features assigned to carotenoids in *Sphingomon*as cells enabled investigations of the spatial distribution of these bacteria in the co-cultures. Figure 11B, C shows the result from multivariate analysis of hyperspectral images using multivariate curve resolution alternating least squares (MCR-ALS). The distribution maps show that both analyzed zones contained clusters of cells with carotenoids surrounded by cells with cytochromes. Inside the clusters, cytochromes could be observed indicating that although *Sphingomonas* cells formed dense clusters, these were not mono-species colonies but contained cells from other species. Most of the variation in the multispectral maps could be explained by the two first components of the multivariate analysis (Supplementary Fig 17). For Zone 1, Component 1 represented mainly carotenoids, Component 2 from the analysis corresponded to the CaF_2_ plate, Component 3 cytochromes, Component 4 spectral noise and the fifth component contained the same chemical information as Component 3, with varying intensities between cytochrome signals. For Zone 2, Component 1 again was represented by mainly carotenoids, Component 2 by cytochromes, Component 3 contained spectral noise and Component 4 contained the same chemical information as Component 2, with varying intensities between carotenoids and cytochrome signals. Thus, Component 4 in Zone 2 most likely depicted variations in the ratios of different bacteria within the measurement volume.

**Fig. 12:**
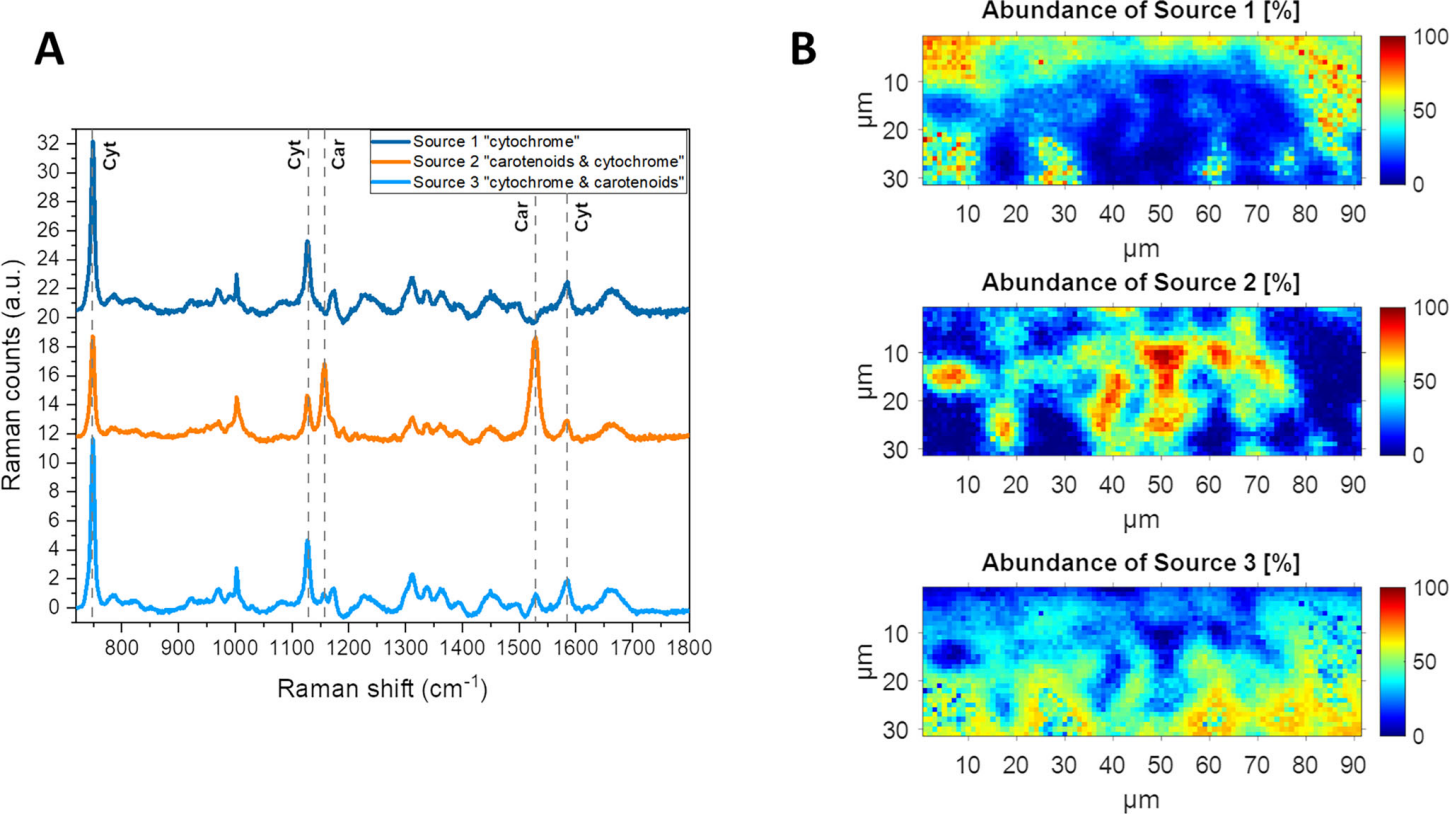
BPSS analysis of Zone 2 from the Raman microspectroscopy data for the four-species consortia. **A** Estimated sources from BPSS calculation (spectra are offset for clarity) and **B** their corresponding maps of abundances. The three sources could explain 98% of the data variance. Cyt cytochrome, Car carotenoids.

In addition to MCR-ALS, we also performed Bayesian positive source separation (BPSS) analysis of the hyperspectral data from Zone 2 of the unperturbed system (Fig. 12). The multivariate contributions, which in this case are called sources instead of components, corresponded to the RR effects described above. Source 1 showed only the cytochrome signature, Source 2 was a mixture of mainly carotenoids with a contribution of cytochrome and Source 3 was a mixture of mainly cytochrome with a contribution of carotenoids, indicating a gradient in species distribution, similar to what we observed in the MCR-ALS analysis. Thus, both probabilistic and iterative least-squares fitting analyses converge on similar solutions. Even though the resolved profiles are not chemically pure, they reveal a spatial organization where *Sphingomonas* cells, containing carotenoids, form small clusters of cells in the co-culture^80,81^, surrounded by bacteria containing cytochromes in different ratios throughout the biomass (Figs. 11, 12).

Clustering of one bacterial species inside the biofilm of another has previously been reported in the literature. For example, Limoli et al. showed that *Pseudomonas* cells can display a special type of motility in the presence of other bacteria, called exploratory motility^82^. This feature was described to influence the spatial distribution of other cells and usher them into dense colonies surrounded by *Pseudomonas* cells. The resolved Raman maps suggest that this happens in our co-cultures as well.

**Fig. 13:**
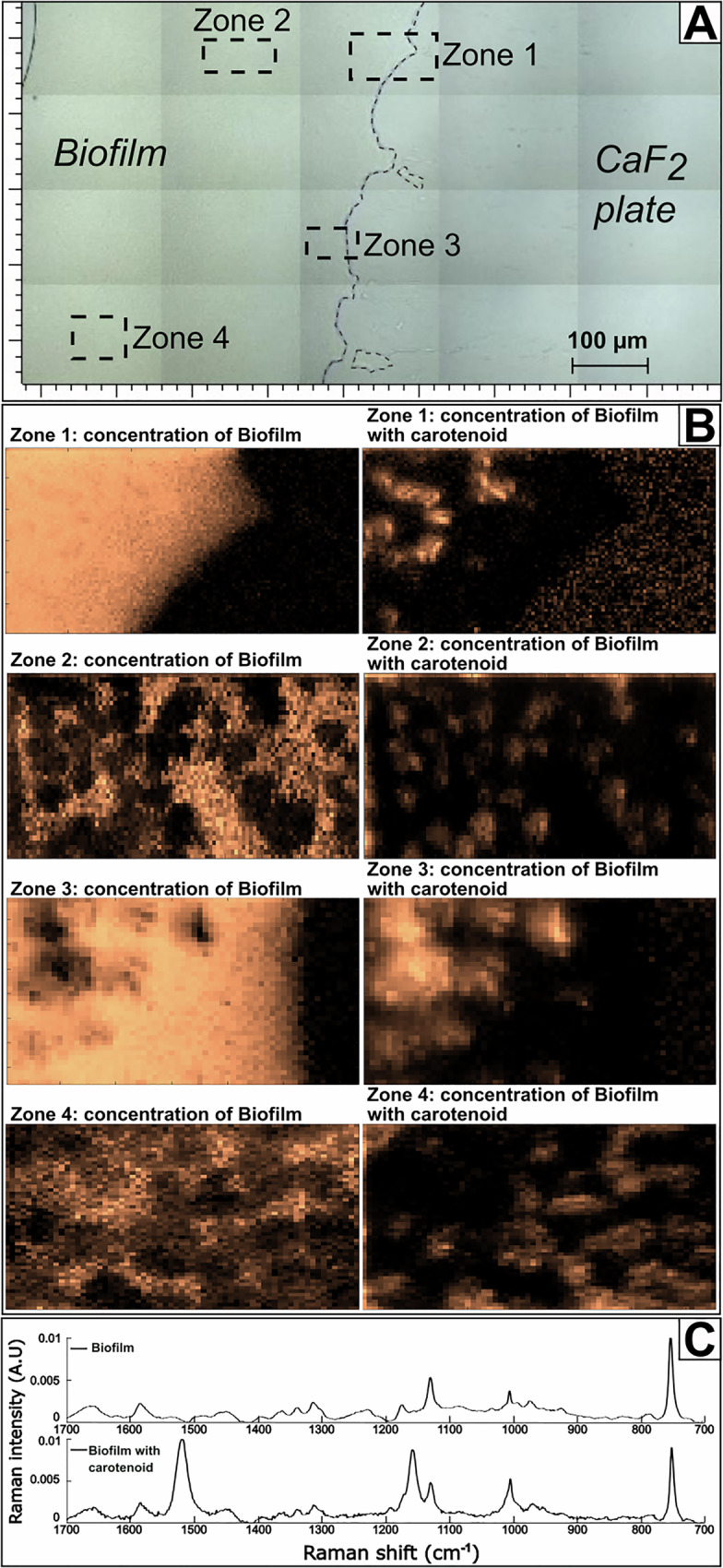
Raman microspectroscopy of co-cultures exposed to 25 mg/L trimethoprim in acidic conditions. The image shows **A** optic microscopy image showing four zones from within the biomass of the sample, **B** corresponding maps of distribution of spectral components with cytochromes and carotenoids within the four zones. **C** Representative Raman spectra corresponding to areas with high abundance of cytochromes and areas with high abundance of carotenoids (i.e., *Sphingomonas*), but with additional presence of cytochromes (i.e., *Pseudomonas, Rhizobium* and/or *Pararhizobium*).

Raman analyses of the four-species consortium exposed to trimethoprim at pH 5 were not dramatically different from the non-exposed system (Fig. 13, Supplementary Fig 18). *Sphingomonas* cells with carotenoids formed small clusters inside the multi-species consortium. The biomass surrounding these clusters showed bands characteristic of cytochromes (Fig. 13)^83^. Cells with cytochromes were present to a lower level inside the *Sphingomonas* clusters indicating that the clusters did not consist exclusively of *Sphingomonas* cells. Although we cannot rule out the presence of other species in the co-culture biofilm, the sensitivity of *Rhizobium* and *Pararhizobium* to acidity observed in the planktonic assay and in individual species biofilms suggest they may have a lower presence in the co-culture biofilm at pH 5. Furthermore, symbiotic interaction of *Pseudomonas* and *Sphingomonas* spp. have previously been reported during degradation of chlorinated compounds^84^. This suggest close interactions between *Sphingomonas* and *Pseudomonas* cells in the co-culture. Such close interactions would enhance metabolic exchanges and can indicate that these bacteria benefit from co-localization. The microcolonies of *Sphingomonas* cells inside the four-species biofilm did not appear to show the cell elongation that was observed in the trimethoprim exposed monoculture at pH 5 (Figs. 9, 10). This suggests benefits of co-culture living for *Sphingomonas* also under stress conditions caused by high concentrations of the antibiotic trimethoprim.

In conclusion, the combined confocal microscopy and Raman microspectroscopy suggest that heterogeneities in the four species consortium combined with the presence of the more resistant *Pseudomonas* isolate, in co-culture, may have protected the more sensitive *Sphingomonas* during stress conditions induced by trimethoprim. Thus, *Sphingomonas* appeared to have received a fitness benefit inside the multi-species consortium comprised of bacterial species with lower sensitivity to trimethoprim.

## Discussion

The aim of this study was twofold. We aimed to: (I) construct a well characterized model system allowing for controlled mechanistic studies of river biofilms as well as (II) test this system by exposing it to the antibiotic trimethoprim and monitor effects of this environmental stressor.

Towards the first aim, the four strains exhibited differences in several microbiological and chemical traits, allowing potential synergies to arise between cells. The model system largely fulfilled the criteria that were set up to make it a versatile tool for mechanistic studies of bacterial biofilms. Firstly, the use of river isolates from a small fresh-water stream, previously co-isolated from one spot, makes the system environmentally relevant. The isolates could be successfully cultivated at different nutrient content both as monocultures and in coculture, providing a simplified, yet broadly and practically applicable model system for future studies of relevance to environmental biofilms. Phenotypic traits could be followed from an integrated microbiological and chemical perspective by combining microbiological assays, optical microscopy and chemical characterization methodologies such as vibrational (micro)spectroscopy. Furthermore, the genome sequence will enable future studies to link various phenotypic and biochemical responses to specific genes. Using ATR-FTIR spectroscopy, we were able to monitor changes in composition of functional groups indicative of groups of macromolecular substances. In addition, we observed changes in bands indicative of PHA, an energy storage molecule previously linked to bacterial processes, such as stress responses. These changes were observed in real time, non-destructively and in situ in the biofilms. Optical microscopy was used to gain complementary information about biofilm morphology and showed changes in biofilm architecture between co-cultures and monocultures, as well as after exposure to trimethoprim. Using Raman microspectroscopy, spatial organization of co-cultures could be investigated showing that *Sphingomonas* cells formed smaller aggregates inside the biofilm surrounded by other cells and by EPS. Thus, the model system holds potential for future studies of interactions between environmental pollutants and biofilms, as it enables investigations of both bacterial cells and EPS from a range of different angles.

Pollutants interacting with biofilms do not only interact with the bacterial cells inside the biofilm but also influence and are influenced by the EPS matrix surrounding these cells. EPS plays an important role in the response to pollutants as production of a highly hydrated hydrophilic EPS layer may protect cells to different extents from substances that are comparably hydrophobic. However, production of hydrophobic storage substances may render the biofilm matrix more hydrophobic and therefore more prone to take up hydrophobic pollutants. Thus, when studying pollutant interaction with biofilms, both the cells and the EPS need to be investigated for a more complete, holistic understanding. This necessitates well-defined model systems, where the bacteria are chosen to be environmentally relevant yet biologically varied, and possible to characterize both in terms of their microbiological, physical and chemical properties. We hope that the approach and model system presented here, provides such a tool for future studies of fresh water bacterial communities.

The second aim of this study was to use the developed model system to investigate the response of river isolates to the environmental stressor trimethoprim. This is an antibiotic that in natural waters can occur in both positively charged and neutral form. The effect of trimethoprim was investigated both on planktonic cells, monospecies biofilms as well as on a multi-species consortium from the four river isolates. Three of the four river isolates investigated were sensitive to trimethoprim in a concentration dependent manner. The effects were observed both at pH 7, where trimethoprim is neutral/positively charged, and at pH 5, where trimethoprim is predominantly positively charged. At pH 5, the bacteria encountered two stressors simultaneously: lower pH and trimethoprim. The four isolates exhibited variations in sensitivity to trimethoprim, with *Pseudomonas* being the most resistant and *Sphingomonas* the most sensitive. Vibrational spectroscopy showed that the more sensitive strains altered their biochemical composition during exposure and increased their production of PHA. These substances function as energy storage and their production is known to be linked to various stress responses in bacteria. The biofilm architecture and thickness were also affected by exposure to trimethoprim and the effect was strain dependent. In co-culture the most sensitive isolates appeared to be protected from the drug. Furthermore, the organization of cells in the four species consortium appeared to be very similar between the exposed and non-exposed condition, whereas the EPS production was decreased during stress conditions. We hypothesize that the observed biofilm heterogeneities of the multi-species consortium improved the fitness of the more sensitive *Sphingomonas* isolate during exposure to trimethoprim at pH 5. These results illustrate the benefit bacteria may experience from residing in multi-species biofilms during periods of external stress in environmental systems.

## Materials and methods

### Strains

Bacterial isolates were collected from a small stream, Knivstaån, in the south of Sweden (Latitude: 59° 43’ 32.30” N, Longitude: 17° 47’ 15.11” E). The isolates were collected in May 2018 from a sampling point downstream from a sewage treatment plant serving the small town of Knivsta (denoted Sampling point 4 in Hagberg et al.^28^) and isolated at room temperature on 10% R2A agar. To develop a model system, we used four bacterial isolates. The four isolates all originated from the same sampling spot. However, they represented only a small fraction of all species that were present in the collected sample^28^. They were selected to represent species with differences in microbiological and chemical characteristics, based on the assumption that such differences could give rise to complementary interactions between strains. From a data-driven design of experiments point of view, selecting species with larger differences covers larger variations and thus expands the potential use and scope of the model system. As the isolates originated from downstream a sewage treatment plant, they had been exposed to low levels of pharmaceuticals prior to isolation. Thus, they could be expected to have adapted their lifestyle to cope with such types of low-intensity stress factors^28^. The antibiotic trimethoprim was chosen as an environmental stressor, and monitored at two pH levels, in neutral and slightly acidic media, to observe possible alterations in interactions following an increased protonation of trimethoprim at lower pH. Trimethoprim was detected in the small stream, from where the isolates were collected, at a concentration of approximately 10 ng/L at the site and time of sampling^28^. Thus, the levels used in this study were much higher than what the bacteria presumably had been exposed to previously.

The strains were routinely grown on R2A medium (Sigma-Aldrich) developed for cultivating freshwater isolates^85^. The isolates were stored as freezer stocks and cultivated in 10% or 100% R2A liquid medium or on 10% or 100% R2A agar plates at room temperature (21 °C)^28^. Two medium concentrations were used to observe differences between nutrient levels, which may vary in fresh water. All experiments were conducted at room temperature.

Whole genome sequencing, followed by de novo assembly, was performed by Eurofins (Germany). The resulting genome sequences were compared to genomes deposited in the database at the US National Library of Medicine using the BLAST search tool^86^. The strains were deposited in the Gothenburg University Culture Collection (Sweden), in connection to publication, and assigned the reference numbers listed below. As described in the “Results” section, their genome sequence matched most closely environmental isolates of *Pseudomonas* sp (CCGU 78360), *Pararhizobium* sp. (CCGU 78362), *Rhizobium* sp. (CCGU 78361) and *Sphingomonas* sp. (CCGU 78359). In this work, these strains were described only by the genus name, i.e., excluding the sp. abbreviation, to facilitate reading, except in the section describing the results from genome analysis.

### Bacterial growth and co-existence

To follow the growth of the isolates, an automated set up was chosen using round bottom 96-well plates. The edge wells of the plate were only filled with sterile milliQ water to reduce evaporation-errors at the edges. An overnight bacterial liquid culture in 100% R2A was resuspended in fresh 100% R2A media to obtain an optical density at 600 nm (OD) of 0.002. This culture was further diluted to OD = 0.001 in the wells of the 96-well plates. The final volume in each well was 100 µL. Sterility control was included in the form of pure medium. Growth was monitored by measuring OD on an automated plate reader (BioTek Synergy 4 Hybrid Reader). Measurements were performed every 30 min with 10 s of shaking beforehand. The growth was monitored for a total of 48 h.

To establish whether the bacteria samples were able to coexist, we performed a series of cross-cultivation experiment on 10% R2A plates. Two bacteria strains grown from the frozen stock were re-streaked as intersecting lines on an agar plate. Thereafter the plates were left to incubate for 3–4 days at room temperature.

### Biofilm formation

One bacterial colony (ca 3 µL), grown on 10% R2A agar, was transferred to 2 mL 100% R2A media and left overnight at room temperature on a shaking table. The next day OD was measured and diluted with fresh R2A 100% media to obtain OD = 0.15. Suspensions of bacteria was pipetted into round-bottom-96-well plates to give a total of 150 µL bacterial suspension in each well. The cells were cultivated for 24 h in static conditions. After that, the suspensions were removed, and the wells washed three times with sterile 150 µL phosphate buffered saline. A volume of 150 µL of crystal violet (CV) was pipetted into each well and left to stain remaining biofilm for 10 min. After this, the staining solution was removed, and the plate was filled with 150 µL of 70% ethanol. The measurements of OD were performed on a plate reader (Perkin Elmer Wallac 1420 Victor 2 Microplate Reader).

### Motility

R2A plates (25 mL) were prepared with different concentrations of agar: 0.3% agar for swimming, 0.5% agar for swarming, 1% agar for twitching. A bacterial suspension was prepared from a single colony grown on a standard 100% R2A agar plate that was transferred into 3 mL of R2A 100% media. After overnight growth, OD of the culture was measured and diluted with fresh R2A 100% media to obtain OD = 0.15. For swarming, a drop of 5 μL bacterial suspension was placed at the top center of the R2A agar plate (100% R2A). For twitching, the agar was stabbed with the pipet, and the drop of suspension was placed at the bottom of the Petri dish. For swimming, the drop of the suspension was injected inside the agar. The plates were cultivated for 72 h. Every 24 h, the diameter of the drop area was measured at the longest distance. Four replicas were done for each type of motility (from two different cultures).

### Cell morphology

To study cell morphology, SEM and AFM were used. For SEM analyses, bacterial cells were grown on 10% R2A agar and thereafter chemically fixed using 2.5% glutaraldehyde in 0.1 M phosphate buffer pH 7.4 either attached to a small cube of agarose from the agar plate or in suspension. *Pseudomonas* and *Rhizobium* were easily detached from the agar and were therefore analyzed from suspension. Cells in suspension were sedimented onto a poly-L-lysine coated coverslip. Samples were washed and dehydrated through a series of ethanol baths. Thereafter, samples were critical point dried using a CPD300 (Leica microsystems, Wetzlar, Germany) and sputter coated with 5 nm of Iridium with a Quorum Q150T-ES (Quorum technologies, Laughton, East Sussex, UK) to avoid charge build-up on the surface of the sample during analysis. Samples were analyzed on a Zeiss Merlin SEM (Carl Zeiss AB, Stockholm, Sweden) at an accelerating voltage of 5 kV, beam current of 150 pA and detected with an InLens secondary electron detector. Cell length, width and volume were measured from SEM images on ten randomly chosen cells using the JMicroVision software^87^.

AFM imaging was done on bacteria collected from individual suspensions of the four strains obtained as follows. Colonies from a working stock (100% R2A agar plates) were inoculated into 100% R2A broth (20 mL in 100 mL Erlenmeyer flask). The cultures were grown in presence of mechanical shaking (50−100 rpm), at 21 °C for 42 h to 65 h depending on the strain, resulting in optical densities between 0.65 and 1.80. A volume of 200 µL of suspension was deposited on a glass disk coated with polyethylene imine and left to settle for 30 min. Thereafter the bacteria were fixed with glutaraldehyde (12%) for 2 h, subsequently the slides were rinsed by dipping in sterile water and finally air dried. AFM measurements were performed using a Drive AFM (Nanosurf AG, Zurich, Switzerland). The topography of bacteria was obtained by AFM operating in wavemode NMA. Silicon nitride AFM probes purchased from Nanosurf AG (WM 0.3 pt) with spring constant of about 0.4 N/m were used for all analyses. All images were recorded with a resolution of 1000 × 1000 pixels and a scan rate of 1 Hz.

### Biochemical composition from infrared spectroscopy

Biofilm formation and changes in the biochemical composition of bacterial isolates were monitored in situ using attenuated total reflection Fourier-transform infrared (ATR-FTIR) spectroscopy, using a custom-built measurement cell^88^. Prior to the measurements, the ATR-FTIR cell was cleaned by alternate bathing in 0.1% (w/v) sodium dodecyl sulfate solution for 20 min and 0.1 M solution of hydrochloric acid (VWR) for 20 min, with magnetic stirring. Thereafter, the measurement cell was transferred into a microbiological safety cabinet, rinsed with sterile milliQ water and mounted with a zinc selenide (ZnSe, Harrick Scientific Products, US) crystal, serving as a substrate for the biofilm and as the internal reflection element of the ATR-FTIR spectroscopic measurements. The mounted cell was disinfected by exposure to 70% ethanol for 1 h, after which the cell was rinsed again with sterile milliQ water and filled in with the bacterial suspension. Bacterial suspensions for biofilm experiments were prepared using cultures of isolates grown for 16 h in 50 mL of 100% R2A medium in 100 mL Erlenmeyer flasks with gentle agitation on a platform shaker (Heidolph polymax 2040). The cultures were centrifuged at 4000 rpm for 12 min, and the obtained pellets were resuspended in 40 mL of 100% R2A medium in the ATR-FTIR measurement cell at a cell optical density of 0.02. The measurement cell was thereafter installed in the FTIR spectrometer (Vertex 80 v, Bruker). Spectra were recorded at static conditions in the room conditioned at 25 °C on at least two sample replicates. The resolution of the single beam spectra was 4 cm^−1^, 200 scans were collected per spectrum. Spectra were recorded every 5 min during the first 3 h of each experiment and thereafter every 20 min for the remaining time of the experiments. Spectra of cells grown on culture plates were analyzed using a Bruker Platinum accessory with a diamond internal reflection element, by placing biomass directly onto the crystal after cultivation on agar for 3 days, using the same spectrometer and the same spectral parameters.

Spectra were processed in OPUS 7.8 to remove atmospheric contributions and adjust the baseline at 3580, 2750, 1800, and 900 cm^−1^. For analyses involving integrated intensities, spectra were processed in Matlab R2023b for baseline correction with no preliminary spectral scaling. Baseline was calculated as a straight line between zero spectral intensities at 1800 and 900 cm^−1^. Integrated intensities were calculated relative to this baseline. Integrated intensities of the region between 1271 cm^−1^ and 1192 cm^−1^, representing phosphates present in nucleic acids and phospholipids, respectively, were used to illustrate bacterial growth on the ATR crystal. Changes in polyhydroxyalkanoate (PHA) were monitored using the carbonyl band intensity between 1755 and 1705 cm^−1^. Proteins were determined using integrated intensities of the amide II band (1591–1487 cm^−1^). The ratio of PHA to amide II was chosen to illustrate changes in PHA accumulation due to possible interference of PHA bands in the phosphate region. The integrated intensities were calculated using the background recorded at 3 h for *Rhizobium* and *Sphingomonas* (as in Fig. 5). However, a background recorded after 5 min after bacterial inoculation was used for *Pseudomonas* and *Pararhizobium* to avoid negative integrated intensities due to initial bacterial attachment and detachment early in the experiment.

For measurements of biochemical changes in planktonic cultures exposed to trimethoprim, bacterial cultures were grown for ~16 h in 50 mL of R2A medium in 100 mL Erlenmeyer flasks with slight agitation on a platform shaker (Heidolph polymax 2040). Thereafter, the cultures were harvested by centrifugation at 4000 rpm for 12 min and resuspended at OD of 0.02 in fresh R2A medium containing 25 mg/L trimethoprim. Plastic 50 mL test tubes containing 25 mL of bacterial suspensions were used for these incubations. Cultures were incubated for 24 h at room temperature. Cultures in R2A medium without trimethoprim were prepared as controls. After 24 h of incubation, cultures were washed with 0.9% NaCl two times, and bacterial pellets were placed on the ATR crystal to record spectra using 0.9% NaCl as a reference. Experiments were performed on duplicate samples. Measurements were done on a Vertex 80 v FTIR spectrometer (Bruker) with a Bruker Platinum attenuated total reflectance (ATR) accessory with a diamond internal reflection element. Spectra were recorded between 4000 and 700 cm^−1^ with 4 cm^−1^ resolution for single beam spectra and 200 scans per spectrum. The collected spectra were processed in OPUS 7.8 for water vapor and baseline correction. Baseline was calculated as a straight line between 3580, 2750, 1800 and 900 cm^−1^. The presented spectra were normalized to the intensity of the amide II band (1592–1486 cm^−1^).

### Spatial distribution from Raman microspectroscopy

For individual isolates grown on agar, samples were prepared by growing cells on 10% or 100% R2A agar plates for 3 days. For four species consortia, the culture was prepared as follows. Each colony from a R2A 100% agar plate was suspended in 40 mL R2A 100% liquid media and left for 24 h on a benchtop shaker at room temperature. The next day, OD was measured, and each bacterial solution was diluted equitably in fresh R2A 100% media to obtain a final OD of 0.02. Finally, 200 µL of the final solution was disposed in 100% R2A agar petri dish and the four-species consortia cultivation was done in 3 days. For measurements, a colony of cells was picked from the agar plate and placed on a Raman-grade calcium fluoride slide (Crystran Ltd, UK). Raman spectra were recorded using a Renishaw Qontor Raman spectrometer using a 532 nm laser and a 100× Leica objective. Spectra were recorded with 100% laser power (50 mW nominal power at laser exit) with the center set at 1250 cm^−1^ with 1 s (*Sphingomonas* and four species consortia), 10 s (*Pseudomonas* and *Rhizobium*) or 25 s (*Pararhizobium*) exposure times. *Pseudomonas* and *Rhizobium* samples were bleached for 30 s before spectral recording. Spectra were noise-filtered and baseline-corrected in Renishaw’s WiRE software (version 5.3). Experiments were performed on duplicate samples, each measured at a minimum of two different locations. Raman microspectroscopy of four species consortia exposed to 25 mg/L trimethoprim was done in the same way with the exception that after cultivation for 1 day on agar, 0.5 mL of 25 mg/L trimethoprim solution was added in order to completely cover the bacterial biofilm. After 2 days of growth, a colony of cells was picked from the agar plate and placed on a Raman-grade calcium fluoride slide for measurements.

Two types of multivariate data analyses were performed for the hyperspectral maps to ensure robustness of the interpretation of hyperspectral images. Both analyses assume the spectra to be a linear combination of “pure” spectral components (or at least classes of compounds, such as proteins, carbohydrates, etc) with intensities proportional to the abundance of those components in the sample. First the contribution of carotenoids from *Sphingomonas* and cytochromes from the other strains in the four species consortia were determined using MCR-ALS analysis (following asymmetrical least squares baseline correction with a lambda = 10^6^ *p* = 0.001, and total area normalization), using only non-negativity constraints (both for spectra and concentration)^89^. In addition, Bayesian positive source separation (BPSS) was also used^90,91^.

### Surface chemical composition from cryo-X-ray photoelectron spectroscopy

Surface chemical composition of bacterial biomass (bacteria and EPS) grown on 10% R2A agar plates was acquired using cryo-X-ray photoelectron spectroscopy (cryo-XPS) on Kratos Axis Ultra DLD spectrometer, as previously described for bacteria from culture plates^92^. The biomass was collected with a cultivation loop directly from the agar plate after 2 days of growth, brought to a temperature of −170 °C on the sample holder inside the XPS spectrometer, using liquid nitrogen cooling, and thereafter analyzed frozen. Two biological replicas were analyzed for each strain.

### Biofilm morphology from confocal microscopy

Biofilms were prepared in Lab-Tek^®^II chambered #1.5 German cover glass systems (Nunc^®^, Thermo Fisher Scientific) containing 8 wells. Wells were filled with 0.5 mL of 100% R2A medium before inoculation of cultures. Bacterial suspensions for biofilm experiments were prepared using cultures of isolates grown for 16 h in 50 mL of 100% R2A medium in 100 mL Erlenmeyer flasks with slight agitation. These cultures were centrifuged at 4000 rpm for 12 min, diluted in fresh 100% R2A medium and added in the chambered cover glass slides. The OD at the start of biofilm culture growth was 0.02. For biofilms composed of multiple species, the sum OD was 0.02, into which isolates contributed equally. Biofilms were cultivated for 72 h at room temperature and washed with fresh 100% R2A medium three times before staining. *Bac*Light^TM^ kit (Invitrogen), containing Syto 9 and propidium iodide, was used to stain the cells. Ten-fold diluted (NaCl (VWR) 0.9% w/v) manufacturer solutions of the dyes were added in 100% R2A medium for cells staining (1.5 µL of 1/10 Syto 9 and 1.5 µL of 1/10 propidium iodide in 100 µL of 100% R2A). Biofilms were stained for 15 min in the dark and washed once with fresh R2A medium to remove dye excess. After staining of the biofilms, microscopy images were recorded using Nikon A1R confocal microscope controlled by Nikon NIS Elements interface. For excitation in blue and emission in green, a laser at 488 nm was used, giving emission in the range 500–550 nm. For excitation in green and observation in red, a laser wavelength of 561 nm was used giving emission in the range 570–620 nm. The same methodology was used for unexposed biofilms and biofilms exposed to 25 mg/L of trimethoprim.

In parallel experiments, biofilms of *Pseudomonas* were stained with CTC *Bac*Light™ RedoxSensor™ kit (Invitrogen) using the manufacturer protocol to estimate the activity of cell respiration chains under conditions with and without trimethoprim. DAPI dye provided in the kit was used for counterstaining.

Images were analyzed using BiofilmQ^93^ to obtain quantitative measures of biofilm biovolume and ratio between propidium iodide and Syto 9 intensity as a measure of the proportion of cells with damaged membranes. The threshold in images for separating cells from the background was obtained using the Otsu method available in the BiofilmQ software with the sensitivity marker 0.2; the threshold was visually verified for a correct separation between biofilm cells and the background. Image processing was performed without biofilm segmentation into voxels. “Global biofilm properties” and “Fluorescent properties” were calculated for the information on the biofilm biovolume, height, and mean intensity of Syto 9 and propidium iodide stains. Biofilm volume represents the quantified volume in µm^3^ of parts of 3D reconstructed CLSM images corresponding to bacterial cells after separating from the background by applying a threshold. Fluorescence properties were calculated to obtain mean intensity of Syto 9 and propidium iodide in the determined biovolume, thereafter the ratio of the mean intensities was calculated.

### Sensitivity of planktonic isolates to trimethoprim

Stock solutions of trimethoprim (Sigma-Aldrich) were prepared in milliQ water (2 g/L or 20 g/L) with 1% (V/V) acetic acid (Merck) to increase solubility of trimethoprim. The solution was stored at 4 °C covered with metal foil, to prevent degradation from light. This stock solution was diluted with sterile milliQ water to desired concentrations.

Bacterial solutions were prepared as follows. One colony (ca 3 µL) from a R2A 10% agar plate was suspended in 3 mL R2A 100% liquid media and left for 24 h on a benchtop shaker at room temperature. The next day, OD was measured, and each bacterial solution was diluted with fresh R2A 100% media to obtain OD = 0.02. The bacterial cultivation was done in 24-well plates in 100% R2A. The wells included rows of sterility controls, positive controls of bacterial growth in pure medium, bacterial growth in medium with trimethoprim, as well as bacterial growth in media with acetate at concentrations corresponding to wells with trimethoprim. The plates were left without shaking at room temperature for 24 h covered in metal foil to avoid light exposure. The effect of trimethoprim on bacterial growth was estimated based on reduction of OD.

In addition to the one-point OD measurements described above, growth inhibition was monitored by measuring OD on an automated plate reader (BioTek Synergy 4 Hybrid Reader) every 30 min for a total of 48 h. In the round bottom 96-well plates, the edge wells of the plate were filled with sterile milliQ water to reduce evaporation-errors at the edges. An overnight bacterial liquid culture was resuspended in fresh 10% R2A media to obtain an OD of 0.002. This culture was further diluted to OD = 0.001, corresponding to approximately 3–7 × 10^5^ colony forming units per mL (CFU/mL), in the wells of the 96-well plates. The final volume in each well was 100 µL. The multi-well plate contained rows of wells for: sterility control, growth control in pure medium, and a dilution cascade giving final concentrations of trimethoprim ranging from 50 mg/L to 1.5 mg/L. The growth was monitored at room temperature with 10 s of shaking before each measurement.

## Supplementary information

Supplementary material TMP 2026-02-06

Pararhizobium 4_30.contigs

Pararhizobium 4_30

Pseudomonas 4_18.contigs

Rhizobium 4_19

Sphingomonas 4_06.contigs

## Data Availability

Supplementary information can be downloaded from the journal webpage. Additional raw data is available via Zenodo with 10.5281/zenodo.17856505. Microbial strains have been deposited and are available via Gothenburg University Culture Collection (https://www.ccug.se/).
