# Supplementary material for: Probing biofilm development, stress response and heterogeneity—spectroscopic characterization of single and multi-species consortia
Source: NPJ Biofilms Microbiomes. 2026 May 20;12:98. doi: 10.1038/s41522-026-01010-x (PMC13190702; doi:10.1038/s41522-026-01010-x)
Supplement: Supplementary file 1 — Supplementary material TMP 2026-02-06 [file 41522_2026_1010_MOESM1_ESM.pdf]

Probing biofilm development, stress response and heterogeneity - spectroscopic characterization of single and multi-species consortia

Elena Yunda[1,2], Aleksandra Hagberg[1,2], Thibault Duteil[1,2], Grégory Francius[3], András Gorzsás[1,4], Fabienne Quilès[3], Madeleine Ramstedt[1,2]*

1. Department of Chemistry, Umeå University, 901 87 Umeå, Sweden
2. Umeå Centre for Microbial Research, Umeå University, 901 87 Umeå, Sweden
3. Université de Lorraine, CNRS, LCPME, F-54000 Nancy, France
4. Science for Life Laboratory, Umeå University, 901 87 Umeå, Sweden
*= corresponding author

Supporting information

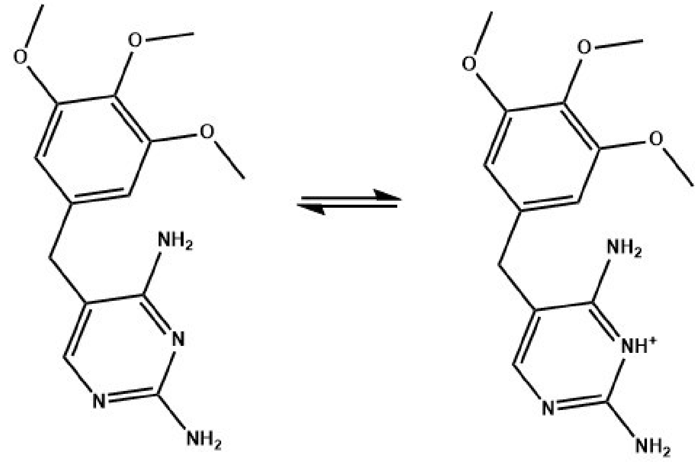

Supplementary Figure 1. The drug molecule trimethoprim and its protonation below pH 7.4 [1]

Reference for Supplementary Figure1

[1] M. Fresta, P.M. Furneri, E. Mezzasalma, V.M. Nicolosi, G. Puglisi, Correlation of trimethoprim and brodimoprim physicochemical and lipid membrane interaction properties with their accumulation in human neutrophils, Antimicrob Agents Chemother 40(12) (1996) 2865-73.

Growth curves from 96-well plates

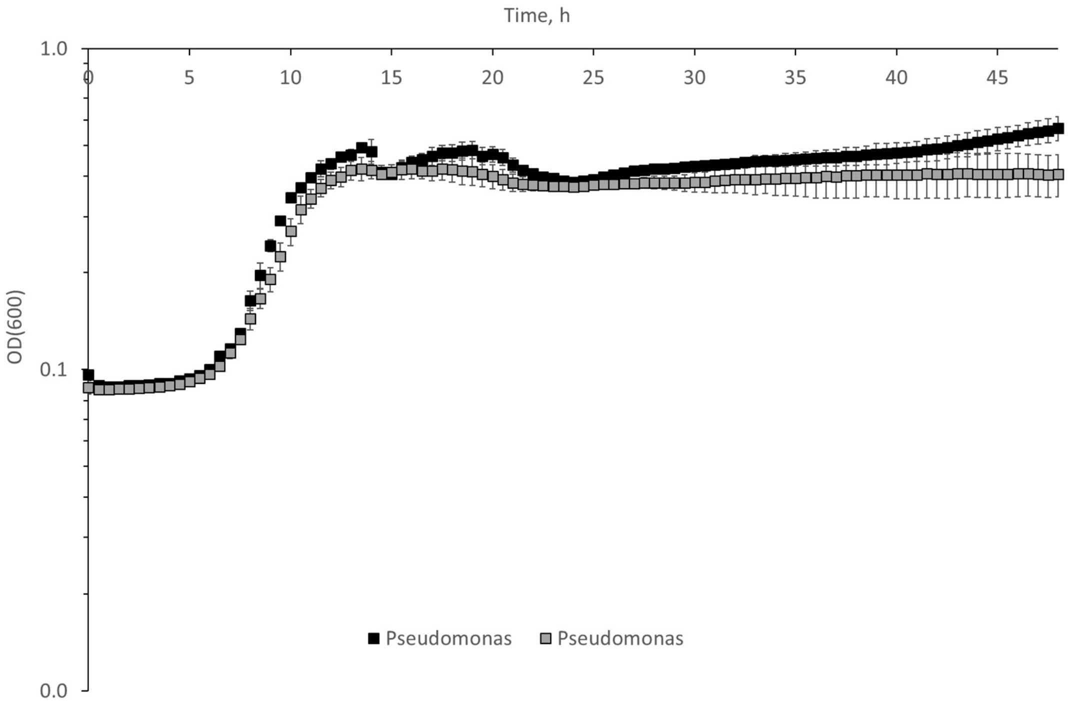

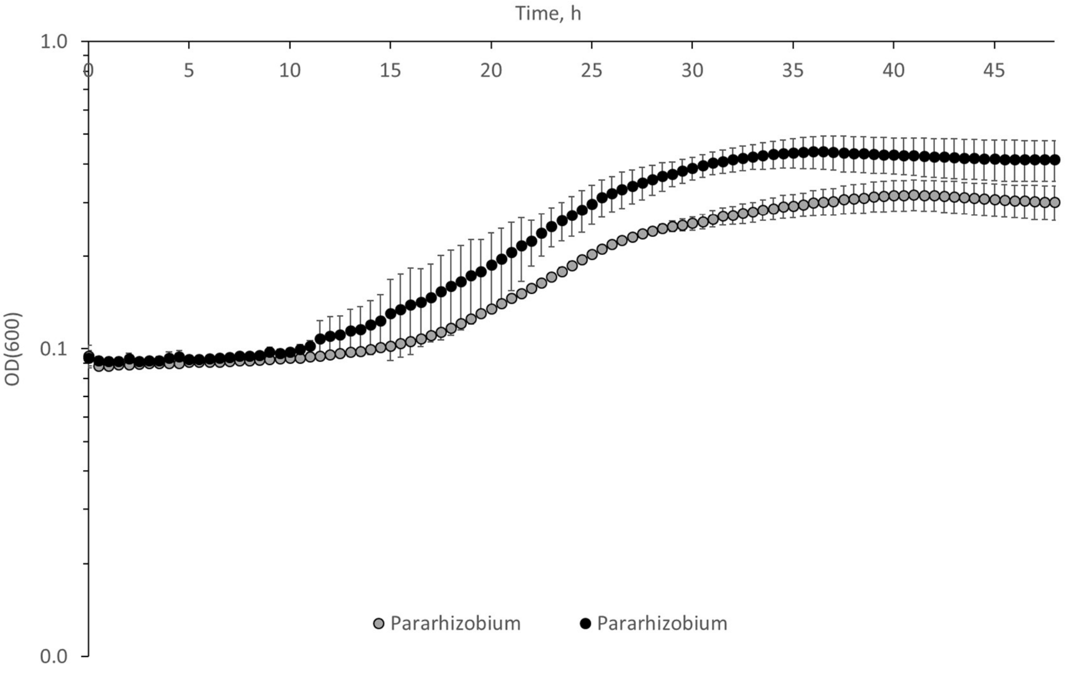

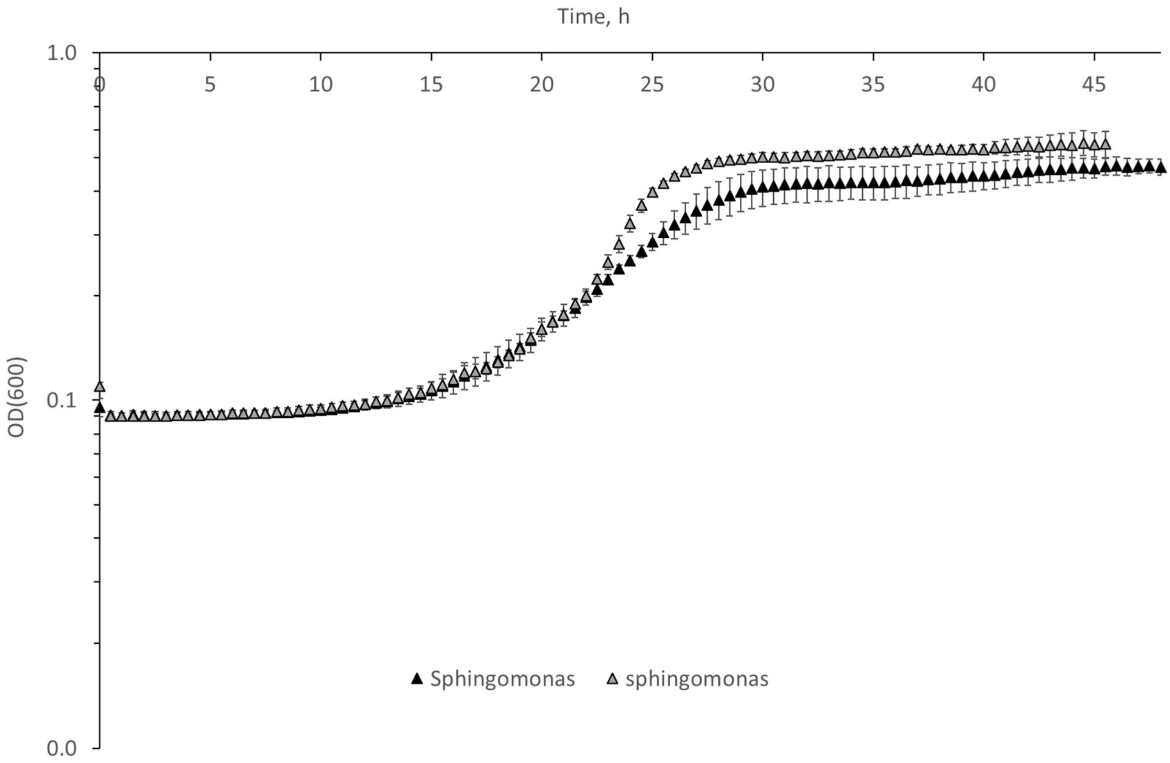

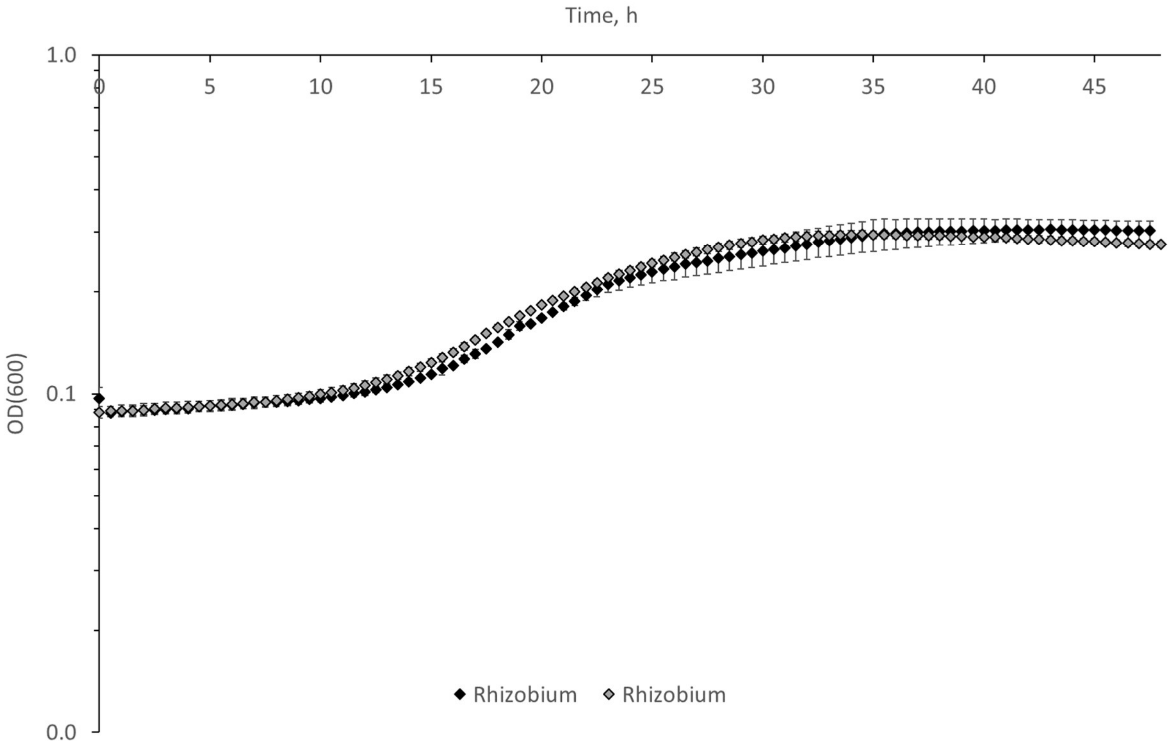

Supplementary Figure 2. Replicates of growth curves for the four river isolates in 96-well plates. Error bars represent standard deviation between 6 wells in 96-well plates. The y-axis displays the log10 value for OD(600).

Growth morphology on R2A agar

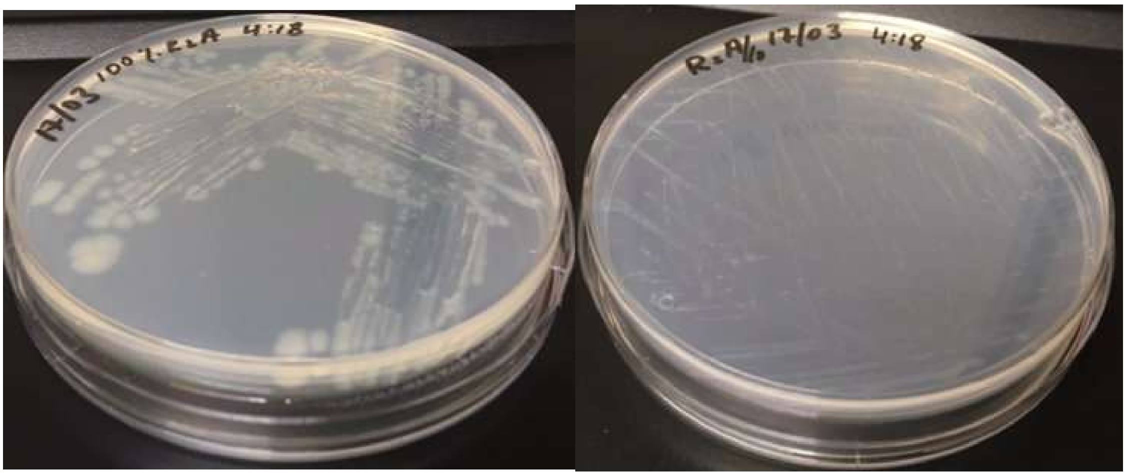

Pseudomonas on 100 % and 10 % R2A agar

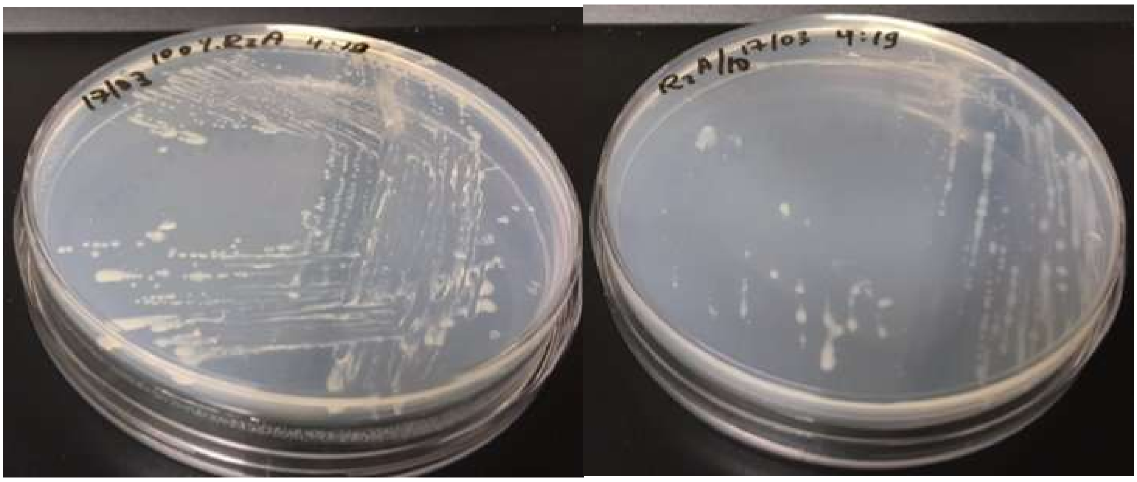

Rhizobium on 100 % and 10 % R2A agar

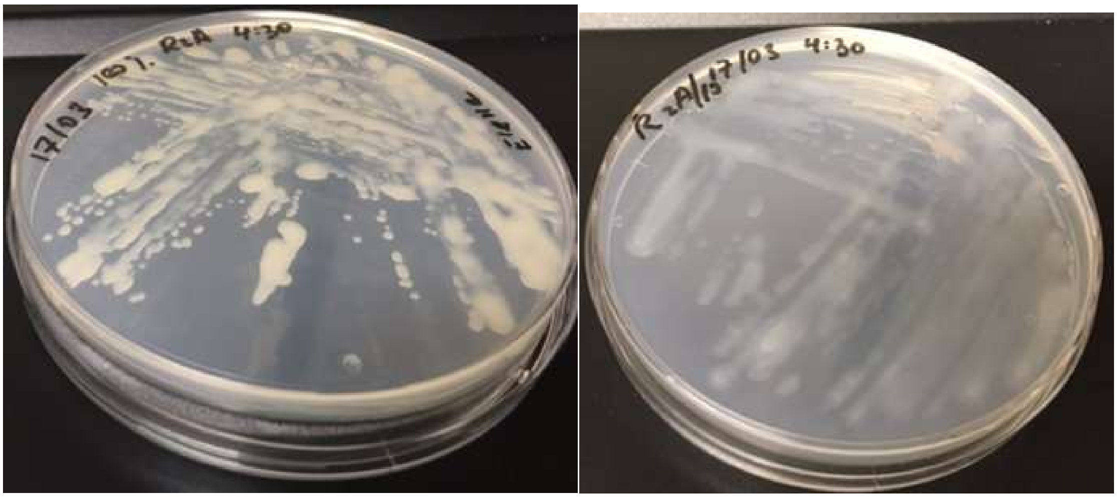

Pararhizobium on 100 % and 10 % R2A agar

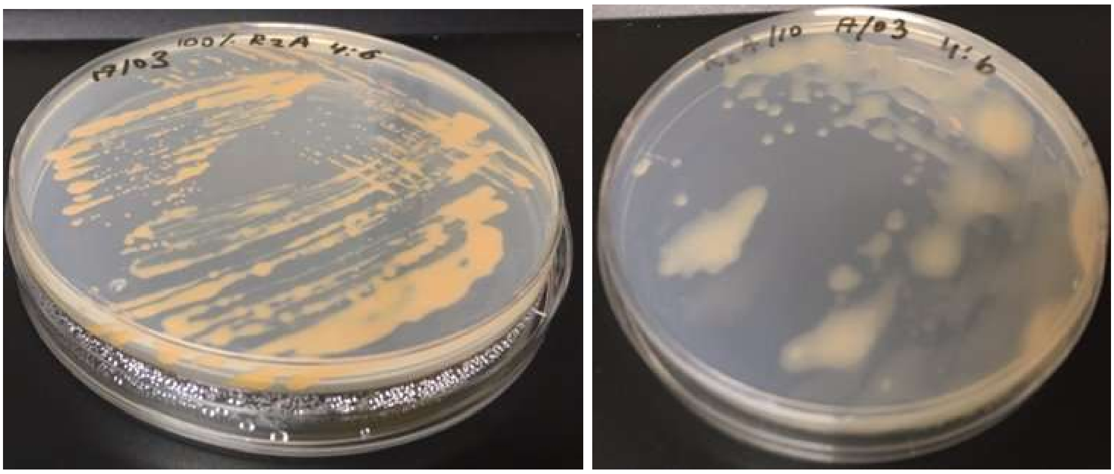

Sphingomonas on 100 % and 10 % R2A agar

Supplementary Figure 3. Growth morphology on 100 % and 10 % R2A agar plates for Pseudomonas, Rhizobium, Pararhizobium and Sphingomonas

More SEM images

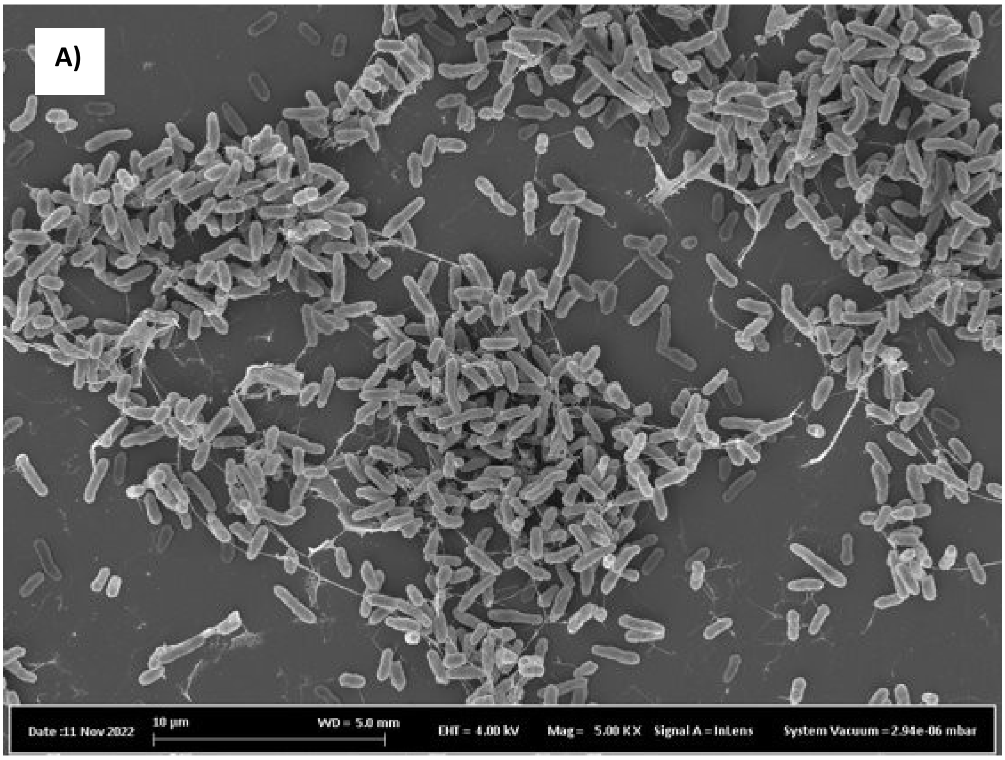

Rhizobium (scale bar 10 µm)

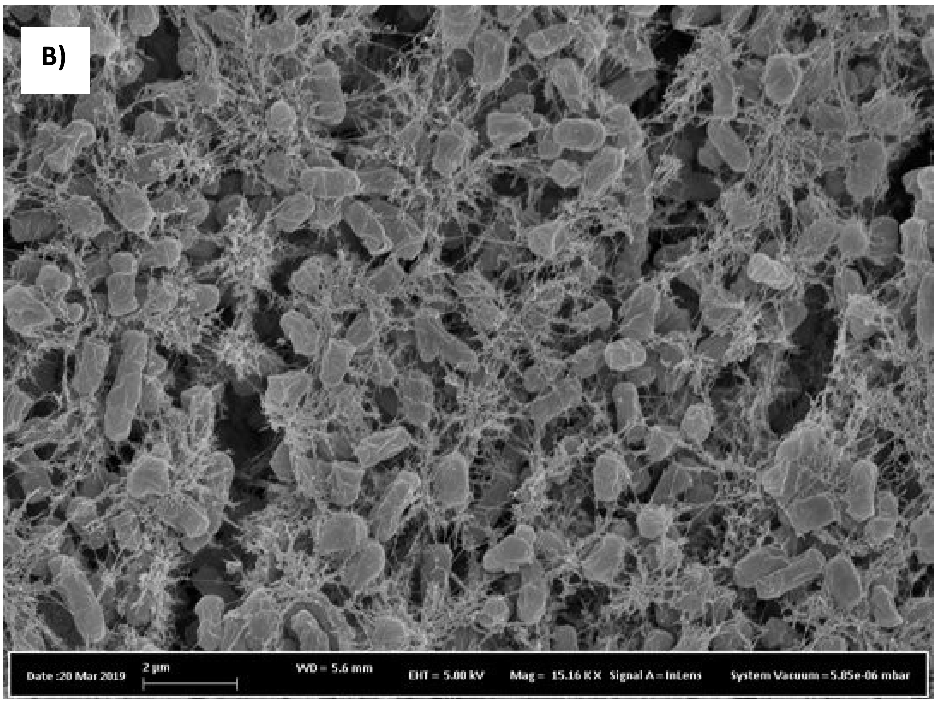

Pararhizobium (scale bar 2 µm)

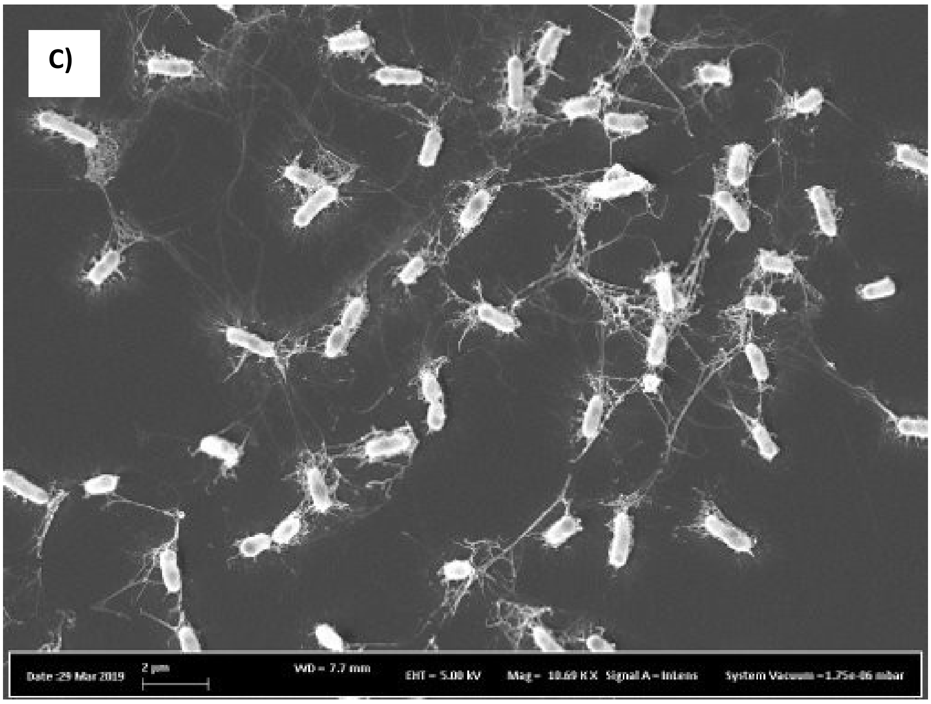

Sphingomonas (scale bar 2 µm)

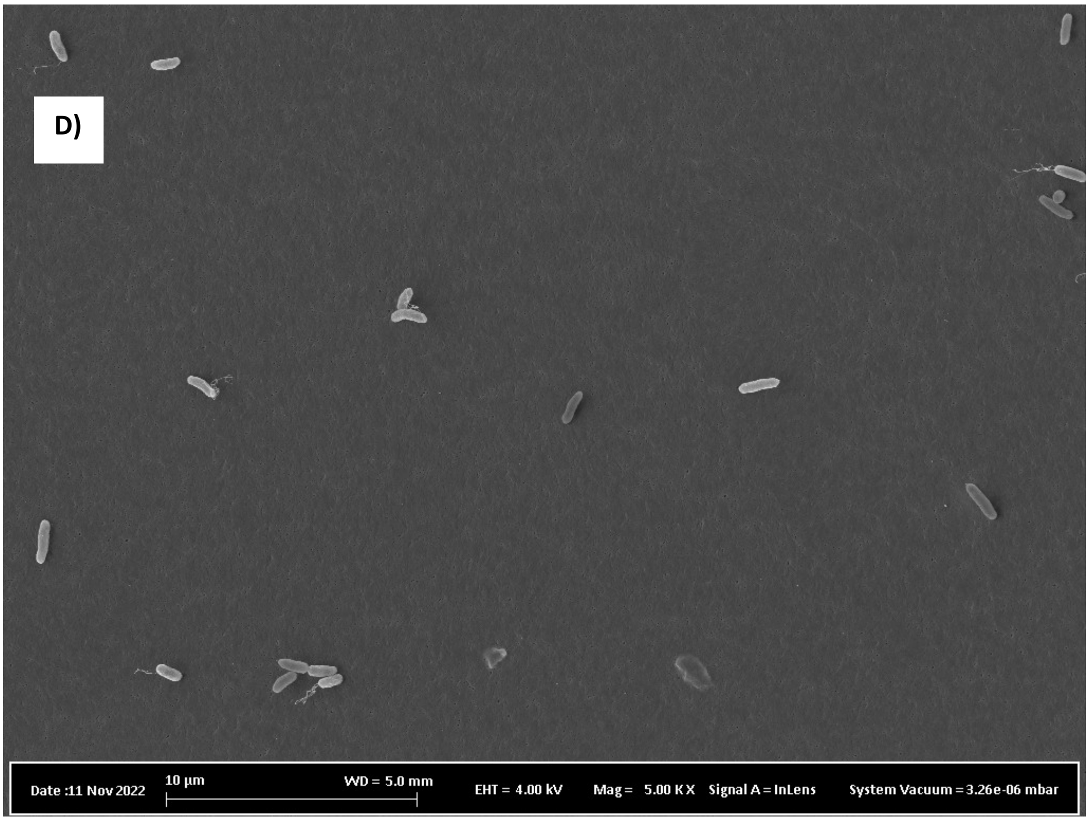

Pseudomonas (scale bar 10 µm)

E)

Cell size	cell length (µm)	cell width (µm)	cell volume (µm³)
Rhizobium	1.78 +/- 0.40	0.48 +/- 0.04	0.93 +/- 0.26
Pararhizobium	1.59 +/- 0.39	0.55 +/- 0.05	0.94 +/- 0.23
Sphingomonas	1.40 +/- 0.21	0.46 +/- 0.03	0.69 +/- 0.09
Pseudomonas	1.22 +/- 0.22	0.41 +/- 0.03	0.55 +/- 0.08

Supplementary Figure 4 SEM images with lower magnification. A) Rhizobium (scale bar 10 µm); B) Pararhizobium (scale bar 2 µm); C) Sphingomonas (scale bar 2 µm); D) Pseudomonas (scale bar 10 µm); E) Cell sizes measured on ten randomly chosen cells.

XPS C 1s spectra of river strains

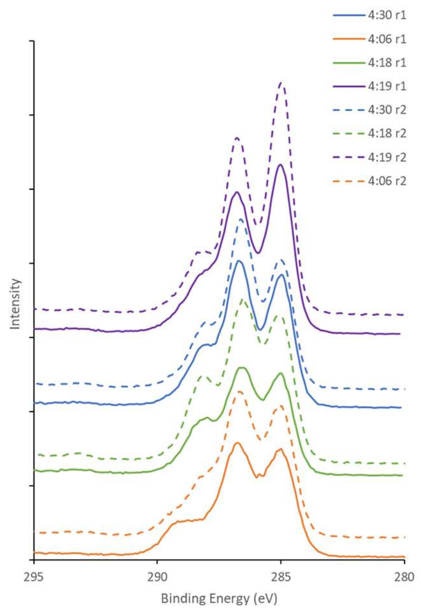

Supplementary Figure 5. XPS C 1s spectra of river strains grown on 10% R2A agar. Purple curves represent two biological replicas from Rhizobium, blue curves represent Pararhizobium, green Pseudomonas and orange Sphingomonas.

Vibrational Spectroscopy

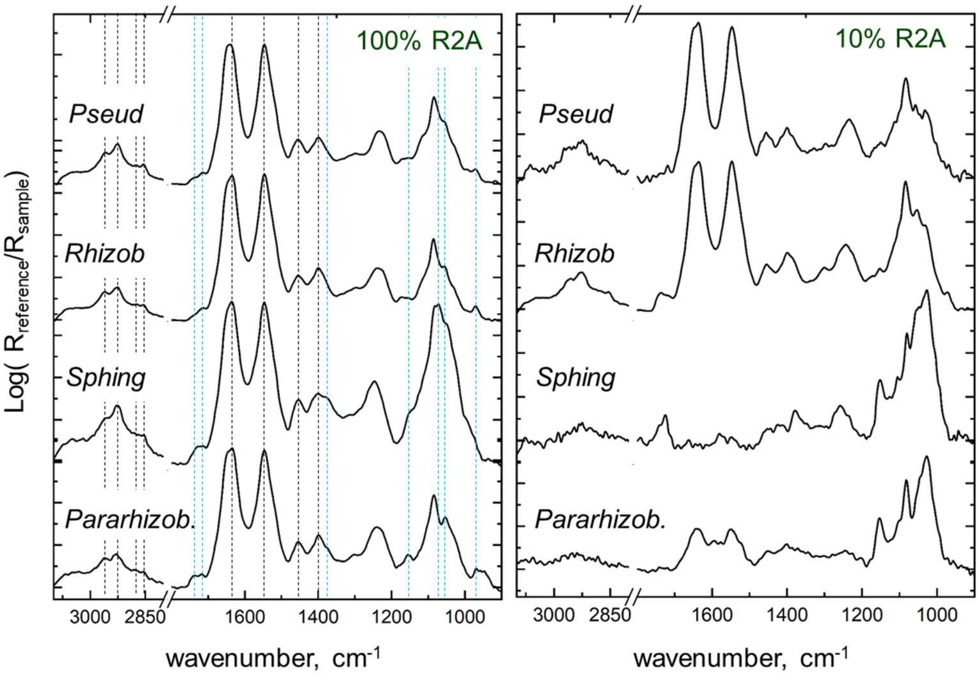

Supplementary Figure 6. Comparison of FTIR spectra of Pseudomonas, Rhizobium, Sphingomonas and Pararhizobium after growth on agar plates with 100% R2A and 10% R2A.

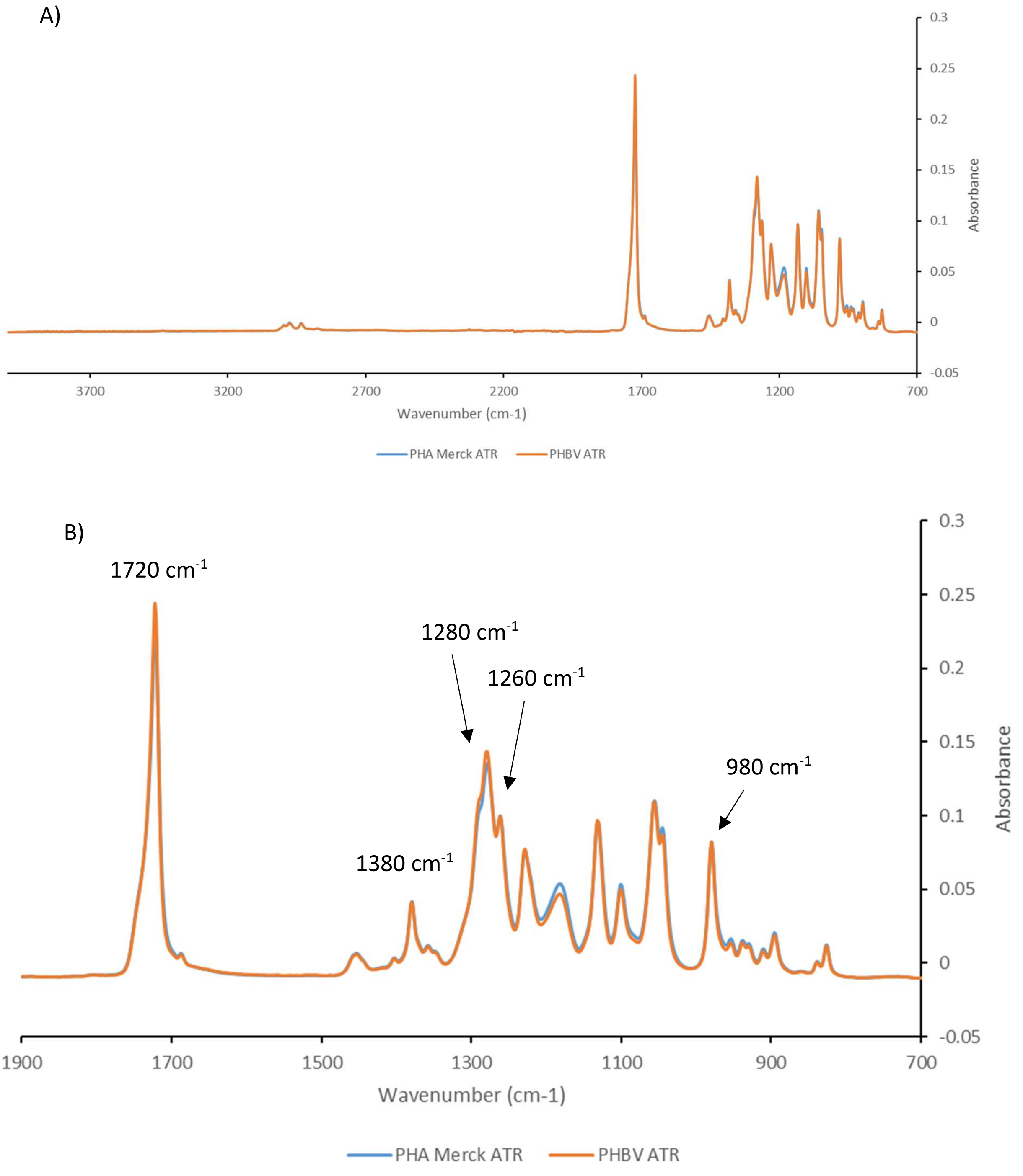

Supplementary Figure 7. A-B) ATR-FTIR spectra of two PHA reference compounds. Poly (3-hydroxybutyric acid) (blue line, PHB, obtained from Merck) and poly (3-hydroxybutyric acid-co-3 hydroxyvaleric acid) (orange line, PHBV, obtained from Natureplast, Mondeville, France (PHI 003)). Spectra acquired using the same setup as used for bacterial biofilm in Figure 4. In B, the x-axis is changed to better show the fingerprint region.

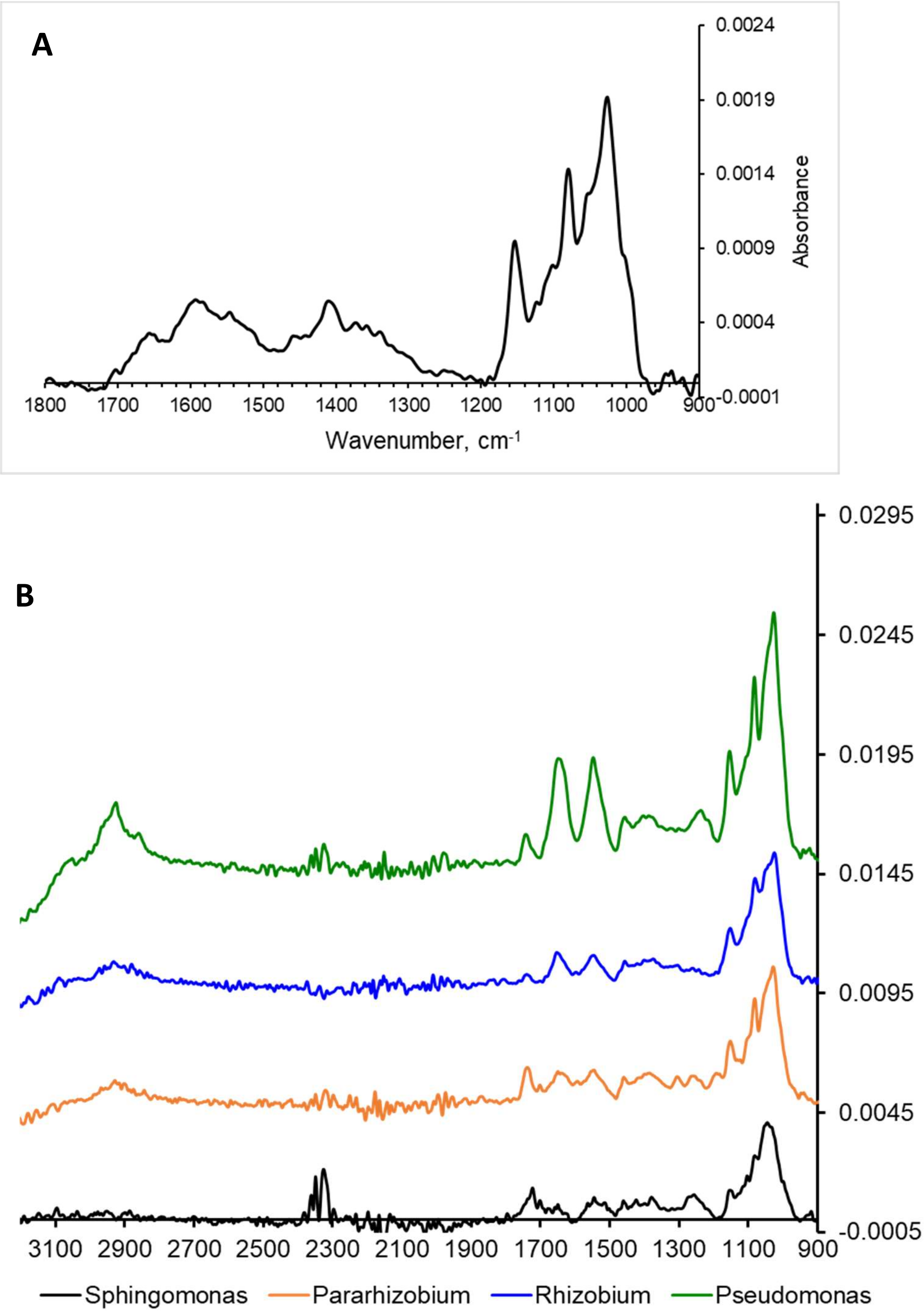

Supplementary Figure 8. A) ATR-FTIR spectrum of R2A medium recorded with ultrapure water as a reference. The pronounced bands between 1000 and 1200 cm^{-1} are assigned to starch present in the medium. B) ATR-FTIR spectra of bacterial isolates after their growth on 10 % R2A agar plates that did not contain starch in the medium composition. The spectra of bacteria and EPS nonetheless show carbohydrate content in the region between 1000 and 1200 cm^{-1}. Green spectrum represents Pseudomonas, blue Rhizobium, orange Pararhizobium and black Sphingomonas.

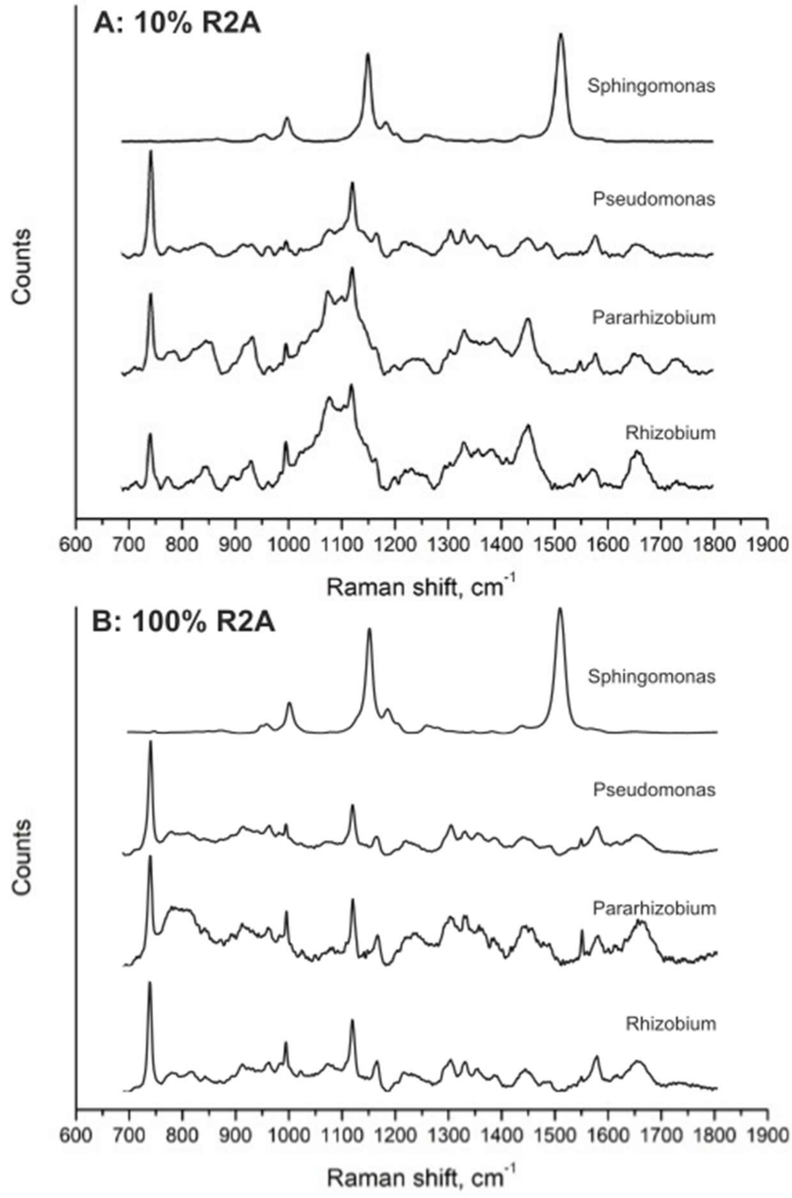

Supplementary Figure 9. Raman spectra of river isolates in A) 10% R2A and B) 100% R2A. The main contribution in the spectra of Pseudomonas, Pararhizobium and Rhizobium is from cytochromes, whereas Sphingomonas spectra show carotenoids

Biofilm assay

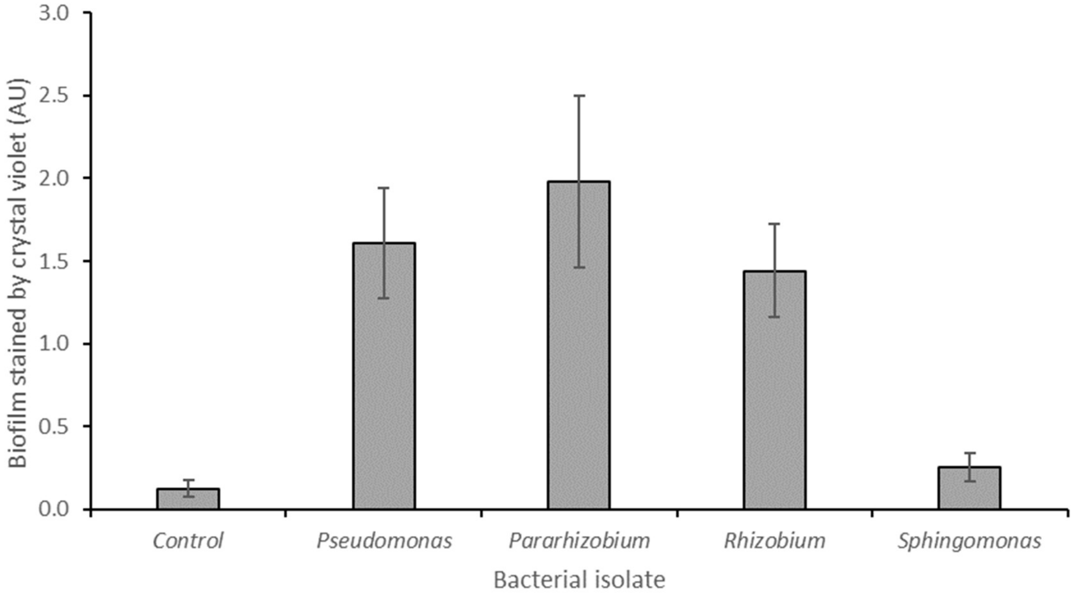

Supplementary Figure 10. Crystal violet staining of biofilms (as measured by absorption units of crystal violet stain). Error bars represent standard deviation between at least 24 wells distributed on at least four plates. Control represents a negative control without bacterial cells.

Growth curves in presence of trimethoprim at pH 7

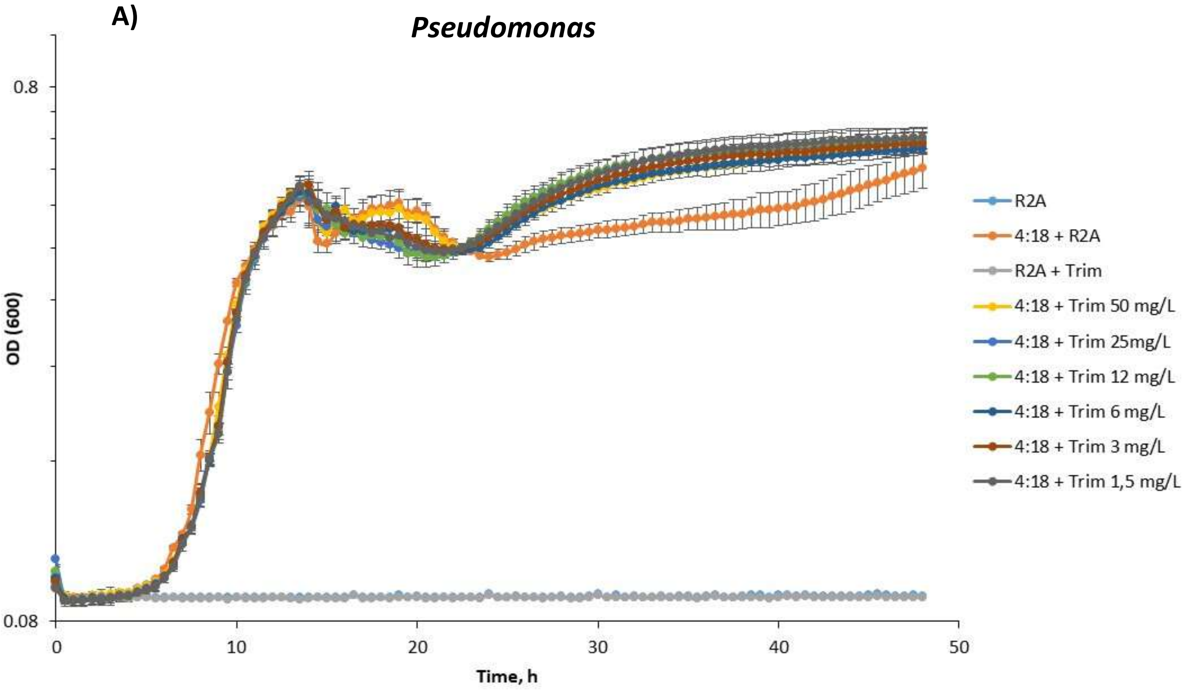

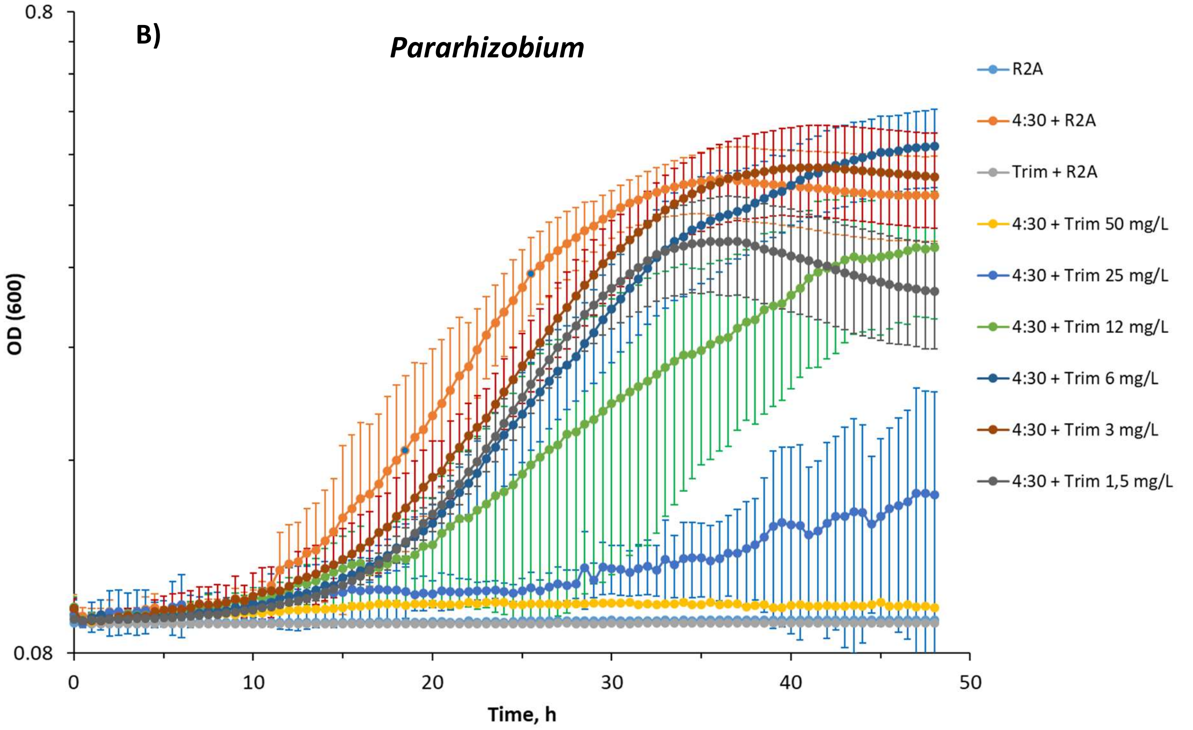

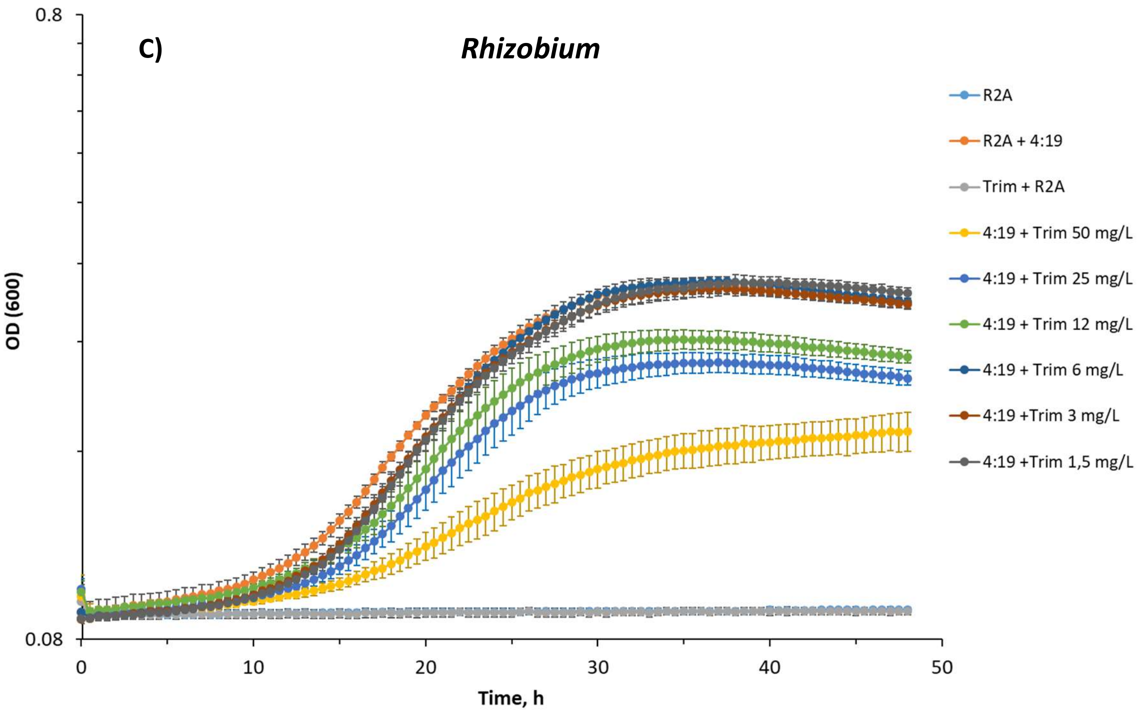

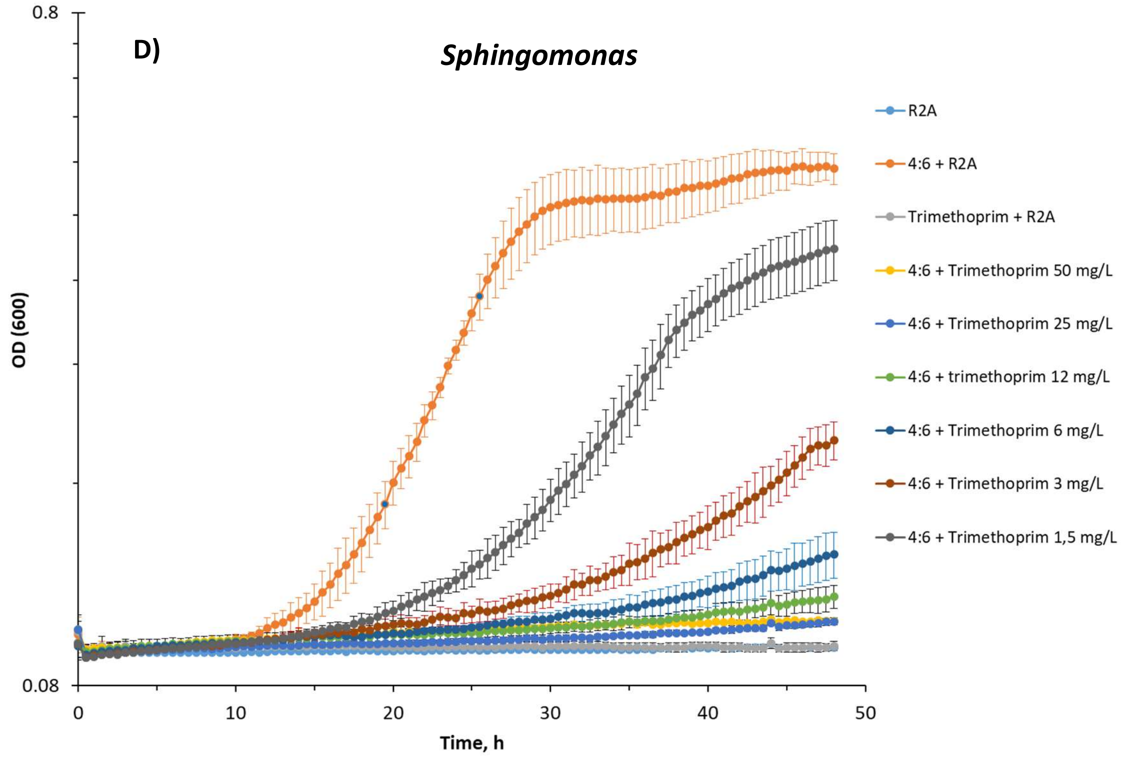

Supplementary Figure 11, Growth curves for the four isolates in 96-well plates in presence of trimethoprim at pH 7. A) Pseudomonas in 100 % R2A. B) Pararhizobium in 100 % R2A. C) Rhizobium in 100 % R2A. D) Sphingomonas in 100 % R2A. Error bars represent standard deviation between 6 wells in a 96-well plate. The y-axis displays the log10 value for OD(600).

Average cell growth at pH 5 after 24h

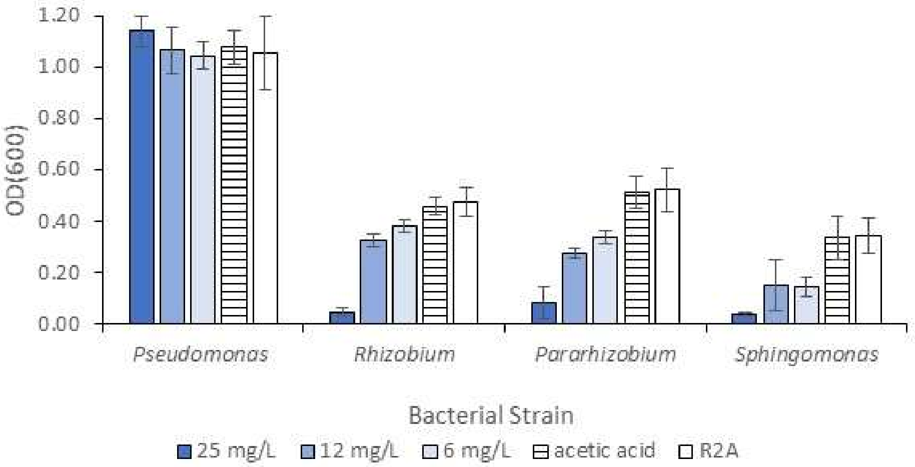

Supplementary Figure 12, Average growth of planktonic bacteria after 24h incubation in absence and presence of trimethoprim at pH 5. Error bars represent the standard deviation for growth in 12-24 wells from two biological replicates for Sphingomonas and one for the other strains. No significant difference was observed for the growth of Pseudomonas at pH 5. The growths of all other strains were significantly reduced compared to control at all concentrations (p<<0.01). No significant changes were observed for acetic acid controls at pH 5 (acetic acid concentration corresponding to 25 mg/L). The difference between Rhizobium, Pararhizobium and Sphingomonas at 25 mg/L was not significant (p>0.01).

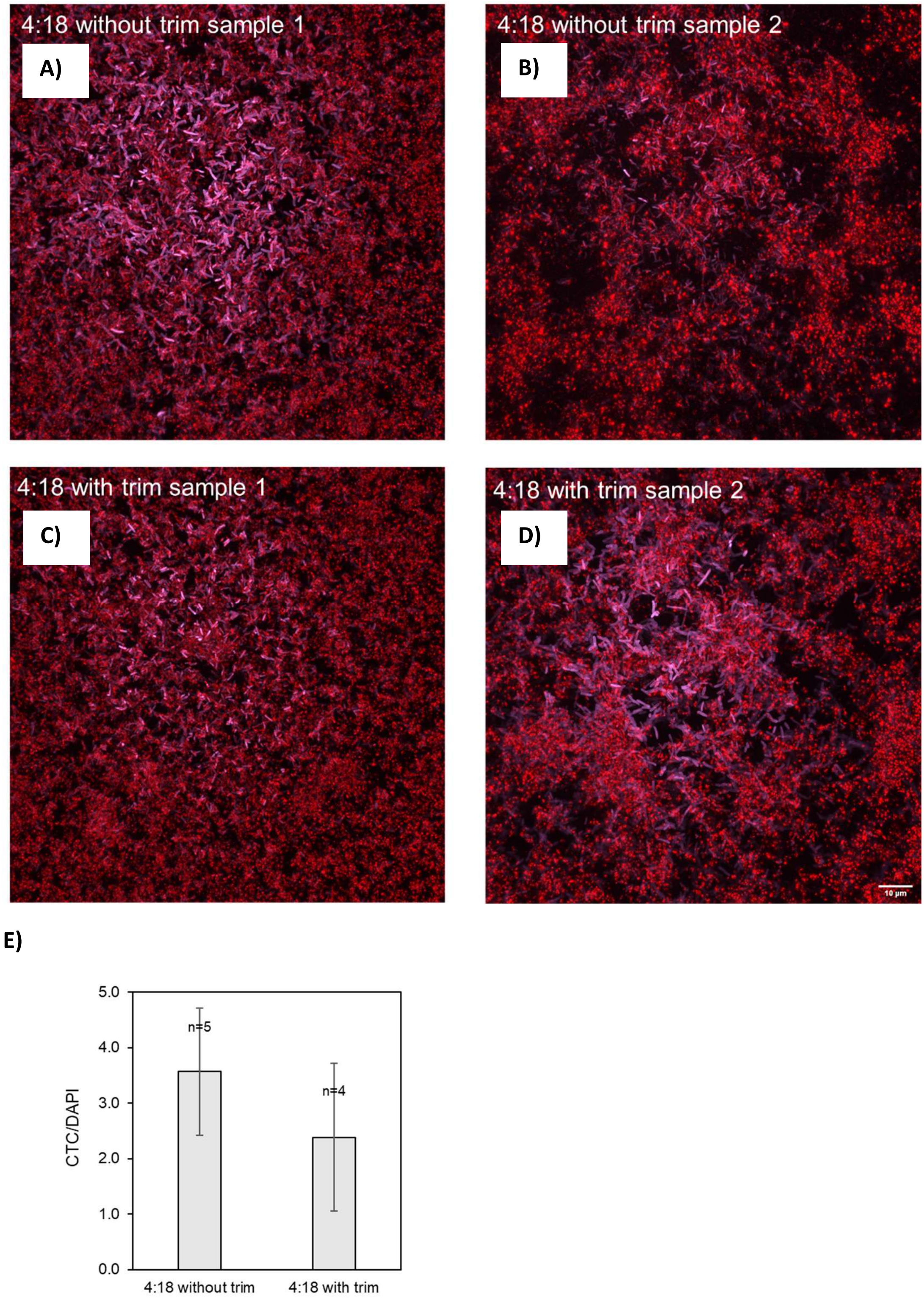

Supplementary Figure 13. Metabolic activity of Pseudomonas in absence and presence of trimethoprim as indicated by CTC (red) and DAPI (blue, counter) staining; Microscopy images A-B) replicate samples without trimethoprim, C-D) replicate samples with trimethoprim 25 mg/L. The same magnification was used in all images, scale bar represents 10 µm E) Quantification of staining ratio between CTC and DAPI staining n = number of images from 2 replicate samples.

Enlargements of microscopy images in Figure 8

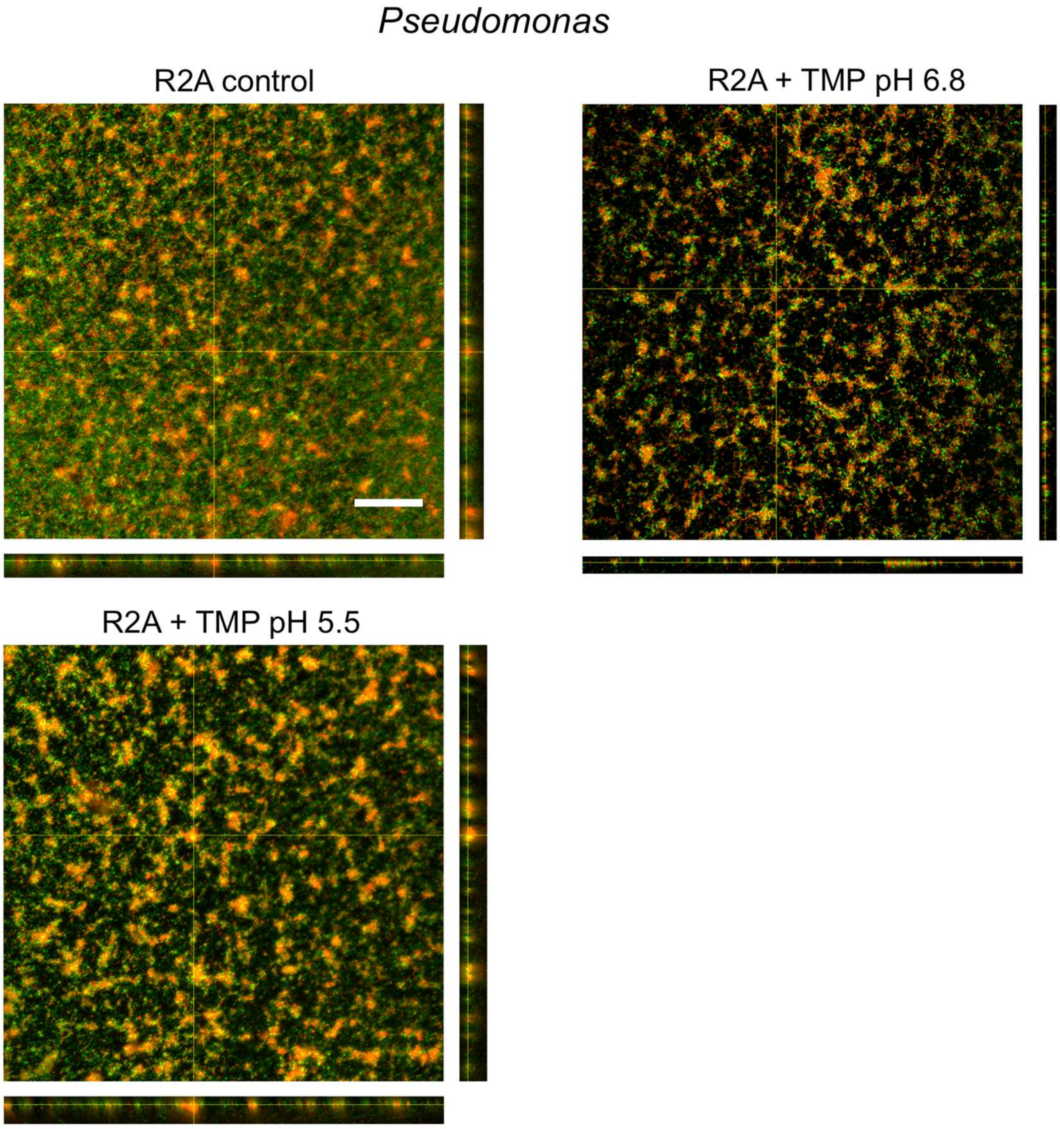

scale bar 100 μm

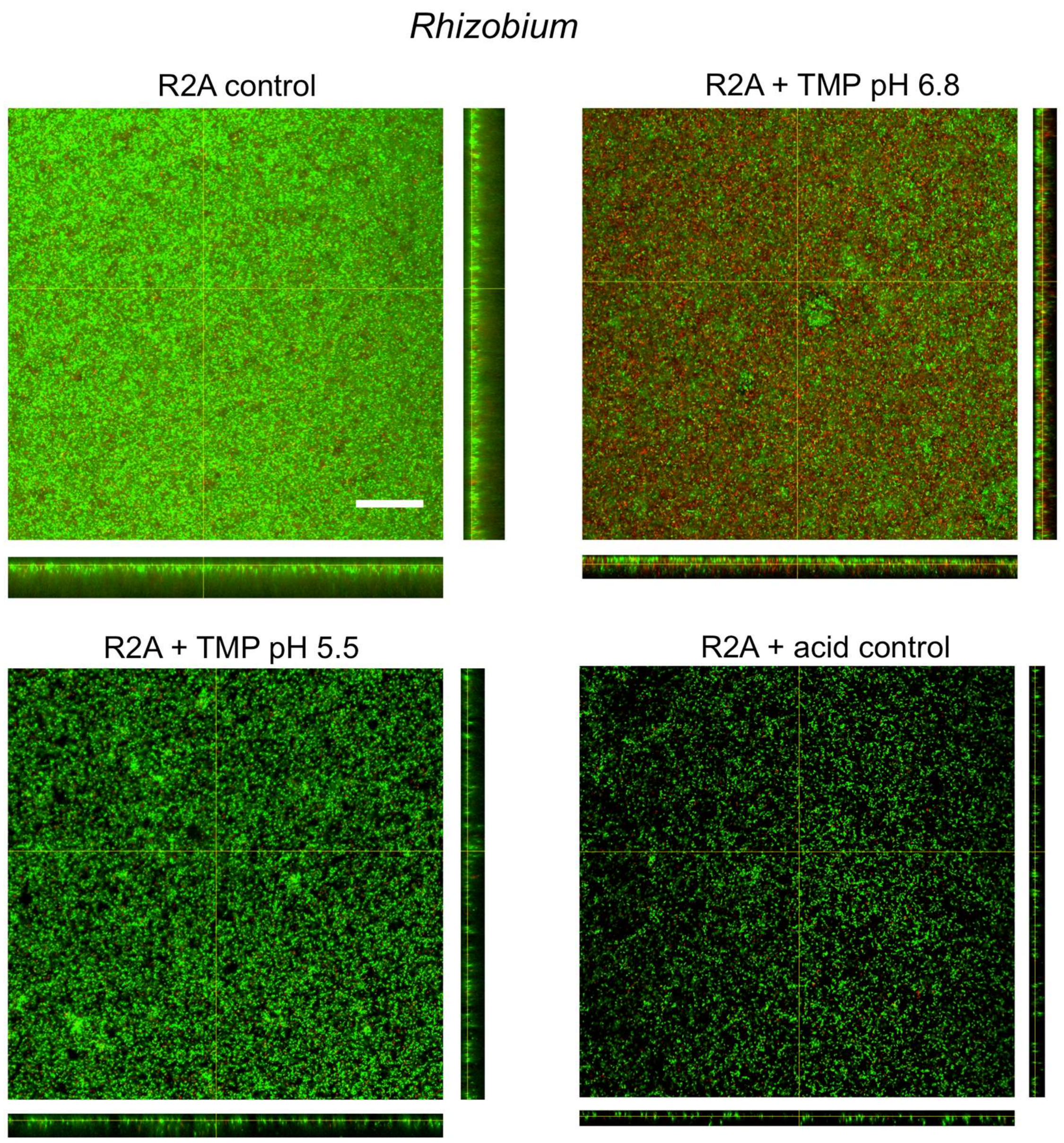

scale bar 100 μm

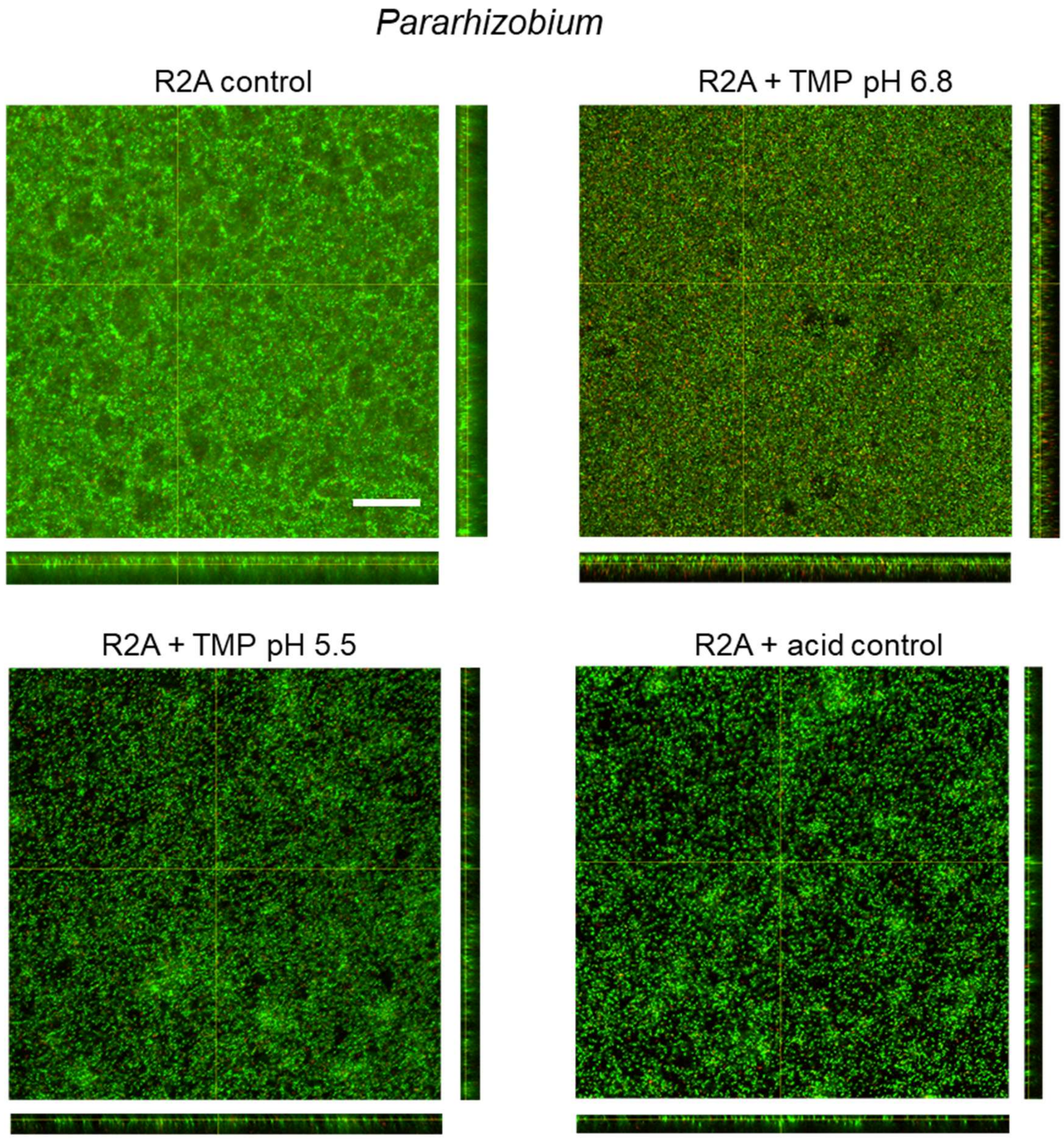

scale bar 100 μm

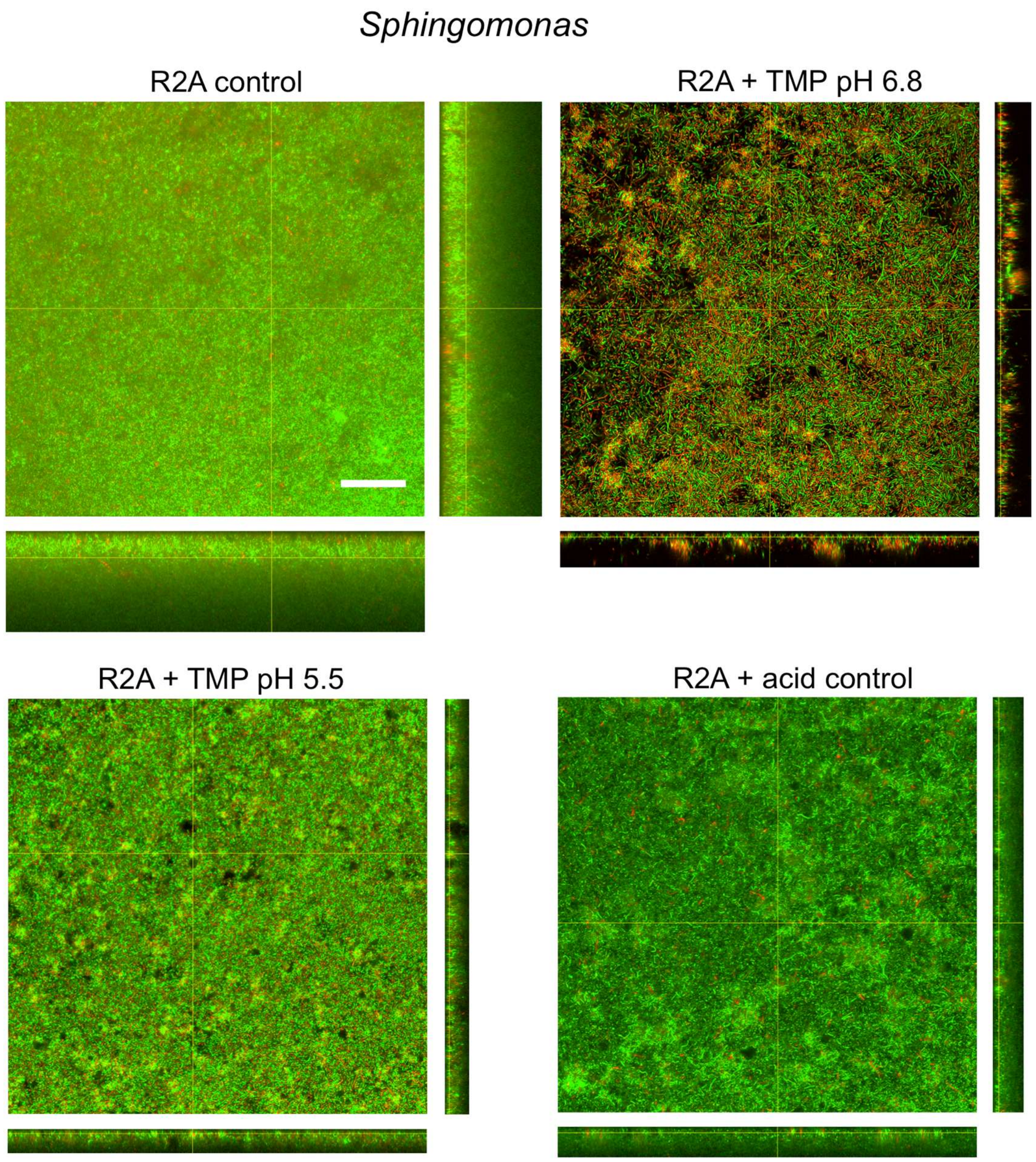

scale bar 100 μm

Supplementary Figure 14 Enlargements of microscopy images from Figure 8. All images collected with the same magnification (20x objective). Scale bar represent 100 μm. Please note: The gradient in stain with height of the biofilm observed in the Sphingomonas biofilm under control conditions is influenced by differences in the penetration of the light through the sample during microscopy analyses, as well as gradients in the ratio of water and highly hydrated EPS from the bottom of the biofilm out into the solution.

Cross assay - photos

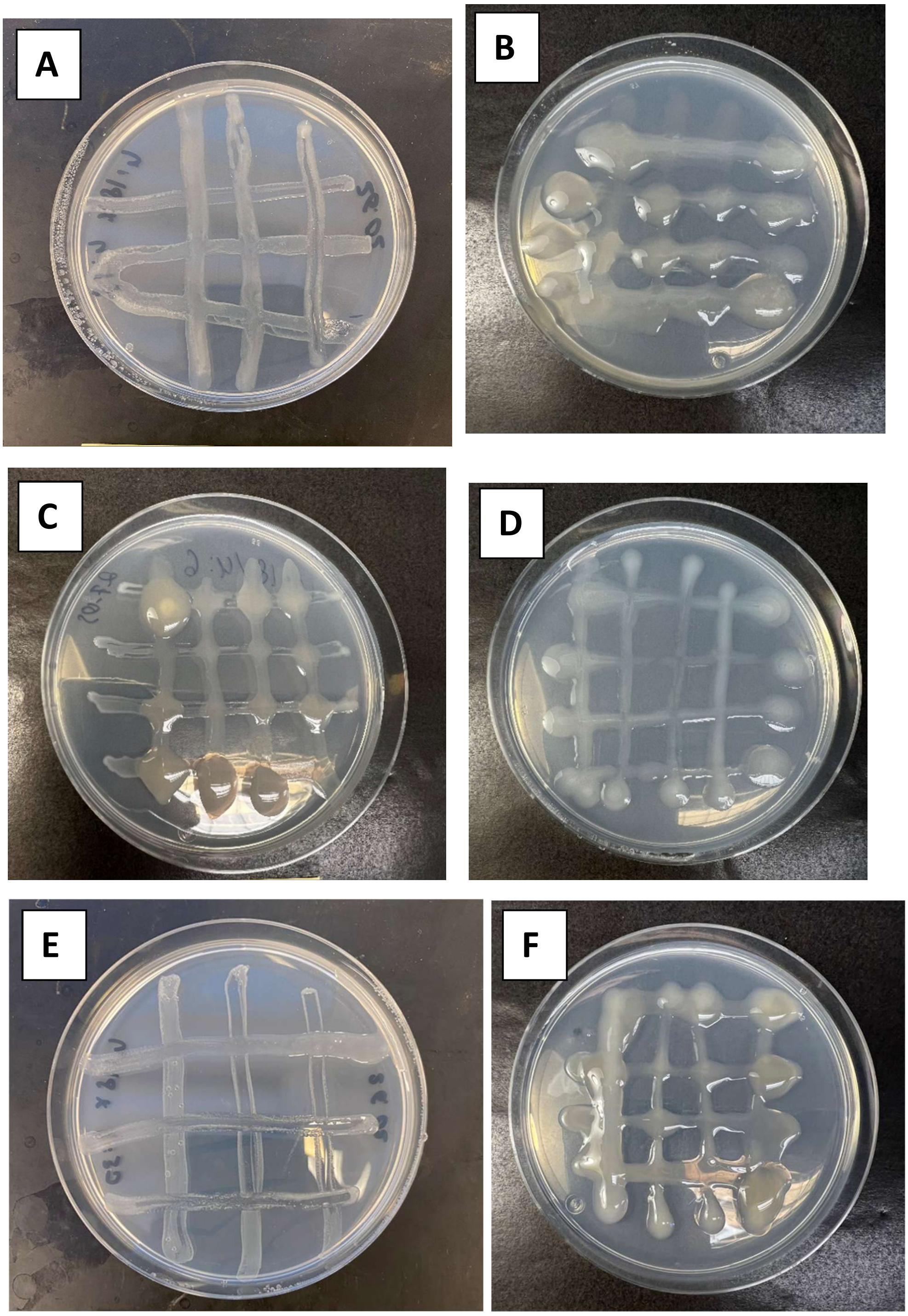

Supplementary Figure 15. Example of results from the cross assay showing absence of antagonism between bacteria isolates when grown on agar (R2A 10 %) plates.
A) Pseudomonas and Rhizobium. B) Pararhizobium and Sphingomonas.
C) Pseudomonas and Sphingomonas. D) Pararhizobium and Rhizobium.
E) Pseudomonas and Pararhizobium. F) Sphingomonas and Rhizobium

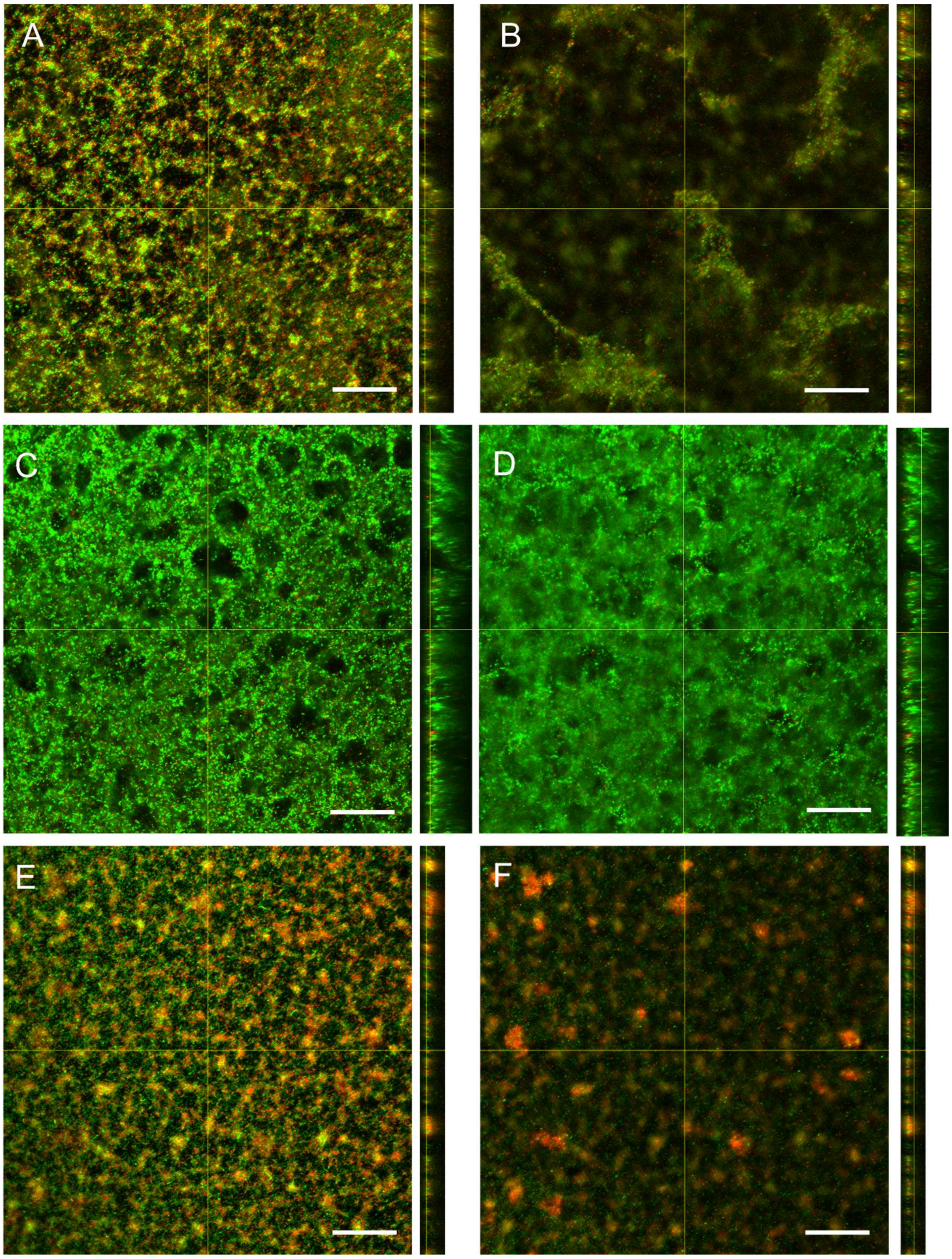

Supplementary Figure 16. Biofilms of Pseudomonas and Pararhizobium grown as A-B) co-culture, or C-F) individual species corresponding to C-D) Pararhizobium and E-F) Pseudomonas. Images A), C), E) correspond to a layer close to the bottom of the biofilm, and images B), D), F) correspond to a layer close to the top of the biofilm. Biofilm stained with BacLight staining. Scale bar 100 μm

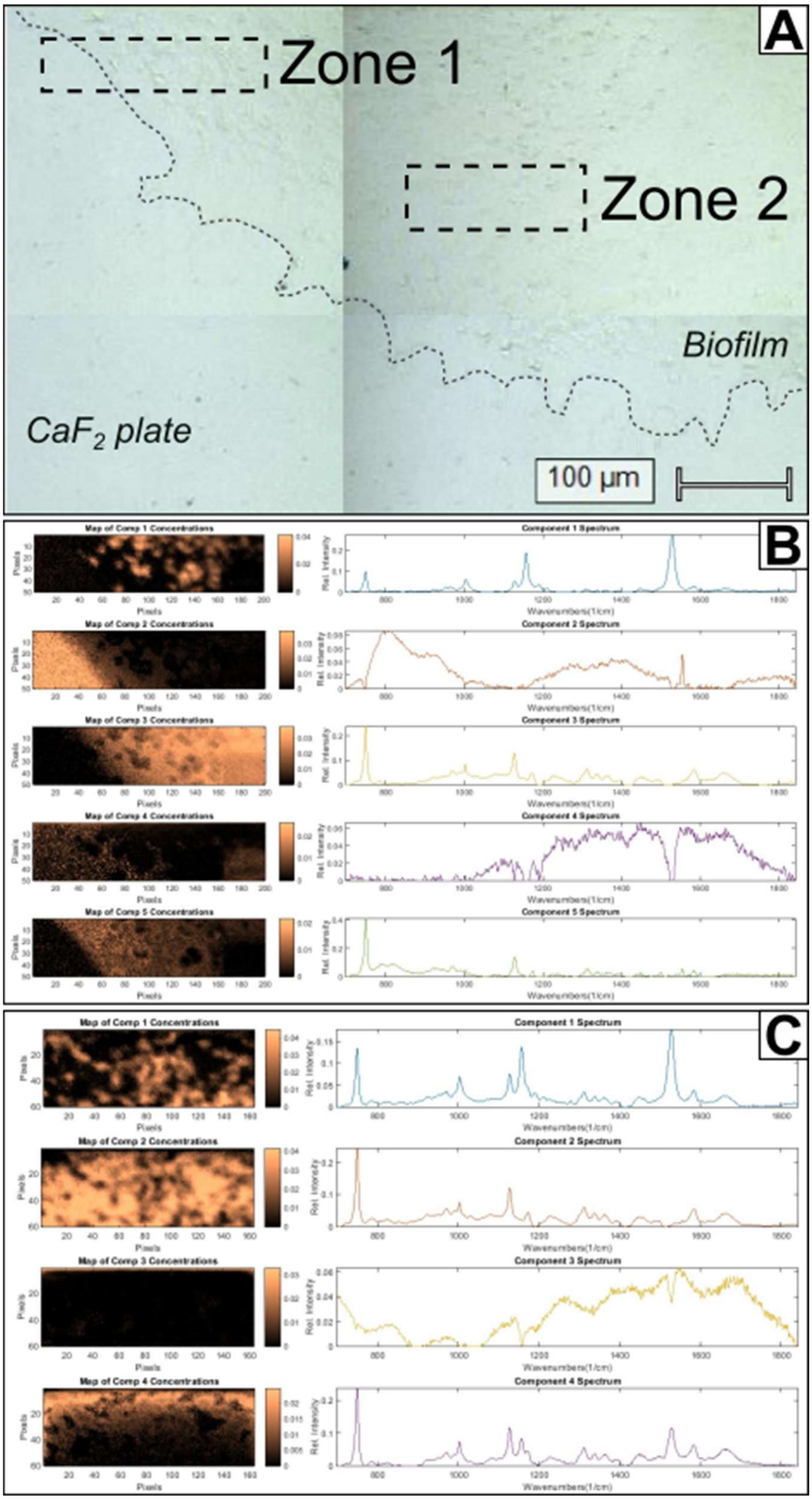

Supplementary Figure 17. Spectral components from MCR-ALS analysis of Raman hyperspectral images shown in Figure 11 of the manuscript. A) optical image. B) Spectral components and maps for Zone 1. Component 2 represents the CaF_2 slide and Component 4 noise. C) Spectral components and maps for Zone 2. Component 3 represents noise.

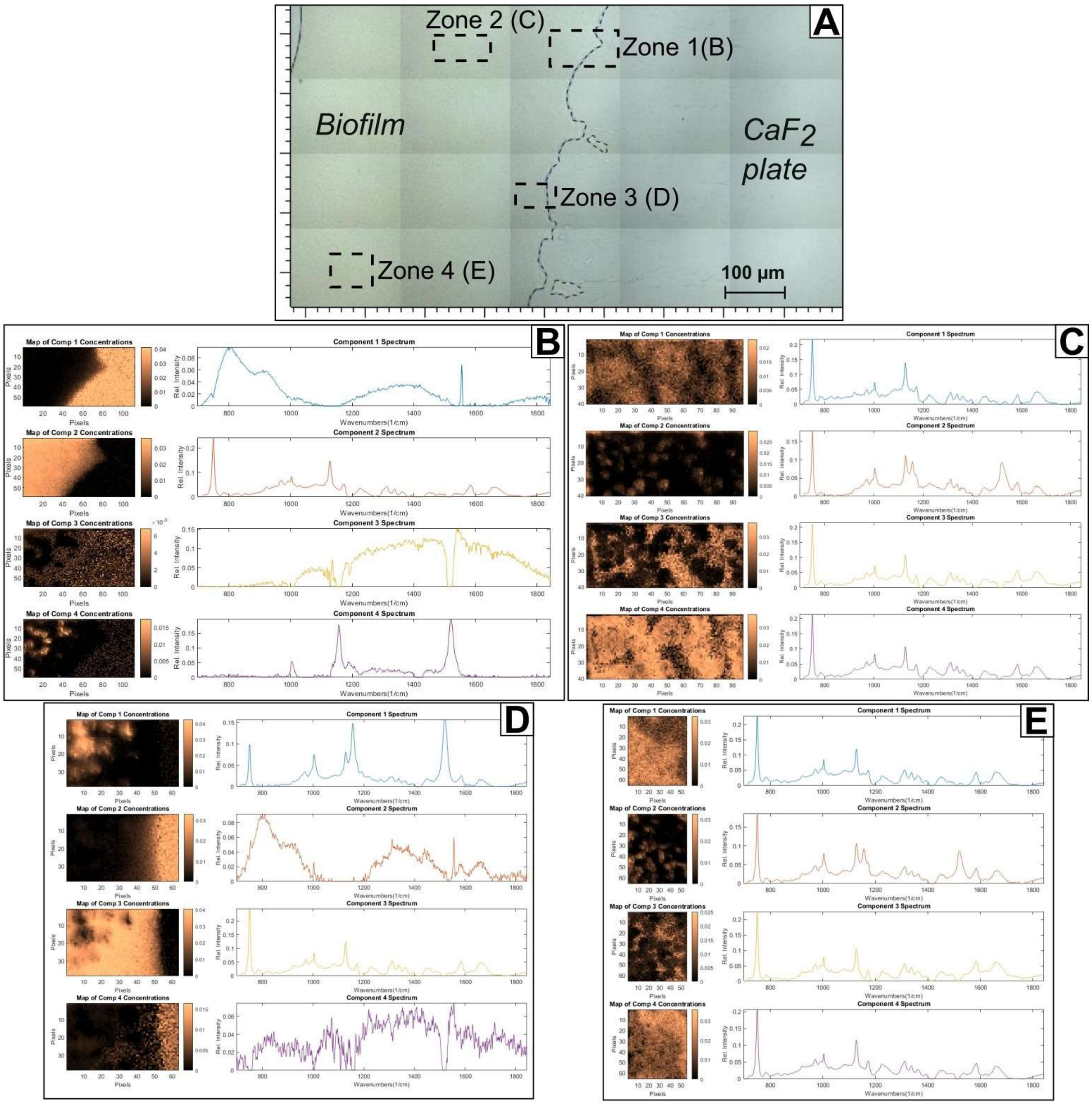

Supplementary Figure 18. Spectral components from MCR-ALS analysis of Raman hyperspectral images shown in Figure 13 of the manuscript. A) optical image. B) Spectral components and maps for Zone 1. Component 1 represents the CaF_2 slide, Component 2 cytochromes, Component 3 noise and Component 4 carotenoids. C) Spectral components and maps for Zone 2. Component 1 carotenoids, Component 2 the CaF_2 slide, Component 3 cytochromes and Component 4 represents noise. D) Spectral components and maps for Zone 3. Component 2 shows a region with carotenoids but also presence of cytochromes and the remining components all show cytochromes. E) Spectral components and maps for Zone 4. Component 2 shows a region with carotenoids but also presence of cytochromes and the remining components all show cytochromes. All zones were analysed using four components for consistency.
